# The *Listeria monocytogenes* Core-Genome Sequence Typer (LmCGST): a bioinformatic pipeline for molecular characterization with next-generation sequence data

**DOI:** 10.1186/s12866-015-0526-1

**Published:** 2015-10-22

**Authors:** Arthur W. Pightling, Nicholas Petronella, Franco Pagotto

**Affiliations:** Office of Analytics and Outreach, Center for Food Safety and Applied Nutrition, U.S. Food and Drug Administration, 5100 Paint Branch Parkway, College Park, MD 20740 USA; Biostatistics and Modelling Division, Bureau of Food Surveillance and Science Integration, Food Directorate, Health Products and Food Branch, Health Canada, 251 Sir Frederick Banting Driveway, Ottawa, K1A 0K9 ON Canada

**Keywords:** *Listeria monocytogenes*, Typing, Molecular characterization, Next-generation sequencing, Pulsed-field gel electrophoresis, PFGE, Multi-locus sequence typing, MLST, wgMLST, cgMLST

## Abstract

**Background:**

Next-generation sequencing provides a powerful means of molecular characterization*.* However, methods such as single-nucleotide polymorphism detection or whole-chromosome sequence analysis are computationally expensive, prone to errors, and are still less accessible than traditional typing methods. Here, we present the *Listeria monocytogenes* core-genome sequence typing method for molecular characterization. This method uses a high-confidence core (HCC) genome, calculated to ensure accurate identification of orthologs. We also developed an evolutionarily relevant nomenclature based upon phylogenetic analysis of HCC genomes. Finally, we created a pipeline (LmCGST; https://sourceforge.net/projects/lmcgst/files/) that takes in raw next-generation sequencing reads, calculates a subject HCC profile, compares it to an expandable database, assigns a sequence type, and performs a phylogenetic analysis.

**Results:**

We analyzed 29 high-quality, closed *Listeria monocytogenes* chromosome sequences and identified loci that are reliable targets for automated molecular characterization methods. We identified 1013 open-reading frames that comprise our high-confidence core (HCC) genome. We then populated a database with HCC profiles from 114 taxa. We sequenced 84 randomly selected isolates from the Listeriosis Reference Service for Canada’s collection and analysed them with the LmCGST pipeline. In addition, we generated pulsed-field gel electrophoresis, ribotyping, and *in silico* multi-locus sequence typing (MLST) data for the 84 isolates and compared the results to those obtained using the CGST method. We found that all of the methods yielded results that are generally congruent. However, due to the increased numbers of categories, the CGST method provides much greater discriminatory power than the other methods tested here.

**Conclusions:**

We show that the CGST method provides increased discriminatory power relative to typing methods such as pulsed-field gel electrophoresis, ribotyping, and multi-locus sequence typing while it addresses several shortcomings of other methods of molecular characterization with next-generation sequence data. It uses discrete, well-defined groupings (types) of organisms that are phylogenetically relevant and easily interpreted. In addition, the CGST scheme can be expanded to include additional loci and HCC profiles in the future. In total, the CGST method provides an approach to the molecular characterization of *Listeria monocytogenes* with next-generation sequence data that is highly reproducible, easily standardized, portable, and accessible.

**Electronic supplementary material:**

The online version of this article (doi:10.1186/s12866-015-0526-1) contains supplementary material, which is available to authorized users.

## Background

*Listeria monocytogenes* is a facultatively anaerobic, Gram-positive bacterium that occurs naturally in plant, soil, and surface water environments [1]. However, *L. monocytogenes* may also be isolated from domestic cattle, sheep, goats, and poultry [2] and can make its way into the food supply, causing sporadic and outbreak cases of foodborne listeriosis [3]. Listeriosis is commonly associated with life-threatening meningitis and septicemia in adults and may lead to miscarriages in pregnant women [4]. Thus, molecular characterization (typing) of *L. monocytogenes* is important to clinical microbiology and to the epidemiological analysis of listeriosis [5].

Molecular typing methods such as pulsed-field gel electrophoresis [6], ribotyping [7], and multi-locus sequence typing (MLST) [8] have provided greatly improved resolving power relative to previously used phenotypic methods, such as serotyping, phage typing, biotyping, antibiotic susceptibility testing, and bacteriocin typing [9]. Recent advances in DNA sequencing technologies and reduced costs have made high-quality whole-genome sequence (WGS) data readily available [10]. Comprehensive sequencing and analysis of bacterial genomes has been shown to be a valuable tool for epidemiological studies [11, 12]. In particular, WGS data are commonly used to identify nucleotide differences, so-called single-nucleotide polymorphisms (SNPs), between bacterial chromosomes [13]. However, studies have shown that the use of either *de-novo* or reference-guided assemblies for identifying SNP differences between subjects and references can lead to errors that make interpretation difficult [14–16]. Alternatively, WGS data may be assembled *de-novo* into chromosome sequence data that can be aligned and phylogenetically analyzed in order to compare them directly. However, we show that this method can generate misleading results, presumably due to differences between sequencing runs and/or the computational challenges of aligning short-read sequence data without a reference. Most importantly, the manner in which these methods are currently used ultimately relies upon the accurate interpretation of phylogenetic trees, a requirement that can make the assignment of discrete groupings (types) difficult and can make these approaches less accessible than the typing methods they are intended to replace.

Whole-genome multi-locus sequence typing (wgMLST) has been shown to be a powerful alternative to SNP or whole-chromosome analyses [17] that is amenable to the assignment of discrete groupings in addition to phylogenetic analysis. In concept, this method is nearly identical to the MLST approach, during which 7 or so loci are studied, with the exception that all the genes in a genome are examined. This scheme takes advantage of the discriminating power of WGS data while providing the basis for the grouping of organisms into sequence types. wgMLST also makes the interpretation of data easier and more accessible. However, the accurate identification of orthologous sequences is computationally expensive and there is no current consensus on how this should be accomplished, making wgMLST methods difficult to standardize and distribute. Researchers have shown that using the loci that comprise an organisms core-genome (cgMLST) provides a powerful means of analyzing WGS data that can be standardized [18, 19]. While cgMLST is an improvement over wgMLST, both methods, like standard MLST, rely upon allele numbering systems that often provide scant information regarding the evolutionary relationships of organisms.

Here, we present a cgMLST-style typing method for the molecular characterization of *L. monocytogenes* (core-genome sequence typing; CGST) that has several advantages over other proposed methods. In order to ensure that orthologs are properly identified, we calculated a high-confidence core (HCC) of genes that is useful for reliable and efficient large-scale typing. Furthermore, we developed a nomenclature that is based upon nucleotide distances between HCC profiles, providing phylogenetically meaningful groupings. Finally, we wrote a bioinformatic pipeline (LmCGST available at https://sourceforge.net/projects/lmcgst/files/) that can: i) analyze raw reads, contiguous sequences, or closed chromosome sequences; ii) identify an organism’s HCC profile; iii) compare that profile to an expandable database; iv) provide an evolutionarily meaningful sequence type assignment; and v) generate a phylogenetic analysis that illustrates the evolutionary relationship of the subject to the members of the database. In total, the CGST method provides an approach that is highly reproducible, easily standardized, portable, and accessible.

## Results and discussion

### Calculation of the *Listeria monocytogenes* core-genome

In order to calculate the pan- and core-genomes of *Listeria monocytogenes*, we used a semi-automated approach in which protein sequence translations of open reading frames (ORFs) predicted from a set of 29 high-quality, phylogenetically diverse chromosome sequences obtained from the National Center for Biotechnology Information (NCBI) were analyzed (Additional file 1, red labels and Additional file 2). A pan-genome is defined as the total pool of ORFs present within the genomes examined (*i.e.,* the union of ORFs) and a core-genome is defined as the subset of ORFs that are present within every genome (*i.e.,* the intersection of ORFs) [20–22]. Based upon pairwise comparisons with the Basic Local Alignment Search Tool (BLAST) [23], using a protein sequence similarity cut-off of 60 % and a minimum coverage of 80 % [24], we determined that the *L. monocytogenes* pan- and core-genomes consist of 4766 and 2114 ORFs, respectively (Additional file 3). Our results are consistent with the analysis of 17 *L. monocytogenes* genomes performed by Kuenne, et al. (Additional file 3, diamond) [24].

### Calculation of a high-confidence core

During the course of calculating the *L. monocytogenes* pan- and core-genomes, we identified conditions that confound pairwise homology searches, such as loci that retrieve multiple hits (*i.e.,* multi-copy genes), the use of different ORF prediction software, and the presence of low complexity regions within ORFs. Therefore, we further curated the set of 2114 core ORFs as described below in order to develop a robust database, ensuring that all orthologs used for downstream comparisons are properly identified. We found and removed 439 predicted multi-copy ORFs. In addition, we annotated the 29 high-quality, closed genome sequences obtained from NCBI with GeneMark [25], Glimmer [26, 27], and Prokka v1.10 [28]. We then used these datasets to estimate three different single-copy core-genomes that are composed of 1772, 1438, and 1826 ORFs, respectively. Differences in the numbers of ORFs predicted may occur as programs use a variety of methods to identify start/stop codons and overlapping ORFs [29]. We performed an all *versus* all bidirectional protein sequence BLAST to identify 1061 ORFs that are shared in all four datasets. Then, we performed an all *versus* all bidirectional nucleotide BLAST with 114 taxa in order to identify ORFs that fail to generate hits (likely due to the presence of low complexity regions) or generate ambiguous results. Any ORF that did not generate hits to all other taxa when used as a query were removed from the analysis. This resulted in the removal of an additional 48 ORFs. In total, 1013 ORFs were identified (Additional file 4) that are reliable targets of nucleotide and protein sequence BLAST searches and, so, comprise hour high-confidence core (HCC) of predicted genes.

In order to characterize the HCC, we assigned Clusters of Orthologous Groups (COGs) categories to the full set of strain 08–5578 protein sequences [GenBank: NC_013766] and compared it to the suite of predicted proteins in the HCC (Additional file 5). The distributions of both the full-set of proteins and the HCC-set within COGs categories are similar. This result indicates that the HCC provides a good approximation of the diversity of functions present in the full complement of *L. monocytogenes* genes. We also mapped the strain 08–5578 HCC genes and the HCC loci appear to be evenly distributed throughout the chromosome (Additional file 6).

### Core-genome sequence typing of *Listeria monocytogenes*

We developed a bioinformatic pipeline using the Perl programming language (LmCGST.pl, available at https://sourceforge.net/projects/lmcgst/files/) for identifying HCC loci in genome sequence data and comparing a subject HCC to a database that has been seeded with HCC profiles calculated from 114 unique, high-quality chromosome sequences (Additional file 7). The script can take raw short-read sequence data (*i.e.,* fastq files), contiguous sequences, closed chromosome sequences, or fully annotated ORFs as input. The software compares the subject HCC to the database and identifies the HCC profile with the fewest nucleotide differences. A file is also generated that reports on the numbers of perfect ORF matches, partial ORF matches, missing ORFs, and the numbers of SNPs, along with the identities of SNP-containing ORFs and the positions of the SNPs.

**Fig. 1:**
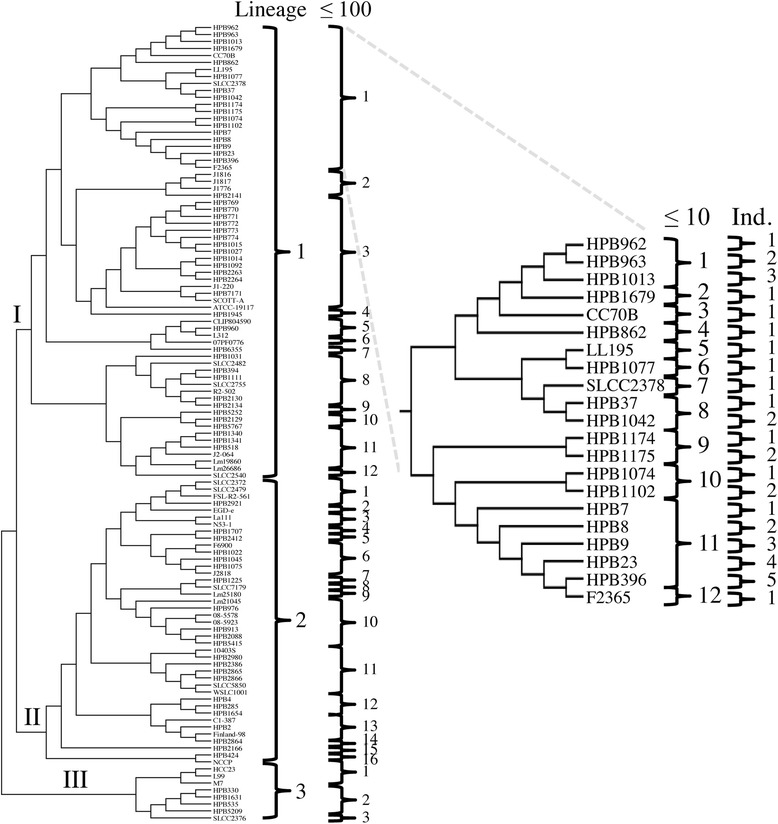
*Listeria monocytogenes* high-confidence core profiles grouped by nucleotide distances. A cladogram was calculated by aligning and concatenating 1013 loci that comprise the *L. monocytogenes* high-confidence core (HCC) genomes of 114 taxa and analyzing the resulting alignment of 1,067,173 nucleotide positions with the Randomized Axelerated Maximum Likelihood tool (GTRCATI + 25γ). The best of 100 bootstrap replicates is shown. Nucleotide distances were measured with PHYLIP. Taxa were grouped by evolutionary lineage (I, II, or III) and those that have 100 and 10 or fewer nucleotide differences, while unique HCC profiles that differ by no more than 10 nucleotides were numbered in the order that they were processed

A phylogenetically relevant typing scheme was developed by grouping HCC profiles by evolutionary lineages (Fig. 1). Then we calculated the pairwise nucleotide distances between HCC profiles of each member of the database. We identified and grouped those HCC profiles with less than or equal to 100 base-pair differences and those with less than or equal to 10 base-pair differences. Finally, unique HCC profiles that differ by fewer than 10 nucleotides were numbered in the order that they were processed. A core-genome sequence type is, therefore, defined as a unique set of HCC sequences and subjects must be 100 % identical at the nucleotide level with no missing or partial ORFs to be considered a match to any HCC profile in the database. This typing scheme allows for the assignment of unique identifiers that specify the evolutionary relationships of subjects to members of the database within the *L. monocytogenes* phylogeny (Additional file 8). Finally, a phylogenetic analyses of the HCC profiles of the subject and the database are generated in order to visualize their evolutionary relationships (Additional file 9).

In order to establish the levels of genome sequence coverage necessary for accurate core-genome sequence typing, we tested the pipeline with a set of 12 *L. monocytogenes* strain 08–5578 Illumina short-read sequence datasets obtained from sequencing runs of varying qualities (Additional file 10 and Additional file 11). We found that *de-novo* sequence assemblies of at least 66-fold coverage provide reliable results, with no false SNPs, missing ORFs, or partial ORFs.

In order to predict the amount of time required to run the LmCGST pipeline as the size of the database increases, we documented the amount of time necessary for the pipeline to complete the genome assembly, annotation, HCC comparisons, and phylogenetic analyses for databases containing 25, 50, and 100 randomly selected HCC profiles (Additional file 12). In addition, we estimated the amount of time required to assemble and annotate a single genome. Genome assembly with SPAdes takes approximately 2.69 h, while it takes approximately 0.09 h to perform annotation with Prokka. Although these times may change with different genomes and short-read sequence datasets, they did not increase with the size of the database (Additional file 13). For the HCC comparison and optional tree-building steps, processing times did increase with the size of the database. We fitted the amount of time necessary to perform the HCC comparisons and phylogenetic analyses to a polynomial regression model (y = 0.0015x^2^ + 2.027x-6.4733 and y = 0.0631x^2^ + 9.973x + 33.137, respectively). With 100 taxa occupying the database, the comparison step took approximately 0.059 h and the tree-building step took approximately 0.46 h. When we predicted processing times with 500 taxa present in the database, we estimated that it will take approximately 0.38 h for the comparison step and 5.77 h to estimate the phylogeny. The total amount of time that is predicted to be necessary for the LmCGST pipeline to run with a database of 500 HCC profiles is 8.93 h, with the assembly, annotation, and comparison steps accounting for approximately 3.16 h.

### Comparison of molecular typing methods

**Fig. 2:**
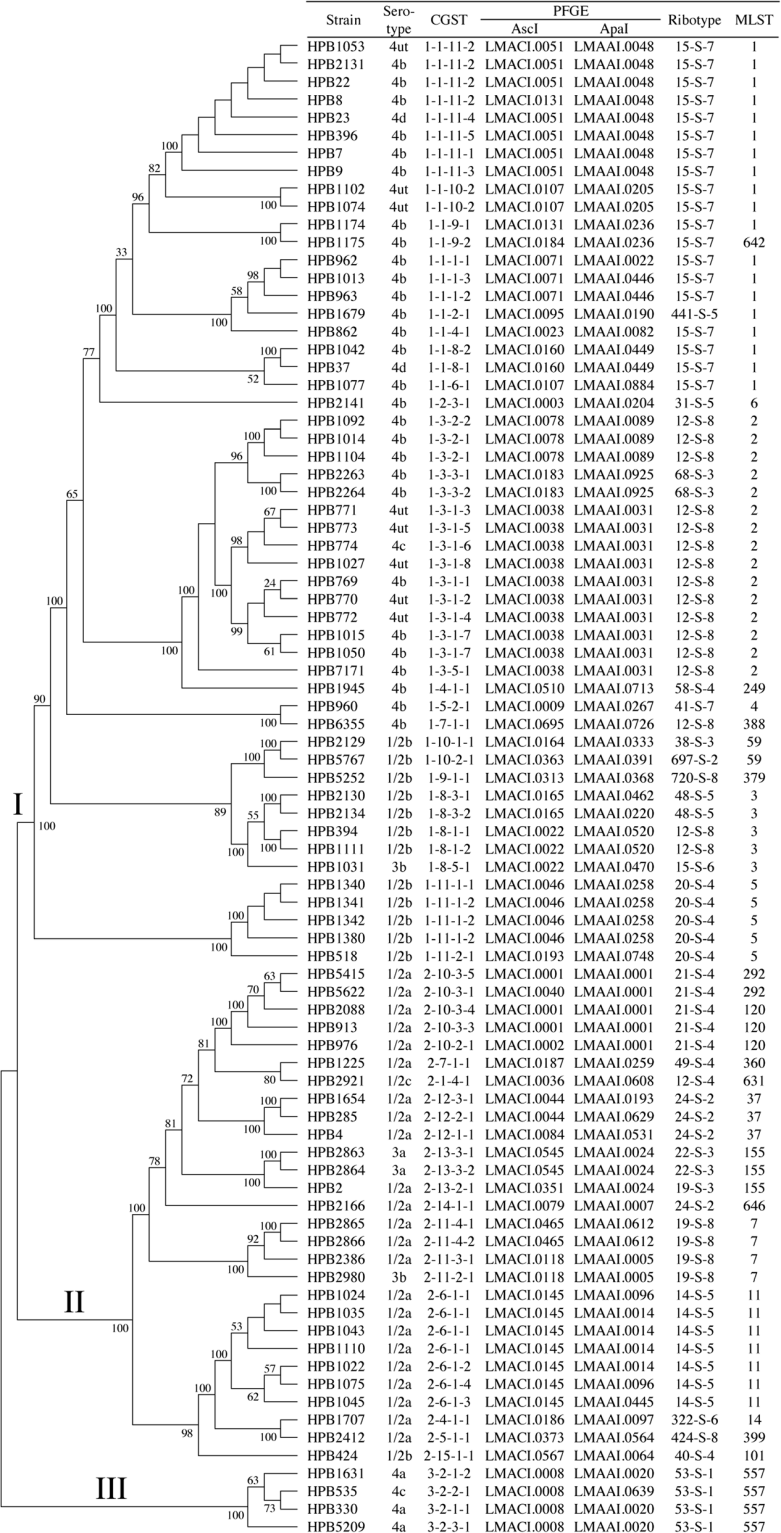
Typing data derived from 84 *Listeria monocytogenes* strains. Strains were selected randomly from the collection stored at the Listeriosis Reference Service for Canada. Standard typing assays, such as serotyping, AscI and ApaI pulsed-field gel electrophoresis (PFGE), and ribotyping were performed. In addition, whole-genome sequence data were generated and analyzed with *in silico* multi-locus sequence typing (MLST) and core-genome sequence typing (CGST)

In order to compare the core-genome sequence typing (CGST) method to currently used molecular typing methods, we randomly selected 84 strains from the collection at the Listeriosis Reference Service for Canada. Then, we performed pulsed-field gel electrophoresis (PFGE) [30] with both ApaI and AscI restriction enzymes and ribotyping [7]. In addition, we generated whole-genome sequence data with an Illumina MiSeq benchtop sequencer and performed *in silico* multi-locus sequence typing (MLST) [8] with *abcZ, bglA, cat, dapE, dat, ldh,* and *lhkA* loci [31] and CGST (Fig. 2). Previous studies have shown that *in silico* MLST analyses of next-generation sequence data allow for high levels of allelic identification and are highly-concordant with published and publicly available sequence types [32]. We analyzed the congruence and discriminating power of each typing method individually and in two combinations in which we analyzed ApaI and AscI PFGE together (labelled PFGE) and we analyzed ApaI, AscI, and ribotyping together (labelled PFGE + Ribo). We began by calculating the Simpson’s index of diversity [33] with 95 % confidence intervals (Additional file 14). The values for all categories range from 0.889 (Ribotyping) to 0.995 (CGST) and the data indicate that CGST has higher discriminating power than the other typing methods, either individually or combined. In addition, the data indicate that the strains randomly selected for this study are sufficiently diverse for purposes of comparing these different typing methods.

We then calculated the adjusted Wallace coefficients [34, 35] with 95 % confidence intervals [36] for the typing datasets (Additional file 15 and Table 1). The data indicate that the typing methods tested here are fairly congruent. For example, if two strains are identified as belonging to the same group using the CGST method, there is approximately a 65.7 % chance that they will be grouped together with PFGE or PFGE + Ribo and a 100.0 % chance they will be grouped together with ribotyping or MLST methods (Table 1, first row). The data also indicate that, due to the increased number of categories, the CGST method has greater discriminatory power than the other methods tested here. That is, if two strains are identified as belonging to the same group with either PFGE or PFGE + Ribo, there is a 12.0 % chance that they will be grouped together with the CGST method, while ribotyping and MLST methods yielded values of 4.6 and 4.8 %, respectively (Table 1, first column).

**Table 1:** Adjusted Wallace coefficient and 95 % confidence intervals

	CGST	PFGE + Ribo	PFGE	ApaI	AscI	Ribotype	MLST
CGST		0.657 (0.400–0.914)	0.657 (0.400–0.914)	0.828 (0.645–1.000)	0.827 (0.644–1.000)	1.000 (1.000–1.000)	1.000 (1.000–1.000)
PFGE + Ribo	0.120 (0.019–0.222)		1.000 (1.000–1.000)	1.000 (1.000–1.000)	1.000 (1.000–1.000)	1.000 (1.000–1.000)	0.977 (0.955–0.999)
PFGE	0.120 (0.019–0.222)	1.000 (1.000–1.000)		1.000 (1.000–1.000)	1.000 (1.000–1.000)	1.000 (1.000–1.000)	0.977 (0.955–0.999)
ApaI	0.128 (0.024–0.233)	0.845 (0.743–0.947)	0.845 (0.743–0.947)		0.844 (0.741–0.947)	0.980 (0.962–0.999)	0.931 (0.896–0.967)
AscI	0.118 (0.017–0.220)	0.781 (0.711–0.851)	0.781 (0.711–0.851)	0.780 (0.709–0.850)		0.982 (0.965–0.999)	0.982 (0.965–0.999)
Ribotype	0.046 (0.000–0.095)	0.251 (0.137–0.365)	0.251 (0.137–0.365)	0.291 (0.171–0.412)	0.316 (0.207–0.425)		0.787 (0.641–0.932)
MLST	0.048 (0.000–0.099)	0.256 (0.144–0.368)	0.256 (0.144–0.368)	0.289 (0.168–0.410)	0.329 (0.223–0.436)	0.820 (0.683–0.957)	

The Wallace coefficient measures agreement between groupings made with different typing methods. Row headers indicate methods from which two random samples were drawn and column headers identify the methods that were compared. The probabilities that two samples grouped together with one method (rows) will also be grouped together with another method (columns) are shown along with 95 % confidence intervals (parentheses)

ApaI and AscI data were combined to generate the PFGE category and ApaI, AscI, and Ribotype data were combined to generate the PFGE + Ribo category

## Conclusions

Here, we have calculated a *Listeria monocytogenes* high-confidence core (HCC) genome which serves as the basis for an extended multi-locus sequence typing regime called core-genome sequence typing (CGST). We have shown that analysis of next-generation sequence data with CGST provides significantly increased power to distinguish isolates of *L. monocytogenes* relative to currently used methods of molecular characterization. Furthermore, CGST provides several advantages over other typing methods that utilize next-generation sequence data, such as the analysis of single-nucleotide polymorphisms (SNPs) or phylogenetic analysis of whole-chromosome sequence data. Recent studies have shown that SNP analyses can be problematic and can result in phylogenetic artifacts that may obscure the true relationships between isolates, whether a *de-novo* or reference-guided approach is used [14–16]. Theoretically, simply performing phylogenetic analysis of whole-chromosome sequence data should provide the highest levels of resolution. However, differences between next-generation sequencing runs may introduce errors that make chromosome sequences appear more evolutionarily distant than they really are (Additional file 16 and Additional file 17). That is, phylogenetic analyses may yield branches separating taxa, due to differences between sequencing runs, when no differences between chromosomes actually exist. Although, it is possible that high sequence coverage (*e.g.,* 155.48-fold) may reduce the numbers of differences, the CGST method is capable of accurately resolving relationships with much lower levels of sequence coverage (Additional file 10). Furthermore, accurate alignment and phylogenetic analysis of large numbers of whole-chromosomes is computationally expensive and accessible methods are currently lacking. Finally, both SNP and whole-chromosome analyses ultimately rely upon the interpretation of phylogenetic trees while keeping all of these considerations in mind. While methods that utilize next-generation sequence data can deliver increased resolution, they may lack discrete, well-defined types and may be less accessible than the typing methods they are meant to replace.

The aim of developing the extended MLST scheme presented here is to remedy the shortcomings of other methods of molecular characterization that utilize next-generation sequence data by providing discrete, well-defined groupings (types) of organisms that are phylogenetically relevant and easily interpreted. In addition, because we target 1013 HCC loci retrieved from *de-novo* genome assemblies, only the most reliable portions of chromosome sequence assemblies are used; regions that are common sources of error, such as gaps and repetitive regions, are avoided [37]. An additional, significant benefit of the CGST scheme is that it can be expanded in the future to include multi-copy and accessory genes, as necessary or desired, and studies correlating nucleotide differences between loci with important phenotypes can be incorporated. Furthermore, the database can be continually improved with the addition of novel HCC profiles. Thus, the CGST provides the best of next-generation sequence data analysis while avoiding several sources of error.

## Methods

### Calculation of the *Listeria monocytogenes* core-genome

We downloaded chromosome, gene, and protein sequence data for 29 high-quality, closed *Listeria monocytogenes* chromosome sequences from the National Center for Biotechnology Information (Additional file 2) [38–48]. Then, we used the methods of Kuenne et al. [24] to calculate the *L. monocytogenes* pan- and core-genomes. Briefly, we used the Basic Local Alignment Search Tool (BLAST) [23] to establish orthology by performing pairwise protein sequence alignments (*i.e.,* all *versus* all BLASTp) with a minimum coverage threshold of 80 % and an identity cut-off of 60 % [24]. Sequences encoding proteins present in all 29 datasets were counted as members of the core-genome and the entire collection of sequences constitutes the pan-genome. Pan- and core-genome distributions were calculated by randomly selecting between 2 and 28 taxa randomly, with replacement and calculating 1000 times (Additional file 3).

### Calculation of a high-confidence core

The high-confidence core (HCC) was calculated by identifying and removing open-reading frames from the calculated *Listeria monocytogenes* core-genome whose products yielded multiple hits with the all *versus* all pairwise alignment analysis of the 29 datasets. We then re-annotated the chromosome sequences three times with GeneMarkS v2.8 [25], Glimmer v3.02 [26, 27], and Prokka v1.10 [28]. Using BLASTp with the 80 % coverage, 60 % identity cut-offs, we identified sequences present in all four datasets (NCBI, GeneMark, Glimmer, and Prokka). Finally, we performed an all *versus* all BLASTn analysis in order to identify genes that, when used as a query, reliably retrieve (“hit”) homologous nucleotide sequences. The composition of HCC loci were compared to the total complement of genes present in the *L. monocytogenes* strain 08–5578 genome the by using the Clusters of Orthologous Genes database (“2003 COGs, original format”) available at http://www.ncbi.nlm.nih.gov/COG/ [49, 50] to analyze the calculated 08–5578 HCC and the NCBI annotation.

### DNA extraction, library construction, and DNA sequencing

*Listeria monocytogenes* isolates frozen in glycerol were streaked on pre-warmed Tryptose Agar plates and incubated at 37 °C over-night. Single colonies were picked and used to inoculate 5 ml pre-warmed Brain Heart Infusion (BHI) broth and incubated over-night at 37 °C with shaking (200 rpm). Then, 200 μl of the cultures were transferred to 50 ml pre-warmed BHI and incubated at 37 °C with shaking for 6 h to achieve mid-logarithmic growth phase [51, 52]. Approximately 25 ml of the cultures were decanted into 50 ml falcon tubes and centrifuged at 3800 RCF for 5 min. The pellets were completely dissolved in 500 μl Tris-ethylenediaminetetraacetic acid by vortexing. We added 500 μl phenol-chloroform (1:1), 30 μl sodium acetate (3 M, pH 5.2), and 30 μl sodium dodecyl sulfate and mixed vigorously by shaking. The mixtures were then pipetted into 2 ml screw-cap tubes filled with approximately 0.5 ml glass beads (0.1 mm). The tubes were shaken in a Mini-Beadbeater machine (BioSpec products, Bartlesville, Oklahoma) for 45 s using the “Homogenizer” setting and placed on ice for 45 s. Shaking was repeated an additional four times. Approximately 300 μl of the mixtures were then added to Maxwell 16 Cell DNA Purification Kit cartridges and samples were run using the standard DNA Blood/Cells protocol on a Maxwell 16 machine (Promega, Madison, Wisconsin) with elution in 300 μl nuclease-free water. RNA contamination was removed by adding 2 μl RNase A (Qiagen Sciences, Maryland) and incubating the samples for 10 min at 37 °C. Single phenol-chloroform-isoamyl alcohol (25:24:1) extractions followed by two ethanol precipitations were done. Samples were indexed with Nextera XT DNA Sample Preparation Kits (Illumina, San Diego, California) according to the standard protocol and sequenced (2 × 250 bp reads) on a MiSeq benchtop sequencer (Illumina).

For the *L. monocytogenes* strain 08–5578 test dataset, the sample was split into four subsamples. Each subsample was indexed and sequenced as described above three separate times for a total of twelve sets of short-read sequences. These data have been deposited to the National Center for Biotechnology Information (NCBI) Sequence Read Archive (SRA) under accession numbers SRR1342176, SRR1342220, SRR1373524, SRR1373525, SRR1373527, SRR1373529, SRR1373530, SRR1373531, SRR1373534, SRR1373535, SRR1507228, and SRR1508282.

### Core-genome sequence typing of *Listeria monocytogenes*

A Perl script was developed (LmCGST) that takes raw short-read (fastq), contiguous, or chromosome sequence files, identifies the high-confidence core (HCC) genome of a subject, compares the subject HCC profile to the database of HCC profiles, and generates a phylogenetic tree to illustrate the relationships of the subject to members of the database. If fastq files are provided, LmCGST assembles the reads *de-novo* using SPAdes v3.0.0 [53] and the BayesHammer error correction tool [54]. The resulting contiguous sequences are then annotated with Prokka v1.10 and the HCC loci are identified with bidirectional BLAST. Assemblies yielding partial (*i.e.,* less than 60 %) or missing ORFs will generate a warning as sequence quality may be insufficient for genome-scale typing. Phylogenetic analyses are calculated with PHYLIP by first using the “dnadist” module to calculate distances (F84 model and default settings) and then by using the “neighbor” module (Neighbor-joining with default settings) to generate the tree.

We used this script and a database originally seeded with HCC profiles calculated from 29 high-quality, closed genome sequences (Additional file 2) to analyze additional sequence data obtained from NCBI as well as data whole-genome sequence data generated as described above. A total of 14 completely unique HCC profiles calculated from the data were added to the database (Additional file 18). We also calculated HCC profiles using sequence data from 84 strains from the Listeriosis Reference Service for Canada's collection; [55] 71 strains were identified as having unique HCC profiles and were added subsequently added to the database (Additional file 18), while HCC profiles calculated from the remaining 13 datasets matched an HCC profile already populating the database (Additional file 18, asterisks).

The pipeline was run on a desktop computer with an AMD Phenom II X6 1090 T processor and 16GB of DDR3 RAM using 5 cores.

### Comparison of molecular typing methods

Serotyping of *Listeria monocytogenes* strains was performed using commercial O-antigen *Listeria* antisera (Denka Seiken, Tokyo, Japan), according to the manufacturer’s recommendations. Consistent with PulseNet standardized protocols, isolates were subtyped using pulsed-field gel electrophoresis with AscI and ApaI restriction endonucleases, as previously described [56]. Automated ribotyping was performed with the restriction enzyme EcoRI and the RiboPrinter Microbial Characterization System (Qualicon Inc., Wilmington, Delaware), according to the manufacturer’s manual [57]. Multi-locus sequence typing was performed *in silico* using allele sequences and profiles downloaded from the Pasteur Institute (http://bigsdb.web.pasteur.fr/), and a custom Perl script. Finally, we used the Comparing Partitions on-line tool (http://darwin.phyloviz.net/ComparingPartitions/index.php?link=Tool) to calculate Simpson’s index of diversity [33, 35] and Adjusted Wallace [34] with 95 % confidence intervals [36].

### Whole-chromosome phylogenetic analysis

We assembled the 4 largest short-read sequence datasets generated during the 12 *L. monocytogenes* strain 08–5578 sequencing runs described above (SRR1373534, SRR1508282, SRR1507228, and SRR1373535). The datasets were assembled *de-novo* with SPAdes v3.0.0. Contiguous sequences were aligned with Mauve v2.3.1 and 3,138,152 nucleotide positions were phylogenetically analyzed with RAxML v8.1.1 [58] (GTRCATI + 25γ) for 100 bootstrap replicates.

### Availability of supporting data

Genome sequence data supporting the results of this article are available in the National Center for Biotechnology Information Sequence Read Archive (http://www.ncbi.nlm.nih.gov/sra) under accession numbers SRR1342176, SRR1342220, SRR1373524, SRR1373525, SRR1373527, SRR1373529, SRR1373530, SRR1373531, SRR1373534, SRR1373535, SRR1507228, and SRR1508282.

## Additional files

Additional file 1:
**Phylogenetic analysis of 68 aligned** ***Listeria monocytogenes*** **genomes.** Evolutionary relationships of 68 *L. monocytogenes* strains, including 29 that were used to calculate pan- and core-genomes during this study. (PDF 121 kb) Additional file 2:
**Sources and general features of closed chromosomes compared for calculation of pan- and core-genomes.** Serotypes, NCBI accession numbers, sources, years collected, countries of origin, chromosome length, GC content, numbers of proteins, and references for 29 *L. monocytogenes*. (PDF 97 kb) Additional file 3:
**Pan- and core-genome sizes of 29 closed** ***Listeria monocytogenes*** **chromosome sequences.** Estimated pan- and core-genome sizes calculated from 2–29 randomly selected chromosome sequences. (PDF 251 kb) Additional file 4:
**List of 1013 open-reading frames that comprise the high-confidence core genome with NCBI numbers for** ***Listeria monocytogenes*** **strain 08–5578.** (PDF 403 kb) Additional file 5:
**Comparisons of the frequencies of proteins within Clusters of Orthologous Groups categories.** Frequencies of different functional classes of proteins encoded by open-reading frames from a complete *Listeria monocytogenes* chromosome (08–5578) and the high-confidence core. (PDF 117 kb) Additional file 6:
**Distribution of 1013 genes encoding ORFs present in the calculated high-confidence core-genome of** ***L. monocytogenes*** **strain 08–5578.** ORFs mapped onto a chromosome map. (PDF 389 kb) Additional file 7:
**Diagram illustrating tasks performed by the** ***Listeria monocytogenes*** **Core-Genome Sequence Typer.** (PDF 117 kb) Additional file 8:
***Listeria monocytogenes*** **high-confidence core profiles grouped by nucleotide distances.** Proposed nomenclature in which *Listeria monocytogenes* high-confidence core profiles are grouped by nucleotide distances. (PDF 144 kb) Additional file 9:
**Phylogenetic analysis of 115 high-confidence core profiles.** Phylogenetic relationships of 114 HCC profiles that comprise the CGST database and a single randomly selected test subject. (PDF 112 kb) Additional file 10:
**Comparison of 12 sets of Illumina MiSeq data of varying qualities.** Numbers of: a) SNPs, b) missing ORFs, and c) partial ORFs observed when genome sequence datasets of low to high quality are analyzed using the LmCGST pipeline. (PDF 112 kb) Additional file 11:
**Summary statistics describing data obtained from Illumina sequencing runs of** ***Listeria monocytogenes*** **strain 08–5578 genomic DNA samples.** (PDF 97 kb) Additional file 12:
**Processing times for steps of the LmCGST pipeline with different database sizes.** (PDF 86 kb) Additional file 13:
**Predicted LmCGST processing times with increasing database sizes.** Amounts of time predicted to be required for the assembly, annotation, HCC comparison, and tree-building steps with database sizes from 25 to 500 HCC profiles. (PDF 108 kb) Additional file 14:
**Simpson’s index of diversity and 95 % confidence intervals.** Measurements of the probabilities that two strains sampled randomly from a population will be assigned to two different types with the CGST, PFGE, ribotyping, and MLST methods. (PDF 85 kb) Additional file 15:
**Diagram of adjusted Wallace coefficients calculated from pulsed-field gel electrophoresis (PFGE), ribotyping, and core-genome sequence typing (CGST) data.** (PDF 124 kb) Additional file 16:
**Phylogenetic analysis of** ***de-novo*** **assemblies calculated from short-read sequence data obtained from four sequencing runs of a single** ***Listeria monocytogenes*** **strain 08–5578 DNA extraction.** (PDF 117 kb) Additional file 17:
**Distance analysis of** ***de-novo*** **assemblies calculated from short-read sequence data obtained from four sequencing runs of a single** ***Listeria monocytogenes*** **strain 08–5578 DNA extraction.** (PDF 96 kb) Additional file 18:
**Data used to calculate unique** ***Listeria monocytogenes*** **high-confidence core genomes.** (PDF 109 kb)

## Abbreviations

MLST: Multi-locus sequence typing
SNP: Single-nucleotide polymorphism
WGS: Whole-genome sequence
wgMLST: Whole-genome multi-locus sequence typing
cgMLST: Core-genome multi-locus sequence typing
CGST: Core-genome sequence typing
HCC: High-confidence core
ORF: Open-reading frame
NCBI: National center for biotechnology information
BLAST: Basic local alignment search tool
