# Supplementary material for: The Listeria monocytogenes Core-Genome Sequence Typer (LmCGST): a bioinformatic pipeline for molecular characterization with next-generation sequence data
Source: BMC Microbiol. 2015 Oct 22;15:224. doi: 10.1186/s12866-015-0526-1 (PMC4618880; doi:10.1186/s12866-015-0526-1)

Supporting Information for:

The *Listeria monocytogenes* Core-Genome Sequence Typer (LmCGST): a bioinformatic pipeline for molecular characterization with next-generation sequence data

Arthur W. Pightling[1], Nicholas Petronella[2], Franco Pagotto[1*]

[1] Listeriosis Reference Service for Canada, Microbiology Research Division, Bureau of Microbial Hazards, Food Directorate, Health Products and Food Branch, Health Canada, 251 Sir Frederick Banting Driveway, Ottawa, Ontario, K1A 0K9 Canada

[2] Biostatistics and Modelling Division, Bureau of Food Surveillance and Science Integration, Food Directorate, Health Products and Food Branch, Health Canada, 251 Sir Frederick Banting Driveway, Ottawa, Ontario, K1A 0K9 Canada

[*] Corresponding author

E-mails:

Franco Pagotto: Franco.Pagotto@hc-sc.gc.ca
Arthur Pightling: Arthur.Pightling@hc-sc.gc.ca
Nicholas Petronella: Nicholas.Petronella@hc-sc.gc.ca

Additional file 1: Phylogenetic analysis of 68 aligned *Listeria monocytogenes* genomes. Trees were calculated from 2,963,005 aligned nucleotides with the Randomized Axelerated Maximum Likelihood tool (GTRCATI+25γ). The best of 100 bootstrap replicates is shown. Red labels indicate closed high-quality genome sequences used for calculation of the *L. monocytogenes* core-genome.

Additional file 1

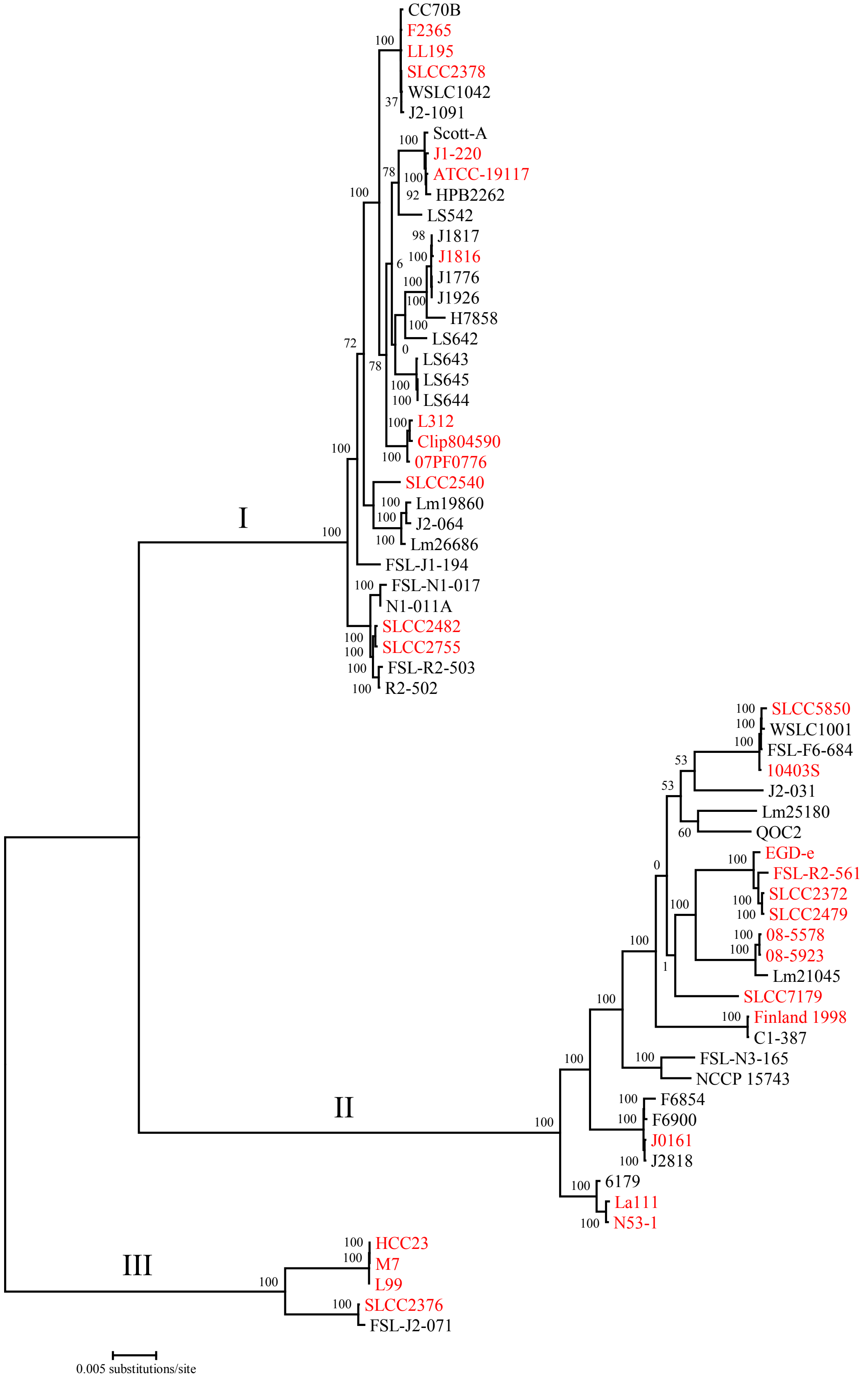

Supplement: Additional file 1: — Phylogenetic analysis of 68 aligned Listeria monocytogenes genomes. Evolutionary relationships of 68 L. monocytogenes strains, including 29 that were used to calculate pan- and core-genomes during this study. (PDF 121 kb) [file 12866_2015_526_MOESM1_ESM.pdf]
