# Supplementary material for: The Listeria monocytogenes Core-Genome Sequence Typer (LmCGST): a bioinformatic pipeline for molecular characterization with next-generation sequence data
Source: BMC Microbiol. 2015 Oct 22;15:224. doi: 10.1186/s12866-015-0526-1 (PMC4618880; doi:10.1186/s12866-015-0526-1)
Supplement: Additional file 2: — Sources and general features of closed chromosomes compared for calculation of pan- and core-genomes. Serotypes, NCBI accession numbers, sources, years collected, countries of origin, chromosome length, GC content, numbers of proteins, and references for 29 L. monocytogenes. (PDF 97 kb) [file 12866_2015_526_MOESM2_ESM.pdf]

Supporting Information for:

The *Listeria monocytogenes* Core-Genome Sequence Typer (LmCGST): a bioinformatic pipeline for molecular characterization with next-generation sequence data

Arthur W. Pightling[1], Nicholas Petronella[2], Franco Pagotto[1*]

[1] Listeriosis Reference Service for Canada, Microbiology Research Division, Bureau of Microbial Hazards, Food Directorate, Health Products and Food Branch, Health Canada, 251 Sir Frederick Banting Driveway, Ottawa, Ontario, K1A 0K9 Canada

[2] Biostatistics and Modelling Division, Bureau of Food Surveillance and Science Integration, Food Directorate, Health Products and Food Branch, Health Canada, 251 Sir Frederick Banting Driveway, Ottawa, Ontario, K1A 0K9 Canada

[*] Corresponding author

E-mails:

Franco Pagotto: Franco.Pagotto@hc-sc.gc.ca
Arthur Pightling: Arthur.Pightling@hc-sc.gc.ca
Nicholas Petronella: Nicholas.Petronella@hc-sc.gc.ca

Additional file 2: Sources and general features of closed chromosomes compared for calculation of pan- and core-genomes.

Strain	Sero-type	Accession number	Source	Year	Country	Length (Mb)	GC	Protein	Ref
08-5578	1/2a	NC_013766.2	Clinical	2008	Canada	3.03	38.0	3010	[12]
08-5923	1/2a	NC_013768.1	Clinical	2008	Canada	3.00	38.0	2966	[12]
10403S	1/2a	NC_017544.1	Clinical			2.90	38.0	2814	[47]
EGD-e	1/2a	NC_022568.1	Rabbit	1926	UK	2.94	38.0	2848	[48]
J0161	1/2a	NC_017545.1	Clinical	2000	USA	3.00		2955	[49]
La111	1/2a	NC_020557.1	Salmon	1996	Denmark	2.78		3131	[50]
N53-1	1/2a	NC_020558.1	Environment	2002	Denmark	2.78		3150	[50]
SLCC5850	1/2a	NC_018592.1	Rabbit	1924	UK	2.91	38.0	2865	[24]
SLCC2755	1/2b	NC_018587.1	Chinchilla	1967		2.97	38.1	2877	[24]
FSL-R2-561	1/2c	NC_017546.1				2.97		2910	
SLCC2372	1/2c	NC_018588.1	Clinical	1935	UK	2.97	38.0	2990	[24]
Finland 1998	3a	NC_017547.1		1998	Finland	2.87		2762	
SLCC7179	3a	NC_018593.1	Cheese	1986	Austria	2.88	38.0	2826	[24]
SLCC2540	3b	NC_018586.1	Clinical	1956	USA	2.98	37.9	2907	[24]
SLCC2479	3c	NC_018589.1		1966		2.97	38.0	2935	[24]
HCC23	4a	NC_011660.1	Catfish			2.98		2974	[51]
L99	4a	NC_017529.1	Cheese	1950	Netherlands	2.98	38.2	2925	[52]
M7	4a	NC_017537.1	Milk		China	2.98		2977	[53]
07PF0776	4b	NC_017728.1	Clinical			2.90		2797	[54]
ATCC 19117	4b	NC_018584.1	Sheep			2.95	38.0	2868	[24]
Clip814590	4b	NC_012488.1				2.91		2766	[52]
F2365	4b	NC_002973.6	Cheese	1985	USA	2.91	38.0	2821	[55]
J1816	4b	NC_021829.1		2002	USA	2.95		2794	[56]
J1-220	4b	NC_021830.1		2002	USA	3.03		2985	[56]
L312	4b	NC_018642.1	Cheese			2.91		2821	[24]
LL195	4b	NC_019556.1	Clinical	1983-87	Switzerland	2.90	38.0	2838	[57]
SLCC2376	4c	NC_018590.1	Poultry			2.84	38.3	2755	[24]
SLCC2378	4e	NC_018585.1	Poultry			2.94	38.0	2874	[24]
SLCC2482	7	NC_018591.1	Clinical	1966		2.94	38.0	2874	[24]
