# Supplementary material for: The Listeria monocytogenes Core-Genome Sequence Typer (LmCGST): a bioinformatic pipeline for molecular characterization with next-generation sequence data
Source: BMC Microbiol. 2015 Oct 22;15:224. doi: 10.1186/s12866-015-0526-1 (PMC4618880; doi:10.1186/s12866-015-0526-1)

Supporting Information for:

The *Listeria monocytogenes* Core-Genome Sequence Typer (LmCGST): a bioinformatic pipeline for molecular characterization with next-generation sequence data

Arthur W. Pightling[1], Nicholas Petronella[2], Franco Pagotto[1*]

[1] Listeriosis Reference Service for Canada, Microbiology Research Division, Bureau of Microbial Hazards, Food Directorate, Health Products and Food Branch, Health Canada, 251 Sir Frederick Banting Driveway, Ottawa, Ontario, K1A 0K9 Canada

[2] Biostatistics and Modelling Division, Bureau of Food Surveillance and Science Integration, Food Directorate, Health Products and Food Branch, Health Canada, 251 Sir Frederick Banting Driveway, Ottawa, Ontario, K1A 0K9 Canada

[*] Corresponding author

E-mails:

Franco Pagotto: Franco.Pagotto@hc-sc.gc.ca
Arthur Pightling: Arthur.Pightling@hc-sc.gc.ca
Nicholas Petronella: Nicholas.Petronella@hc-sc.gc.ca

Additional file 3: Pan- and core-genome sizes of 29 closed *Listeria monocytogenes* chromosome sequences. The numbers of translated open reading frames (ORFs) in the pan- and core-genomes were calculated by identifying homologs with a 60% sequence identity and 80% coverage. Chromosomes were added 1000 times with replacement in a randomized order. The total numbers of ORFs (pan-genome; open circles) and the numbers of mutually conserved ORFs (core-genome; closed circles) was noted. Grey circles indicate mean numbers of ORFs. Each diamond indicates the numbers of ORFs calculated by Kuenne *et al*. [24].

Additional file 3

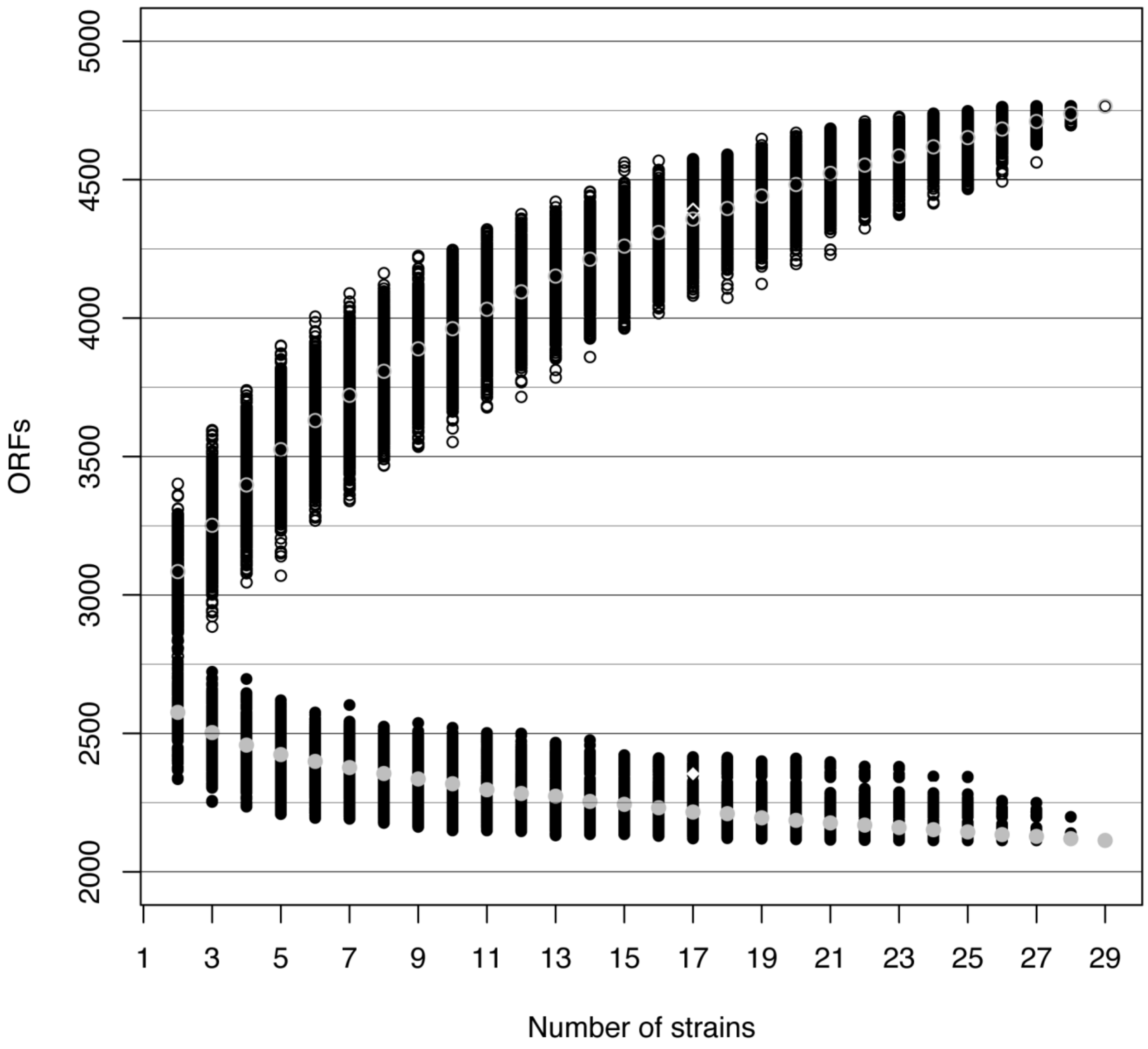

Supplement: Additional file 3: — Pan- and core-genome sizes of 29 closed Listeria monocytogenes chromosome sequences. Estimated pan- and core-genome sizes calculated from 2–29 randomly selected chromosome sequences. (PDF 251 kb) [file 12866_2015_526_MOESM3_ESM.pdf]
