# Supplementary material for: The Listeria monocytogenes Core-Genome Sequence Typer (LmCGST): a bioinformatic pipeline for molecular characterization with next-generation sequence data
Source: BMC Microbiol. 2015 Oct 22;15:224. doi: 10.1186/s12866-015-0526-1 (PMC4618880; doi:10.1186/s12866-015-0526-1)
Supplement: Additional file 4: — List of 1013 open-reading frames that comprise the high-confidence core genome with NCBI numbers for Listeria monocytogenes strain 08–5578. (PDF 403 kb) [file 12866_2015_526_MOESM4_ESM.pdf]

Supporting Information for:

The *Listeria monocytogenes* Core-Genome Sequence Typer (LmCGST): a bioinformatic pipeline for molecular characterization with next-generation sequence data

Arthur W. Pightling[1], Nicholas Petronella[2], Franco Pagotto[1*]

[1] Listeriosis Reference Service for Canada, Microbiology Research Division, Bureau of Microbial Hazards, Food Directorate, Health Products and Food Branch, Health Canada, 251 Sir Frederick Banting Driveway, Ottawa, Ontario, K1A 0K9 Canada

[2] Biostatistics and Modelling Division, Bureau of Food Surveillance and Science Integration, Food Directorate, Health Products and Food Branch, Health Canada, 251 Sir Frederick Banting Driveway, Ottawa, Ontario, K1A 0K9 Canada

[*] Corresponding author

E-mails:

Franco Pagotto: Franco.Pagotto@hc-sc.gc.ca
Arthur Pightling: Arthur.Pightling@hc-sc.gc.ca
Nicholas Petronella: Nicholas.Petronella@hc-sc.gc.ca

Additional file 4: List of 1013 open-reading frames that comprise the high-confidence core genome with NCBI numbers for *Listeria monocytogenes* strain 08-5578.

Protein name	Protein sequence	Gene sequence
hypothetical protein LM5578 0004	gi|284800259|ref|YP 003412124.1	gi|662858600|ref|NC_013766.2|:1450-2313
hypothetical protein LM5578 0005	gi|284800260|ref|YP 003412125.1	gi|662858600|ref|NC_013766.2|:2310-2930
hypothetical protein LM5578 0008	gi|284800263|ref|YP 003412128.1	gi|662858600|ref|NC_013766.2|:4599-5867
L-rhamnose isomerase	gi|284800265|ref|YP 003412130.1	gi|662858600|ref|NC_013766.2|:7350-8612
rhamnulose-1-phosphate aldolase	gi|284800266|ref|YP 003412131.1	gi|662858600|ref|NC_013766.2|:8625-9446
hypothetical protein LM5578 0013	gi|284800268|ref|YP 003412133.1	gi|662858600|ref|NC_013766.2|:c11226-9814
hypothetical protein LM5578 0018	gi|284800273|ref|YP 003412138.1	gi|662858600|ref|NC_013766.2|:17136-18428
hypothetical protein LM5578 0019	gi|284800274|ref|YP 003412139.1	gi|662858600|ref|NC_013766.2|:18503-19384
hypothetical protein LM5578 0020	gi|284800275|ref|YP 003412140.1	gi|662858600|ref|NC_013766.2|:19371-20222
hypothetical protein LM5578 0022	gi|284800277|ref|YP 003412142.1	gi|662858600|ref|NC_013766.2|:21306-22100
hypothetical protein LM5578 0026	gi|284800281|ref|YP 003412146.1	gi|662858600|ref|NC_013766.2|:26478-27140
hypothetical protein LM5578 0028	gi|284800283|ref|YP 003412148.1	gi|662858600|ref|NC_013766.2|:c28238-27639
hypothetical protein LM5578 0033	gi|284800288|ref|YP 003412153.1	gi|662858600|ref|NC_013766.2|:31927-33114

amino-terminal domain-containing protein	gi|284800292|ref|YP 003412157.1	gi|662858600|ref|NC_013766.2|:37589-39931
hypothetical protein LM5578 0039	gi|284800294|ref|YP 003412159.1	gi|662858600|ref|NC_013766.2|:41329-42729
hypothetical protein LM5578 0041	gi|284800296|ref|YP 003412161.1	gi|662858600|ref|NC_013766.2|:44023-45216
glucose-inhibited division protein B	gi|284800312|ref|YP 003412177.1	gi|662858600|ref|NC_013766.2|:56888-57604
hypothetical protein LM5578 0063	gi|284800318|ref|YP 003412183.1	gi|662858600|ref|NC_013766.2|:62165-63070
hypothetical protein LM5578 0064	gi|284800319|ref|YP 003412184.1	gi|662858600|ref|NC_013766.2|:63054-63860
hypothetical protein LM5578 0065	gi|284800320|ref|YP 003412185.1	gi|662858600|ref|NC_013766.2|:64032-64886
hypothetical protein LM5578 0067	gi|284800322|ref|YP 003412187.1	gi|662858600|ref|NC_013766.2|:65392-66114
partition protein, ParA-like protein	gi|284800323|ref|YP 003412188.1	gi|662858600|ref|NC_013766.2|:66338-67099
partition protein ParB homolg	gi|284800324|ref|YP 003412189.1	gi|662858600|ref|NC_013766.2|:67092-67943
catalase	gi|284800329|ref|YP 003412194.1	gi|662858600|ref|NC_013766.2|:72319-73785
hypothetical protein LM5578 0075	gi|284800330|ref|YP 003412195.1	gi|662858600|ref|NC_013766.2|:73996-75912
hypothetical protein LM5578 0076	gi|284800331|ref|YP 003412196.1	gi|662858600|ref|NC_013766.2|:76062-77357
bifunctional glutamate--cysteine ligase/glutathione synthetase	gi|284800343|ref|YP 003412208.1	gi|662858600|ref|NC_013766.2|:c91313-88983
hypothetical protein LM5578 0095	gi|284800350|ref|YP 003412215.1	gi|662858600|ref|NC_013766.2|:c97683-96331

DNA topoisomerase III	gi|284800358|ref|YP 003412223.1	gi|662858600|ref|NC_013766.2|:105943-108096
hypothetical protein LM5578 0104	gi|284800359|ref|YP 003412224.1	gi|662858600|ref|NC_013766.2|:108205-109872
hypothetical protein LM5578 0105	gi|284800360|ref|YP 003412225.1	gi|662858600|ref|NC_013766.2|:110051-111388
hypothetical protein LM5578 0108	gi|284800363|ref|YP 003412228.1	gi|662858600|ref|NC_013766.2|:c115415-113694
hypothetical protein LM5578 0114	gi|284800369|ref|YP 003412234.1	gi|662858600|ref|NC_013766.2|:c122070-120343
hypothetical protein LM5578 0115	gi|284800370|ref|YP 003412235.1	gi|662858600|ref|NC_013766.2|:122400-123089
hypothetical protein LM5578 0124	gi|284800379|ref|YP 003412244.1	gi|662858600|ref|NC_013766.2|:c131821-130139
hypothetical protein LM5578 0127	gi|284800382|ref|YP 003412247.1	gi|662858600|ref|NC_013766.2|:136656-137408
hypothetical protein LM5578 0138	gi|284800392|ref|YP 003412257.1	gi|662858600|ref|NC_013766.2|:c144093-143359
putative 6-phosphogluconolactonase	gi|284800393|ref|YP 003412258.1	gi|662858600|ref|NC_013766.2|:144173-144892
hypothetical protein LM5578 0143	gi|284800397|ref|YP 003412262.1	gi|662858600|ref|NC_013766.2|:148951-149964
hypothetical protein LM5578 0145	gi|284800399|ref|YP 003412264.1	gi|662858600|ref|NC_013766.2|:151685-153427
pepdidoglycan bound protein	gi|284800400|ref|YP 003412265.1	gi|662858600|ref|NC_013766.2|:153568-154515
recombination protein RecR	gi|284800412|ref|YP 003412277.1	gi|662858600|ref|NC_013766.2|:164508-165104
hypothetical protein LM5578 0160	gi|284800414|ref|YP 003412279.1	gi|662858600|ref|NC_013766.2|:c166252-165410

hypothetical protein LM5578 0161	gi|284800415|ref|YP 003412280.1	gi|662858600|ref|NC_013766.2|:c167212-166373
hypothetical protein LM5578 0166	gi|284800420|ref|YP 003412285.1	gi|662858600|ref|NC_013766.2|:170457-171836
hypothetical protein LM5578 0167	gi|284800421|ref|YP 003412286.1	gi|662858600|ref|NC_013766.2|:171889-172515
hypothetical protein LM5578 0172	gi|284800426|ref|YP 003412291.1	gi|662858600|ref|NC_013766.2|:178815-179945
hypothetical protein LM5578 0176	gi|284800430|ref|YP 003412295.1	gi|662858600|ref|NC_013766.2|:c183850-182543
potassium-transporting ATPase subunit A	gi|284800432|ref|YP 003412297.1	gi|662858600|ref|NC_013766.2|:184632-186317
potassium-transporting atpase c chain	gi|284800434|ref|YP 003412299.1	gi|662858600|ref|NC_013766.2|:188389-188961
hypothetical protein LM5578 0181	gi|284800435|ref|YP 003412300.1	gi|662858600|ref|NC_013766.2|:188977-191667
hypothetical protein LM5578 0182	gi|284800436|ref|YP 003412301.1	gi|662858600|ref|NC_013766.2|:191664-192359
hypothetical protein LM5578 0183	gi|284800437|ref|YP 003412302.1	gi|662858600|ref|NC_013766.2|:192400-193212
hypothetical protein LM5578 0192	gi|284800446|ref|YP 003412311.1	gi|662858600|ref|NC_013766.2|:198940-201009
hypothetical protein LM5578 0195	gi|284800449|ref|YP 003412314.1	gi|662858600|ref|NC_013766.2|:201908-203179
hypothetical protein LM5578 0196	gi|284800450|ref|YP 003412315.1	gi|662858600|ref|NC_013766.2|:203216-204268
hypothetical protein LM5578 0197	gi|284800451|ref|YP 003412316.1	gi|662858600|ref|NC_013766.2|:204270-205301
hypothetical protein LM5578 0201	gi|284800455|ref|YP 003412320.1	gi|662858600|ref|NC_013766.2|:208665-209309

elongation factor G	gi|284800460|ref|YP 003412325.1	gi|662858600|ref|NC_013766.2|:212507-214594
elongation factor Tu	gi|284800461|ref|YP 003412326.1	gi|662858600|ref|NC_013766.2|:214703-215890
hypothetical protein LM5578 0212	gi|284800466|ref|YP 003412331.1	gi|662858600|ref|NC_013766.2|:220409-221401
hypothetical protein LM5578 0213	gi|284800467|ref|YP 003412332.1	gi|662858600|ref|NC_013766.2|:221414-222163
hypothetical protein LM5578 0214	gi|284800468|ref|YP 003412333.1	gi|662858600|ref|NC_013766.2|:222123-222842
hypothetical protein LM5578 0217	gi|284800471|ref|YP 003412336.1	gi|662858600|ref|NC_013766.2|:224505-225491
hypothetical protein LM5578 0219	gi|284800473|ref|YP 003412338.1	gi|662858600|ref|NC_013766.2|:c227937-226957
hypothetical protein LM5578 0220	gi|284800474|ref|YP 003412339.1	gi|662858600|ref|NC_013766.2|:c228501-227959
hypothetical protein LM5578 0222	gi|284800476|ref|YP 003412341.1	gi|662858600|ref|NC_013766.2|:c231030-229144
hypothetical protein LM5578 0223	gi|284800477|ref|YP 003412342.1	gi|662858600|ref|NC_013766.2|:231478-232377
hypothetical protein LM5578 0224	gi|284800478|ref|YP 003412343.1	gi|662858600|ref|NC_013766.2|:232541-233623
hypothetical protein LM5578 0226	gi|284800480|ref|YP 003412345.1	gi|662858600|ref|NC_013766.2|:234684-235484
50S ribosomal protein L3	gi|284800482|ref|YP 003412347.1	gi|662858600|ref|NC_013766.2|:236188-236817
50S ribosomal protein L4	gi|284800483|ref|YP 003412348.1	gi|662858600|ref|NC_013766.2|:236843-237466
50S ribosomal protein L2	gi|284800485|ref|YP 003412350.1	gi|662858600|ref|NC_013766.2|:237791-238624

30S ribosomal protein S3	gi|284800488|ref|YP 003412353.1	gi|662858600|ref|NC_013766.2|:239371-240027
50S ribosomal protein L5	gi|284800494|ref|YP 003412359.1	gi|662858600|ref|NC_013766.2|:241760-242299
preprotein translocase subunit SecY	gi|284800502|ref|YP 003412367.1	gi|662858600|ref|NC_013766.2|:245127-246422
hypothetical protein LM5578 0249	gi|284800503|ref|YP 003412368.1	gi|662858600|ref|NC_013766.2|:246482-247129
DNA-directed RNA polymerase subunit alpha	gi|284800508|ref|YP 003412373.1	gi|662858600|ref|NC_013766.2|:248856-249800
hypothetical protein LM5578 0258	gi|284800512|ref|YP 003412377.1	gi|662858600|ref|NC_013766.2|:c253236-252574
cobalt transporter ATP-binding subunit	gi|284800513|ref|YP 003412378.1	gi|662858600|ref|NC_013766.2|:253367-254206
cobalt transporter ATP-binding subunit	gi|284800514|ref|YP 003412379.1	gi|662858600|ref|NC_013766.2|:254182-255048
tRNA pseudouridine synthase A	gi|284800516|ref|YP 003412381.1	gi|662858600|ref|NC_013766.2|:255854-256600
hypothetical protein LM5578 0267	gi|284800521|ref|YP 003412386.1	gi|662858600|ref|NC_013766.2|:259878-260729
ATP:guanido phosphotransferase	gi|284800526|ref|YP 003412391.1	gi|662858600|ref|NC_013766.2|:270180-271202
endopeptidase Clp ATP-binding chain C	gi|284800527|ref|YP 003412392.1	gi|662858600|ref|NC_013766.2|:271231-273693
DNA repair protein RadA	gi|284800528|ref|YP 003412393.1	gi|662858600|ref|NC_013766.2|:273839-275212
2-C-methyl-D-erythritol 4-phosphate cytidylyltransferase	gi|284800530|ref|YP 003412395.1	gi|662858600|ref|NC_013766.2|:276439-277137
RNA polymerase factor sigma-70	gi|284800538|ref|YP 003412403.1	gi|662858600|ref|NC_013766.2|:283275-283880

50S ribosomal protein L1	gi|284800543|ref|YP 003412408.1	gi|662858600|ref|NC_013766.2|:286112-286801
hypothetical protein LM5578 0305	gi|284800558|ref|YP 003412423.1	gi|662858600|ref|NC_013766.2|:306990-308450
succinyl-diaminopimelate desuccinylase	gi|284800563|ref|YP 003412428.1	gi|662858600|ref|NC_013766.2|:315735-316874
hypothetical protein LM5578 0312	gi|284800565|ref|YP 003412430.1	gi|662858600|ref|NC_013766.2|:317445-318404
hypothetical protein LM5578 0317	gi|284800570|ref|YP 003412435.1	gi|662858600|ref|NC_013766.2|:c324097-323285
hypothetical protein LM5578 0320	gi|284800573|ref|YP 003412438.1	gi|662858600|ref|NC_013766.2|:c326536-325694
hypothetical protein LM5578 0321	gi|284800574|ref|YP 003412439.1	gi|662858600|ref|NC_013766.2|:c327374-326553
hypothetical protein LM5578 0323	gi|284800576|ref|YP 003412441.1	gi|662858600|ref|NC_013766.2|:c329601-328501
hypothetical protein LM5578 0327	gi|284800580|ref|YP 003412445.1	gi|662858600|ref|NC_013766.2|:c334456-333677
putative lipoprotein	gi|284800584|ref|YP 003412449.1	gi|662858600|ref|NC_013766.2|:c337057-336236
hypothetical protein LM5578 0333	gi|284800586|ref|YP 003412451.1	gi|662858600|ref|NC_013766.2|:338833-339546
hypothetical protein LM5578 0335	gi|284800588|ref|YP 003412453.1	gi|662858600|ref|NC_013766.2|:341560-342882
hypothetical protein LM5578 0336	gi|284800589|ref|YP 003412454.1	gi|662858600|ref|NC_013766.2|:342885-343724
hypothetical protein LM5578 0337	gi|284800590|ref|YP 003412455.1	gi|662858600|ref|NC_013766.2|:343844-344674
hypothetical protein LM5578 0344	gi|284800597|ref|YP 003412462.1	gi|662858600|ref|NC_013766.2|:c353316-352003

hypothetical protein LM5578 0360	gi|284800613|ref|YP 003412478.1	gi|662858600|ref|NC_013766.2|:c366592-365939
hypothetical protein LM5578 0362	gi|284800615|ref|YP 003412480.1	gi|662858600|ref|NC_013766.2|:367490-368392
short chain dehydrogenase	gi|284800632|ref|YP 003412497.1	gi|662858600|ref|NC_013766.2|:c390420-389656
hypothetical protein LM5578 0381	gi|284800634|ref|YP 003412499.1	gi|662858600|ref|NC_013766.2|:c391661-390897
hypothetical protein LM5578 0382	gi|284800635|ref|YP 003412500.1	gi|662858600|ref|NC_013766.2|:c392315-391665
hypothetical protein LM5578 0383	gi|284800636|ref|YP 003412501.1	gi|662858600|ref|NC_013766.2|:c393332-392337
hypothetical protein LM5578 0387	gi|284800640|ref|YP 003412505.1	gi|662858600|ref|NC_013766.2|:394787-395551
hypothetical protein LM5578 0389	gi|284800642|ref|YP 003412507.1	gi|662858600|ref|NC_013766.2|:c397602-396076
fumarate reductase flavoprotein subunit	gi|284800643|ref|YP 003412508.1	gi|662858600|ref|NC_013766.2|:397839-399359
hypothetical protein LM5578 0394	gi|284800647|ref|YP 003412512.1	gi|662858600|ref|NC_013766.2|:402602-403462
hypothetical protein LM5578 0395	gi|284800648|ref|YP 003412513.1	gi|662858600|ref|NC_013766.2|:403519-404289
hypothetical protein LM5578 0405	gi|284800658|ref|YP 003412523.1	gi|662858600|ref|NC_013766.2|:c412299-411586
hypothetical protein LM5578 0418	gi|284800671|ref|YP 003412536.1	gi|662858600|ref|NC_013766.2|:422365-423186
low temperature requirement protein A	gi|284800676|ref|YP 003412541.1	gi|662858600|ref|NC_013766.2|:c428215-427100
uracil-DNA glycosylase	gi|284800677|ref|YP 003412542.1	gi|662858600|ref|NC_013766.2|:428301-429011

hypothetical protein LM5578 0426	gi|284800679|ref|YP 003412544.1	gi|662858600|ref|NC_013766.2|:429475-430419
pyrroline-5-carboxylate reductase	gi|284800683|ref|YP 003412548.1	gi|662858600|ref|NC_013766.2|:c433014-432214
hypothetical protein LM5578 0447	gi|284800700|ref|YP 003412565.1	gi|662858600|ref|NC_013766.2|:450630-451439
hypothetical protein LM5578 0448	gi|284800701|ref|YP 003412566.1	gi|662858600|ref|NC_013766.2|:451733-453133
hypothetical protein LM5578 0461	gi|284800714|ref|YP 003412579.1	gi|662858600|ref|NC_013766.2|:462054-463160
hypothetical protein LM5578 0465	gi|284800718|ref|YP 003412583.1	gi|662858600|ref|NC_013766.2|:467357-468103
hypothetical protein LM5578 0470	gi|284800723|ref|YP 003412588.1	gi|662858600|ref|NC_013766.2|:473832-474677
hypothetical protein LM5578 0483	gi|284800736|ref|YP 003412601.1	gi|662858600|ref|NC_013766.2|:490480-491205
hypothetical protein LM5578 0484	gi|284800737|ref|YP 003412602.1	gi|662858600|ref|NC_013766.2|:c491913-491248
hypothetical protein LM5578 0485	gi|284800738|ref|YP 003412603.1	gi|662858600|ref|NC_013766.2|:c492689-491934
hypothetical protein LM5578 0487	gi|284800740|ref|YP 003412605.1	gi|662858600|ref|NC_013766.2|:c496111-494963
hypothetical protein LM5578 0488	gi|284800741|ref|YP 003412606.1	gi|662858600|ref|NC_013766.2|:c497063-496116
hypothetical protein LM5578 0490	gi|284800743|ref|YP 003412608.1	gi|662858600|ref|NC_013766.2|:498948-500231
hypothetical protein LM5578 0491	gi|284800744|ref|YP 003412609.1	gi|662858600|ref|NC_013766.2|:500232-501332
hypothetical protein LM5578 0508	gi|284800760|ref|YP 003412625.1	gi|662858600|ref|NC_013766.2|:515774-516349

hypothetical protein LM5578 0517	gi|284800769|ref|YP 003412634.1	gi|662858600|ref|NC_013766.2|:525328-525921
hypothetical protein LM5578 0518	gi|284800770|ref|YP 003412635.1	gi|662858600|ref|NC_013766.2|:526107-527066
hypothetical protein LM5578 0522	gi|284800774|ref|YP 003412639.1	gi|662858600|ref|NC_013766.2|:529566-530234
hypothetical protein LM5578 0524	gi|284800776|ref|YP 003412641.1	gi|662858600|ref|NC_013766.2|:531000-533060
hypothetical protein LM5578 0525	gi|284800777|ref|YP 003412642.1	gi|662858600|ref|NC_013766.2|:533064-533666
hypothetical protein LM5578 0528	gi|284800780|ref|YP 003412645.1	gi|662858600|ref|NC_013766.2|:534587-535237
hypothetical protein LM5578 0531	gi|284800783|ref|YP 003412648.1	gi|662858600|ref|NC_013766.2|:536604-537875
phosphoribosyl pyrophosphate synthetase	gi|284800784|ref|YP 003412649.1	gi|662858600|ref|NC_013766.2|:538015-538950
hypothetical protein LM5578 0539	gi|284800791|ref|YP 003412656.1	gi|662858600|ref|NC_013766.2|:c546497-545067
hypothetical protein LM5578 0540	gi|284800792|ref|YP 003412657.1	gi|662858600|ref|NC_013766.2|:c547433-546618
hypothetical protein LM5578 0543	gi|284800795|ref|YP 003412660.1	gi|662858600|ref|NC_013766.2|:549773-550780
hypothetical protein LM5578 0552	gi|284800804|ref|YP 003412669.1	gi|662858600|ref|NC_013766.2|:c563736-562075
hypothetical protein LM5578 0554	gi|284800806|ref|YP 003412671.1	gi|662858600|ref|NC_013766.2|:c566109-565369
transmembrane protein	gi|284800807|ref|YP 003412672.1	gi|662858600|ref|NC_013766.2|:566339-567814
hypothetical protein LM5578 0557	gi|284800809|ref|YP 003412674.1	gi|662858600|ref|NC_013766.2|:569314-570564

hypothetical protein LM5578 0559	gi|284800811|ref|YP 003412676.1	gi|662858600|ref|NC_013766.2|:572644-573498
hypothetical protein LM5578 0562	gi|284800814|ref|YP 003412679.1	gi|662858600|ref|NC_013766.2|:574754-576109
hypothetical protein LM5578 0563	gi|284800815|ref|YP 003412680.1	gi|662858600|ref|NC_013766.2|:c577117-576149
hypothetical protein LM5578 0568	gi|284800820|ref|YP 003412685.1	gi|662858600|ref|NC_013766.2|:582429-583622
hypothetical protein LM5578 0569	gi|284800821|ref|YP 003412686.1	gi|662858600|ref|NC_013766.2|:c584582-583662
hypothetical protein LM5578 0571	gi|284800823|ref|YP 003412688.1	gi|662858600|ref|NC_013766.2|:c586048-585062
hypothetical protein LM5578 0572	gi|284800824|ref|YP 003412689.1	gi|662858600|ref|NC_013766.2|:c586590-586069
hypothetical protein LM5578 0574	gi|284800826|ref|YP 003412691.1	gi|662858600|ref|NC_013766.2|:c588300-587050
hypothetical protein LM5578 0575	gi|284800827|ref|YP 003412692.1	gi|662858600|ref|NC_013766.2|:c589505-588558
hypothetical protein LM5578 0579	gi|284800831|ref|YP 003412696.1	gi|662858600|ref|NC_013766.2|:592949-593791
hypothetical protein LM5578 0581	gi|284800833|ref|YP 003412698.1	gi|662858600|ref|NC_013766.2|:c595590-594955
hypothetical protein LM5578 0582	gi|284800834|ref|YP 003412699.1	gi|662858600|ref|NC_013766.2|:595800-596981
hypothetical protein LM5578 0586	gi|284800838|ref|YP 003412703.1	gi|662858600|ref|NC_013766.2|:c601163-600123
hypothetical protein LM5578 0587	gi|284800839|ref|YP 003412704.1	gi|662858600|ref|NC_013766.2|:601554-602468
glutamate dehydrogenase	gi|284800840|ref|YP 003412705.1	gi|662858600|ref|NC_013766.2|:c603887-602511

ATP phosphoribosyltransferase regulatory subunit	gi|284800849|ref|YP 003412714.1	gi|662858600|ref|NC_013766.2|:c610763-609582
histidinol-phosphatase	gi|284800850|ref|YP 003412715.1	gi|662858600|ref|NC_013766.2|:610915-611742
hypothetical protein LM5578 0608	gi|284800860|ref|YP 003412725.1	gi|662858600|ref|NC_013766.2|:620510-621172
preprotein translocase subunit SecA	gi|284800863|ref|YP 003412728.1	gi|662858600|ref|NC_013766.2|:624342-626672
hypothetical protein LM5578 0612	gi|284800864|ref|YP 003412729.1	gi|662858600|ref|NC_013766.2|:626788-627960
hypothetical protein LM5578 0618	gi|284800870|ref|YP 003412735.1	gi|662858600|ref|NC_013766.2|:634575-636344
hypothetical protein LM5578 0619	gi|284800871|ref|YP 003412736.1	gi|662858600|ref|NC_013766.2|:636341-637114
hypothetical protein LM5578 0620	gi|284800872|ref|YP 003412737.1	gi|662858600|ref|NC_013766.2|:637242-637784
hypothetical protein LM5578 0621	gi|284800873|ref|YP 003412738.1	gi|662858600|ref|NC_013766.2|:638012-638812
homoserine O-acetyltransferase	gi|284800874|ref|YP 003412739.1	gi|662858600|ref|NC_013766.2|:c639957-638851
hypothetical protein LM5578 0623	gi|284800875|ref|YP 003412740.1	gi|662858600|ref|NC_013766.2|:c641251-639974
hypothetical protein LM5578 0625	gi|284800877|ref|YP 003412742.1	gi|662858600|ref|NC_013766.2|:c643016-642315
putative biotin biosynthesis protein BioY	gi|284800878|ref|YP 003412743.1	gi|662858600|ref|NC_013766.2|:c643640-643092
hypothetical protein LM5578 0628	gi|284800880|ref|YP 003412745.1	gi|662858600|ref|NC_013766.2|:644159-644749
hypothetical protein LM5578 0635	gi|284800887|ref|YP 003412752.1	gi|662858600|ref|NC_013766.2|:649400-651124

azoreductase	gi|284800891|ref|YP 003412756.1	gi|662858600|ref|NC_013766.2|:c655954-655328
hypothetical protein LM5578 0641	gi|284800893|ref|YP 003412758.1	gi|662858600|ref|NC_013766.2|:656575-657516
glycerophosphoryl diester phosphodiesterase	gi|284800896|ref|YP 003412761.1	gi|662858600|ref|NC_013766.2|:658491-660242
hypothetical protein LM5578 0653	gi|284800905|ref|YP 003412770.1	gi|662858600|ref|NC_013766.2|:c665705-665004
hypothetical protein LM5578 0654	gi|284800906|ref|YP 003412771.1	gi|662858600|ref|NC_013766.2|:c667470-665791
pepdidoglycan bound protein	gi|284800907|ref|YP 003412772.1	gi|662858600|ref|NC_013766.2|:667776-672524
hypothetical protein LM5578 0663	gi|284800915|ref|YP 003412780.1	gi|662858600|ref|NC_013766.2|:679097-679801
hypothetical protein LM5578 0714	gi|284800966|ref|YP 003412831.1	gi|662858600|ref|NC_013766.2|:717886-718803
hypothetical protein LM5578 0715	gi|284800967|ref|YP 003412832.1	gi|662858600|ref|NC_013766.2|:719069-720949
hypothetical protein LM5578 0718	gi|284800970|ref|YP 003412835.1	gi|662858600|ref|NC_013766.2|:721916-722566
hypothetical protein LM5578 0719	gi|284800971|ref|YP 003412836.1	gi|662858600|ref|NC_013766.2|:c724447-722606
hypothetical protein LM5578 0721	gi|284800973|ref|YP 003412838.1	gi|662858600|ref|NC_013766.2|:726142-726981
hypothetical protein LM5578 0723	gi|284800975|ref|YP 003412840.1	gi|662858600|ref|NC_013766.2|:727446-728396
hypothetical protein LM5578 0724	gi|284800976|ref|YP 003412841.1	gi|662858600|ref|NC_013766.2|:728420-729061
hypothetical protein LM5578 0726	gi|284800978|ref|YP 003412843.1	gi|662858600|ref|NC_013766.2|:731840-732487

hypothetical protein LM5578 0727	gi|284800979|ref|YP 003412844.1	gi|662858600|ref|NC_013766.2|:c733032-732496
hypothetical protein LM5578 0728	gi|284800980|ref|YP 003412845.1	gi|662858600|ref|NC_013766.2|:c733942-733019
hypothetical protein LM5578 0733	gi|284800985|ref|YP 003412850.1	gi|662858600|ref|NC_013766.2|:736309-736944
hypothetical protein LM5578 0734	gi|284800986|ref|YP 003412851.1	gi|662858600|ref|NC_013766.2|:c737830-736934
hypothetical protein LM5578 0740	gi|284800992|ref|YP 003412857.1	gi|662858600|ref|NC_013766.2|:740652-741215
flagellar biosynthesis protein FliR	gi|284801008|ref|YP 003412873.1	gi|662858600|ref|NC_013766.2|:750008-750769
flagellar biosynthesis protein FlhA	gi|284801010|ref|YP 003412875.1	gi|662858600|ref|NC_013766.2|:751878-753953
flagellar biosynthesis regulator FlhF	gi|284801011|ref|YP 003412876.1	gi|662858600|ref|NC_013766.2|:753975-755198
hypothetical protein LM5578 0762	gi|284801013|ref|YP 003412878.1	gi|662858600|ref|NC_013766.2|:756003-756791
flagellar motor protein MotA	gi|284801015|ref|YP 003412880.1	gi|662858600|ref|NC_013766.2|:757178-758029
hypothetical protein LM5578 0765	gi|284801016|ref|YP 003412881.1	gi|662858600|ref|NC_013766.2|:757989-758816
hypothetical protein LM5578 0767	gi|284801018|ref|YP 003412883.1	gi|662858600|ref|NC_013766.2|:759349-761262
flagellin	gi|284801020|ref|YP 003412885.1	gi|662858600|ref|NC_013766.2|:762421-763284
two-component sensor histidine kinase CheA	gi|284801022|ref|YP 003412887.1	gi|662858600|ref|NC_013766.2|:763938-765794
hypothetical protein LM5578 0774	gi|284801025|ref|YP 003412890.1	gi|662858600|ref|NC_013766.2|:766551-767597

hypothetical protein LM5578 0780	gi|284801031|ref|YP 003412896.1	gi|662858600|ref|NC_013766.2|:772095-773498
flagellar hook-associated protein FlgK	gi|284801035|ref|YP 003412900.1	gi|662858600|ref|NC_013766.2|:775716-777236
flagellar hook-associated protein FlgL	gi|284801036|ref|YP 003412901.1	gi|662858600|ref|NC_013766.2|:777248-778123
flagellar capping protein	gi|284801037|ref|YP 003412902.1	gi|662858600|ref|NC_013766.2|:778135-779424
flagellar MS-ring protein	gi|284801043|ref|YP 003412908.1	gi|662858600|ref|NC_013766.2|:781307-782959
flagellar assembly protein H	gi|284801045|ref|YP 003412910.1	gi|662858600|ref|NC_013766.2|:784055-784747
hypothetical protein LM5578 0796	gi|284801047|ref|YP 003412912.1	gi|662858600|ref|NC_013766.2|:786062-786730
hypothetical protein LM5578 0797	gi|284801048|ref|YP 003412913.1	gi|662858600|ref|NC_013766.2|:786744-787388
hypothetical protein LM5578 0803	gi|284801054|ref|YP 003412919.1	gi|662858600|ref|NC_013766.2|:792833-793561
hypothetical protein LM5578 0807	gi|284801058|ref|YP 003412923.1	gi|662858600|ref|NC_013766.2|:796181-796921
hypothetical protein LM5578 0822	gi|284801073|ref|YP 003412938.1	gi|662858600|ref|NC_013766.2|:809836-810525
hypothetical protein LM5578 0823	gi|284801074|ref|YP 003412939.1	gi|662858600|ref|NC_013766.2|:810522-811226
hypothetical protein LM5578 0825	gi|284801076|ref|YP 003412941.1	gi|662858600|ref|NC_013766.2|:813270-813773
hypothetical protein LM5578 0839	gi|284801090|ref|YP 003412955.1	gi|662858600|ref|NC_013766.2|:822311-823273
hypothetical protein LM5578 0840	gi|284801091|ref|YP 003412956.1	gi|662858600|ref|NC_013766.2|:823280-823888

hypothetical protein LM5578 0841	gi|284801092|ref|YP 003412957.1	gi|662858600|ref|NC_013766.2|:823898-824518
hypothetical protein LM5578 0842	gi|284801093|ref|YP 003412958.1	gi|662858600|ref|NC_013766.2|:824846-826102
hypothetical protein LM5578 0843	gi|284801094|ref|YP 003412959.1	gi|662858600|ref|NC_013766.2|:826168-827040
hypothetical protein LM5578 0844	gi|284801095|ref|YP 003412960.1	gi|662858600|ref|NC_013766.2|:827046-828035
hypothetical protein LM5578 0852	gi|284801103|ref|YP 003412968.1	gi|662858600|ref|NC_013766.2|:835666-836313
hypothetical protein LM5578 0853	gi|284801104|ref|YP 003412969.1	gi|662858600|ref|NC_013766.2|:c837342-836353
hypothetical protein LM5578 0854	gi|284801105|ref|YP 003412970.1	gi|662858600|ref|NC_013766.2|:837521-838450
hypothetical protein LM5578 0856	gi|284801107|ref|YP 003412972.1	gi|662858600|ref|NC_013766.2|:c839727-838861
mannose-specific PTS system protein IID	gi|284801112|ref|YP 003412977.1	gi|662858600|ref|NC_013766.2|:c843298-842420
hypothetical protein LM5578 0862	gi|284801113|ref|YP 003412978.1	gi|662858600|ref|NC_013766.2|:c844129-843317
hypothetical protein LM5578 0865	gi|284801116|ref|YP 003412981.1	gi|662858600|ref|NC_013766.2|:c848325-845509
hypothetical protein LM5578 0873	gi|284801124|ref|YP 003412989.1	gi|662858600|ref|NC_013766.2|:c859846-859145
hypothetical protein LM5578 0874	gi|284801125|ref|YP 003412990.1	gi|662858600|ref|NC_013766.2|:c860602-859961
hypothetical protein LM5578 0876	gi|284801127|ref|YP 003412992.1	gi|662858600|ref|NC_013766.2|:c862243-861713
hypothetical protein LM5578 0879	gi|284801130|ref|YP 003412995.1	gi|662858600|ref|NC_013766.2|:c865586-864825

hypothetical protein LM5578 0881	gi|284801132|ref|YP 003412997.1	gi|662858600|ref|NC_013766.2|:866244-866882
hypothetical protein LM5578 0891	gi|284801142|ref|YP 003413007.1	gi|662858600|ref|NC_013766.2|:875501-876043
hypothetical protein LM5578 0898	gi|284801149|ref|YP 003413014.1	gi|662858600|ref|NC_013766.2|:882575-883423
hypothetical protein LM5578 0899	gi|284801150|ref|YP 003413015.1	gi|662858600|ref|NC_013766.2|:883533-884090
hypothetical protein LM5578 0900	gi|284801151|ref|YP 003413016.1	gi|662858600|ref|NC_013766.2|:884107-884769
hypothetical protein LM5578 0902	gi|284801153|ref|YP 003413018.1	gi|662858600|ref|NC_013766.2|:885295-886119
hypothetical protein LM5578 0905	gi|284801156|ref|YP 003413021.1	gi|662858600|ref|NC_013766.2|:888685-890319
hypothetical protein LM5578 0909	gi|284801160|ref|YP 003413025.1	gi|662858600|ref|NC_013766.2|:c897582-895636
hypothetical protein LM5578 0910	gi|284801161|ref|YP 003413026.1	gi|662858600|ref|NC_013766.2|:c898623-897712
hypothetical protein LM5578 0918	gi|284801168|ref|YP 003413033.1	gi|662858600|ref|NC_013766.2|:c907773-906553
hypothetical protein LM5578 0924	gi|284801174|ref|YP 003413039.1	gi|662858600|ref|NC_013766.2|:918946-920049
hypothetical protein LM5578 0925	gi|284801175|ref|YP 003413040.1	gi|662858600|ref|NC_013766.2|:920154-921254
UDP-N-acetylmuramoylalanyl-D-glutamyl-2, 6- diamino pimelate-D-alanyl-D-alanyl ligase	gi|284801185|ref|YP 003413050.1	gi|662858600|ref|NC_013766.2|:929781-931193
hypothetical protein LM5578 0937	gi|284801187|ref|YP 003413052.1	gi|662858600|ref|NC_013766.2|:c933010-931985

hypothetical protein LM5578 0942	gi|284801192|ref|YP 003413057.1	gi|662858600|ref|NC_013766.2|:937819-938733
hypothetical protein LM5578 0944	gi|284801194|ref|YP 003413059.1	gi|662858600|ref|NC_013766.2|:939894-941492
hypothetical protein LM5578 0946	gi|284801196|ref|YP 003413061.1	gi|662858600|ref|NC_013766.2|:941938-943506
hypothetical protein LM5578 0953	gi|284801203|ref|YP 003413068.1	gi|662858600|ref|NC_013766.2|:948354-950372
hypothetical protein LM5578 0969	gi|284801219|ref|YP 003413084.1	gi|662858600|ref|NC_013766.2|:963092-963928
RNA polymerase sigma factor SigB	gi|284801225|ref|YP 003413090.1	gi|662858600|ref|NC_013766.2|:966653-967432
indirect negative regulation of sigma B dependant gene expression (serine phosphatase)	gi|284801226|ref|YP 003413091.1	gi|662858600|ref|NC_013766.2|:967433-968032
hypothetical protein LM5578 0977	gi|284801227|ref|YP 003413092.1	gi|662858600|ref|NC_013766.2|:968239-969864
hypothetical protein LM5578 0982	gi|284801232|ref|YP 003413097.1	gi|662858600|ref|NC_013766.2|:973722-975008
hypothetical protein LM5578 0983	gi|284801233|ref|YP 003413098.1	gi|662858600|ref|NC_013766.2|:975089-975802
hypothetical protein LM5578 0986	gi|284801236|ref|YP 003413101.1	gi|662858600|ref|NC_013766.2|:977598-978095
hypothetical protein LM5578 0989	gi|284801239|ref|YP 003413104.1	gi|662858600|ref|NC_013766.2|:c981362-980226
hypothetical protein LM5578 0993	gi|284801243|ref|YP 003413108.1	gi|662858600|ref|NC_013766.2|:983701-984513
hypothetical protein LM5578 0999	gi|284801249|ref|YP 003413114.1	gi|662858600|ref|NC_013766.2|:989825-991723

hypothetical protein LM5578 1001	gi|284801251|ref|YP 003413116.1	gi|662858600|ref|NC_013766.2|:993707-994747
hypothetical protein LM5578 1002	gi|284801252|ref|YP 003413117.1	gi|662858600|ref|NC_013766.2|:994766-995605
pantothenate kinase	gi|284801253|ref|YP 003413118.1	gi|662858600|ref|NC_013766.2|:995694-996614
hypothetical protein LM5578 1005	gi|284801255|ref|YP 003413120.1	gi|662858600|ref|NC_013766.2|:997453-998568
hypothetical protein LM5578 1006	gi|284801256|ref|YP 003413121.1	gi|662858600|ref|NC_013766.2|:998591-999247
hypothetical protein LM5578 1007	gi|284801257|ref|YP 003413122.1	gi|662858600|ref|NC_013766.2|:c1001239-999278
hypothetical protein LM5578 1009	gi|284801259|ref|YP 003413124.1	gi|662858600|ref|NC_013766.2|:1001497-1002120
hypothetical protein LM5578 1010	gi|284801260|ref|YP 003413125.1	gi|662858600|ref|NC_013766.2|:1002229-1002897
hypothetical protein LM5578 1013	gi|284801263|ref|YP 003413128.1	gi|662858600|ref|NC_013766.2|:c1005408-1004803
hypothetical protein LM5578 1017	gi|284801267|ref|YP 003413132.1	gi|662858600|ref|NC_013766.2|:1008703-1009443
hypothetical protein LM5578 1029	gi|284801279|ref|YP 003413144.1	gi|662858600|ref|NC_013766.2|:c1019631-1018942
hypothetical protein LM5578 1032	gi|284801282|ref|YP 003413147.1	gi|662858600|ref|NC_013766.2|:c1022549-1021728
hypothetical protein LM5578 1039	gi|284801289|ref|YP 003413154.1	gi|662858600|ref|NC_013766.2|:1026952-1027674
hypothetical protein LM5578 1040	gi|284801290|ref|YP 003413155.1	gi|662858600|ref|NC_013766.2|:1027794-1028849
hypothetical protein LM5578 1041	gi|284801291|ref|YP 003413156.1	gi|662858600|ref|NC_013766.2|:1028962-1029870

hypothetical protein LM5578 1042	gi|284801292|ref|YP 003413157.1	gi|662858600|ref|NC_013766.2|:1029884-1031110
hypothetical protein LM5578 1048	gi|284801298|ref|YP 003413163.1	gi|662858600|ref|NC_013766.2|:1035006-1035674
hypothetical protein LM5578 1050	gi|284801300|ref|YP 003413165.1	gi|662858600|ref|NC_013766.2|:1036519-1037409
DltD protein for D-alanine esterification of lipoteichoic acid and wall teichoic acid	gi|284801302|ref|YP 003413167.1	gi|662858600|ref|NC_013766.2|:c1039577-1038303
hypothetical protein LM5578 1059	gi|284801309|ref|YP 003413174.1	gi|662858600|ref|NC_013766.2|:c1044863-1044114
hypothetical protein LM5578 1061	gi|284801311|ref|YP 003413176.1	gi|662858600|ref|NC_013766.2|:1046299-1047063
hypothetical protein LM5578 1064	gi|284801314|ref|YP 003413179.1	gi|662858600|ref|NC_013766.2|:1049491-1050513
hypothetical protein LM5578 1068	gi|284801318|ref|YP 003413183.1	gi|662858600|ref|NC_013766.2|:1052016-1052927
peptide chain release factor 3	gi|284801320|ref|YP 003413185.1	gi|662858600|ref|NC_013766.2|:1053856-1055424
ATP-dependent protease	gi|284801329|ref|YP 003413194.1	gi|662858600|ref|NC_013766.2|:c1065032-1062858
hypothetical protein LM5578 1081	gi|284801331|ref|YP 003413196.1	gi|662858600|ref|NC_013766.2|:1065952-1066743
hypothetical protein LM5578 1082	gi|284801332|ref|YP 003413197.1	gi|662858600|ref|NC_013766.2|:c1068210-1066744
phosphotransferase system enzyme I	gi|284801335|ref|YP 003413200.1	gi|662858600|ref|NC_013766.2|:1069177-1070895
hypothetical protein LM5578 1087	gi|284801337|ref|YP 003413202.1	gi|662858600|ref|NC_013766.2|:1072055-1072915
hypothetical protein LM5578 1088	gi|284801338|ref|YP	gi|662858600|ref|NC_013766.2|:c1074103-1072958

	003413203.1	
hypothetical protein LM5578 1092	gi|284801342|ref|YP 003413207.1	gi|662858600|ref|NC_013766.2|:1075732-1076610
hypothetical protein LM5578 1093	gi|284801343|ref|YP 003413208.1	gi|662858600|ref|NC_013766.2|:1076658-1077368
hypothetical protein LM5578 1094	gi|284801344|ref|YP 003413209.1	gi|662858600|ref|NC_013766.2|:1077432-1078547
hypothetical protein LM5578 1095	gi|284801345|ref|YP 003413210.1	gi|662858600|ref|NC_013766.2|:c1079472-1078591
hypothetical protein LM5578 1096	gi|284801346|ref|YP 003413211.1	gi|662858600|ref|NC_013766.2|:1079807-1081000
hypothetical protein LM5578 1097	gi|284801347|ref|YP 003413212.1	gi|662858600|ref|NC_013766.2|:1080993-1081841
hypothetical protein LM5578 1102	gi|284801352|ref|YP 003413217.1	gi|662858600|ref|NC_013766.2|:1085257-1085970
hypothetical protein LM5578 1103	gi|284801353|ref|YP 003413218.1	gi|662858600|ref|NC_013766.2|:1085967-1087025
hypothetical protein LM5578 1104	gi|284801354|ref|YP 003413219.1	gi|662858600|ref|NC_013766.2|:1087022-1087660
hypothetical protein LM5578 1105	gi|284801355|ref|YP 003413220.1	gi|662858600|ref|NC_013766.2|:1087679-1088338
hypothetical protein LM5578 1106	gi|284801356|ref|YP 003413221.1	gi|662858600|ref|NC_013766.2|:1088493-1089413
hypothetical protein LM5578 1108	gi|284801358|ref|YP 003413223.1	gi|662858600|ref|NC_013766.2|:c1091268-1090303
hypothetical protein LM5578 1109	gi|284801359|ref|YP 003413224.1	gi|662858600|ref|NC_013766.2|:c1093044-1091377
hypothetical protein LM5578 1111	gi|284801361|ref|YP 003413226.1	gi|662858600|ref|NC_013766.2|:1093745-1094515

hypothetical protein LM5578 1122	gi|284801371|ref|YP 003413236.1	gi|662858600|ref|NC_013766.2|:c1105801-1105139
hypothetical protein LM5578 1124	gi|284801373|ref|YP 003413238.1	gi|662858600|ref|NC_013766.2|:1106580-1107350
hypothetical protein LM5578 1125	gi|284801374|ref|YP 003413239.1	gi|662858600|ref|NC_013766.2|:1107445-1108668
hypothetical protein LM5578 1132	gi|284801381|ref|YP 003413246.1	gi|662858600|ref|NC_013766.2|:c1112841-1111843
hypothetical protein LM5578 1133	gi|284801382|ref|YP 003413247.1	gi|662858600|ref|NC_013766.2|:1112972-1113526
peptide deformylase	gi|284801383|ref|YP 003413248.1	gi|662858600|ref|NC_013766.2|:c1114126-1113575
hypothetical protein LM5578 1135	gi|284801384|ref|YP 003413249.1	gi|662858600|ref|NC_013766.2|:1114960-1116075
dihydrolipoamide acetyltransferase	gi|284801386|ref|YP 003413251.1	gi|662858600|ref|NC_013766.2|:1117166-1118800
hypothetical protein LM5578 1140	gi|284801389|ref|YP 003413254.1	gi|662858600|ref|NC_013766.2|:1120836-1121789
hypothetical protein LM5578 1147	gi|284801396|ref|YP 003413261.1	gi|662858600|ref|NC_013766.2|:c1128037-1127093
hypothetical protein LM5578 1148	gi|284801397|ref|YP 003413262.1	gi|662858600|ref|NC_013766.2|:c1128852-1128238
hypothetical protein LM5578 1150	gi|284801399|ref|YP 003413264.1	gi|662858600|ref|NC_013766.2|:1129948-1131786
hypothetical protein LM5578 1154	gi|284801403|ref|YP 003413268.1	gi|662858600|ref|NC_013766.2|:1133762-1134970
pyruvate carboxylase	gi|284801404|ref|YP 003413269.1	gi|662858600|ref|NC_013766.2|:1135248-1138688
hypothetical protein LM5578 1156	gi|284801405|ref|YP 003413270.1	gi|662858600|ref|NC_013766.2|:1138840-1139739

NAD synthetase	gi|284801425|ref|YP 003413290.1	gi|662858600|ref|NC_013766.2|:1164188-1165012
hypothetical protein LM5578 1204	gi|284801451|ref|YP 003413316.1	gi|662858600|ref|NC_013766.2|:1188827-1190542
ATP-dependent Clp protease proteolytic subunit	gi|284801459|ref|YP 003413324.1	gi|662858600|ref|NC_013766.2|:c1197311-1196739
hypothetical protein LM5578 1215	gi|284801462|ref|YP 003413327.1	gi|662858600|ref|NC_013766.2|:1198546-1199901
hypothetical protein LM5578 1219	gi|284801466|ref|YP 003413331.1	gi|662858600|ref|NC_013766.2|:1201412-1202161
hypothetical protein LM5578 1221	gi|284801468|ref|YP 003413333.1	gi|662858600|ref|NC_013766.2|:1202717-1203463
hypothetical protein LM5578 1222	gi|284801469|ref|YP 003413334.1	gi|662858600|ref|NC_013766.2|:1203464-1204039
hypothetical protein LM5578 1225	gi|284801472|ref|YP 003413337.1	gi|662858600|ref|NC_013766.2|:1205914-1206717
hypothetical protein LM5578 1226	gi|284801473|ref|YP 003413338.1	gi|662858600|ref|NC_013766.2|:1206736-1208400
hypothetical protein LM5578 1227	gi|284801474|ref|YP 003413339.1	gi|662858600|ref|NC_013766.2|:1208438-1209097
hypothetical protein LM5578 1233	gi|284801480|ref|YP 003413345.1	gi|662858600|ref|NC_013766.2|:1212589-1213224
ATP:cob(I)alamin adenosyltransferase protein PduO	gi|284801484|ref|YP 003413349.1	gi|662858600|ref|NC_013766.2|:1214843-1215838
hypothetical protein LM5578 1239	gi|284801486|ref|YP 003413351.1	gi|662858600|ref|NC_013766.2|:1217270-1218388
hypothetical protein LM5578 1241	gi|284801488|ref|YP 003413353.1	gi|662858600|ref|NC_013766.2|:1219186-1220379
threonine-phosphate decarboxylase	gi|284801489|ref|YP	gi|662858600|ref|NC_013766.2|:1220393-1221478

	003413354.1	
hypothetical protein LM5578 1243	gi|284801490|ref|YP 003413355.1	gi|662858600|ref|NC_013766.2|:1221468-1222343
hypothetical protein LM5578 1245	gi|284801492|ref|YP 003413357.1	gi|662858600|ref|NC_013766.2|:1224296-1224928
hypothetical protein LM5578 1248	gi|284801495|ref|YP 003413360.1	gi|662858600|ref|NC_013766.2|:1227930-1229294
ethanolamine ammonia-lyase small subunit	gi|284801496|ref|YP 003413361.1	gi|662858600|ref|NC_013766.2|:1229314-1230195
hypothetical protein LM5578 1250	gi|284801497|ref|YP 003413362.1	gi|662858600|ref|NC_013766.2|:1230218-1230871
hypothetical protein LM5578 1252	gi|284801499|ref|YP 003413364.1	gi|662858600|ref|NC_013766.2|:1231407-1232867
hypothetical protein LM5578 1254	gi|284801501|ref|YP 003413366.1	gi|662858600|ref|NC_013766.2|:1233349-1234104
hypothetical protein LM5578 1255	gi|284801502|ref|YP 003413367.1	gi|662858600|ref|NC_013766.2|:1234117-1234758
hypothetical protein LM5578 1256	gi|284801503|ref|YP 003413368.1	gi|662858600|ref|NC_013766.2|:1234755-1235327
hypothetical protein LM5578 1258	gi|284801505|ref|YP 003413370.1	gi|662858600|ref|NC_013766.2|:1235604-1236149
precorrin-8X methylmutase	gi|284801513|ref|YP 003413378.1	gi|662858600|ref|NC_013766.2|:1243430-1244062
cobalt-precorrin-6Y C(5)-methyltransferase	gi|284801515|ref|YP 003413380.1	gi|662858600|ref|NC_013766.2|:1245196-1245792
hypothetical protein LM5578 1274	gi|284801521|ref|YP 003413386.1	gi|662858600|ref|NC_013766.2|:1249595-1251076
hypothetical protein LM5578 1275	gi|284801522|ref|YP 003413387.1	gi|662858600|ref|NC_013766.2|:1251073-1251858

cobalt-precorrin-2 C(20)-methyltransferase	gi|284801523|ref|YP 003413388.1	gi|662858600|ref|NC_013766.2|:1251851-1252561
cobalt transport protein CbiM	gi|284801524|ref|YP 003413389.1	gi|662858600|ref|NC_013766.2|:1252558-1253292
hypothetical protein LM5578 1279	gi|284801526|ref|YP 003413391.1	gi|662858600|ref|NC_013766.2|:1253548-1254249
hypothetical protein LM5578 1289	gi|284801536|ref|YP 003413401.1	gi|662858600|ref|NC_013766.2|:1261102-1262115
hypothetical protein LM5578 1296	gi|284801543|ref|YP 003413408.1	gi|662858600|ref|NC_013766.2|:1268963-1269664
uracil-DNA glycosylase	gi|284801547|ref|YP 003413412.1	gi|662858600|ref|NC_013766.2|:1276961-1277635
ribonuclease HIII	gi|284801548|ref|YP 003413413.1	gi|662858600|ref|NC_013766.2|:c1278554-1277673
hypothetical protein LM5578 1303	gi|284801550|ref|YP 003413415.1	gi|662858600|ref|NC_013766.2|:1279016-1279558
hypothetical protein LM5578 1304	gi|284801551|ref|YP 003413416.1	gi|662858600|ref|NC_013766.2|:1279651-1281363
recombination and DNA strand exchange inhibitor protein	gi|284801552|ref|YP 003413417.1	gi|662858600|ref|NC_013766.2|:1281386-1283743
aspartate kinase	gi|284801555|ref|YP 003413420.1	gi|662858600|ref|NC_013766.2|:1286211-1287425
hypothetical protein LM5578 1313	gi|284801560|ref|YP 003413425.1	gi|662858600|ref|NC_013766.2|:1290334-1290858
hypothetical protein LM5578 1376	gi|284801623|ref|YP 003413488.1	gi|662858600|ref|NC_013766.2|:1336156-1337514
hypothetical protein LM5578 1385	gi|284801632|ref|YP 003413497.1	gi|662858600|ref|NC_013766.2|:c1343718-1342513
hypothetical protein LM5578 1386	gi|284801633|ref|YP 003413498.1	gi|662858600|ref|NC_013766.2|:1343923-1344582

hypothetical protein LM5578 1392	gi|284801639|ref|YP 003413504.1	gi|662858600|ref|NC_013766.2|:c1348743-1347097
gamma-glutamyl kinase	gi|284801645|ref|YP 003413510.1	gi|662858600|ref|NC_013766.2|:c1354515-1353685
hypothetical protein LM5578 1399	gi|284801646|ref|YP 003413511.1	gi|662858600|ref|NC_013766.2|:c1355790-1354651
trigger factor	gi|284801652|ref|YP 003413517.1	gi|662858600|ref|NC_013766.2|:1359282-1360565
ATP-dependent protease ATP-binding subunit	gi|284801653|ref|YP 003413518.1	gi|662858600|ref|NC_013766.2|:1360750-1362009
hypothetical protein LM5578 1407	gi|284801654|ref|YP 003413519.1	gi|662858600|ref|NC_013766.2|:1362128-1362694
hypothetical protein LM5578 1408	gi|284801655|ref|YP 003413520.1	gi|662858600|ref|NC_013766.2|:1362729-1363298
ribosomal biogenesis GTPase	gi|284801657|ref|YP 003413522.1	gi|662858600|ref|NC_013766.2|:1363952-1364815
DNA topoisomerase I	gi|284801660|ref|YP 003413525.1	gi|662858600|ref|NC_013766.2|:1366863-1368941
hypothetical protein LM5578 1415	gi|284801662|ref|YP 003413527.1	gi|662858600|ref|NC_013766.2|:1370591-1371493
ATP-dependent protease peptidase subunit	gi|284801663|ref|YP 003413528.1	gi|662858600|ref|NC_013766.2|:1371514-1372053
transcriptional repressor CodY	gi|284801665|ref|YP 003413530.1	gi|662858600|ref|NC_013766.2|:1373497-1374276
hypothetical protein LM5578 1421	gi|284801668|ref|YP 003413533.1	gi|662858600|ref|NC_013766.2|:1375135-1376007
DNA topoisomerase IV subunit A	gi|284801672|ref|YP 003413537.1	gi|662858600|ref|NC_013766.2|:1379355-1381814
hypothetical protein LM5578 1428	gi|284801675|ref|YP 003413540.1	gi|662858600|ref|NC_013766.2|:1384756-1386552

hypothetical protein LM5578 1431	gi|284801678|ref|YP 003413543.1	gi|662858600|ref|NC_013766.2|:1389598-1391274
hypothetical protein LM5578 1432	gi|284801679|ref|YP 003413544.1	gi|662858600|ref|NC_013766.2|:1391400-1392317
hypothetical protein LM5578 1434	gi|284801681|ref|YP 003413546.1	gi|662858600|ref|NC_013766.2|:1392778-1394007
hypothetical protein LM5578 1435	gi|284801682|ref|YP 003413547.1	gi|662858600|ref|NC_013766.2|:1394000-1395226
hypothetical protein LM5578 1437	gi|284801684|ref|YP 003413549.1	gi|662858600|ref|NC_013766.2|:1395868-1397202
LexA repressor	gi|284801687|ref|YP 003413552.1	gi|662858600|ref|NC_013766.2|:c1399850-1399236
hypothetical protein LM5578 1450	gi|284801696|ref|YP 003413561.1	gi|662858600|ref|NC_013766.2|:c1407931-1406732
hypothetical protein LM5578 1452	gi|284801698|ref|YP 003413563.1	gi|662858600|ref|NC_013766.2|:1408582-1409310
hypothetical protein LM5578 1453	gi|284801699|ref|YP 003413564.1	gi|662858600|ref|NC_013766.2|:1409310-1409867
hypothetical protein LM5578 1457	gi|284801703|ref|YP 003413568.1	gi|662858600|ref|NC_013766.2|:1412828-1414090
prolyl-tRNA synthetase	gi|284801704|ref|YP 003413569.1	gi|662858600|ref|NC_013766.2|:1414130-1415836
hypothetical protein LM5578 1467	gi|284801713|ref|YP 003413578.1	gi|662858600|ref|NC_013766.2|:1425761-1426675
hypothetical protein LM5578 1468	gi|284801714|ref|YP 003413579.1	gi|662858600|ref|NC_013766.2|:1426745-1427689
hypothetical protein LM5578 1471	gi|284801717|ref|YP 003413582.1	gi|662858600|ref|NC_013766.2|:c1431596-1430556
hypothetical protein LM5578 1475	gi|284801721|ref|YP 003413586.1	gi|662858600|ref|NC_013766.2|:1433023-1433562

hypothetical protein LM5578 1476	gi|284801722|ref|YP 003413587.1	gi|662858600|ref|NC_013766.2|:1433661-1435199
hypothetical protein LM5578 1478	gi|284801724|ref|YP 003413589.1	gi|662858600|ref|NC_013766.2|:1435449-1436417
hypothetical protein LM5578 1479	gi|284801725|ref|YP 003413590.1	gi|662858600|ref|NC_013766.2|:1436551-1437642
glycine cleavage system aminomethyltransferase T	gi|284801733|ref|YP 003413598.1	gi|662858600|ref|NC_013766.2|:1441941-1443095
hypothetical protein LM5578 1493	gi|284801739|ref|YP 003413604.1	gi|662858600|ref|NC_013766.2|:1447832-1448893
elongation factor P	gi|284801740|ref|YP 003413605.1	gi|662858600|ref|NC_013766.2|:1448996-1449553
hypothetical protein LM5578 1495	gi|284801741|ref|YP 003413606.1	gi|662858600|ref|NC_013766.2|:1449712-1450179
acetyl-CoA carboxylase biotin carboxylase subunit	gi|284801742|ref|YP 003413607.1	gi|662858600|ref|NC_013766.2|:1450193-1451557
hypothetical protein LM5578 1499	gi|284801745|ref|YP 003413610.1	gi|662858600|ref|NC_013766.2|:1452481-1453335
exodeoxyribonuclease VII large subunit	gi|284801746|ref|YP 003413611.1	gi|662858600|ref|NC_013766.2|:1453354-1454706
hypothetical protein LM5578 1502	gi|284801748|ref|YP 003413613.1	gi|662858600|ref|NC_013766.2|:1454926-1455807
1-deoxy-D-xylulose-5-phosphate synthase	gi|284801750|ref|YP 003413615.1	gi|662858600|ref|NC_013766.2|:1456421-1458202
hypothetical protein LM5578 1505	gi|284801751|ref|YP 003413616.1	gi|662858600|ref|NC_013766.2|:1458199-1459023
DNA repair and genetic recombination	gi|284801753|ref|YP 003413618.1	gi|662858600|ref|NC_013766.2|:1459686-1461377
hypothetical protein LM5578 1508	gi|284801754|ref|YP 003413619.1	gi|662858600|ref|NC_013766.2|:1461506-1462372

dihydrolipoamide dehydrogenase	gi|284801756|ref|YP 003413621.1	gi|662858600|ref|NC_013766.2|:1463592-1465019
6-phosphogluconate dehydrogenase	gi|284801762|ref|YP 003413627.1	gi|662858600|ref|NC_013766.2|:1469933-1471351
two-component response regulator	gi|284801763|ref|YP 003413628.1	gi|662858600|ref|NC_013766.2|:1471542-1472222
two-component sensor histidine kinase	gi|284801764|ref|YP 003413629.1	gi|662858600|ref|NC_013766.2|:1472219-1473670
hypothetical protein LM5578 1525	gi|284801770|ref|YP 003413635.1	gi|662858600|ref|NC_013766.2|:c1477846-1476851
hypothetical protein LM5578 1526	gi|284801771|ref|YP 003413636.1	gi|662858600|ref|NC_013766.2|:1478062-1478988
hypothetical protein LM5578 1527	gi|284801772|ref|YP 003413637.1	gi|662858600|ref|NC_013766.2|:1479140-1481413
pyrroline-5-carboxylate reductase	gi|284801773|ref|YP 003413638.1	gi|662858600|ref|NC_013766.2|:c1482286-1481447
CD4+ T cell-stimulating antigen, lipoprotein	gi|284801774|ref|YP 003413639.1	gi|662858600|ref|NC_013766.2|:1482638-1483711
hypothetical protein LM5578 1531	gi|284801776|ref|YP 003413641.1	gi|662858600|ref|NC_013766.2|:1485678-1486730
hypothetical protein LM5578 1532	gi|284801777|ref|YP 003413642.1	gi|662858600|ref|NC_013766.2|:1486727-1487677
hypothetical protein LM5578 1533	gi|284801778|ref|YP 003413643.1	gi|662858600|ref|NC_013766.2|:1487795-1489087
hypothetical protein LM5578 1534	gi|284801779|ref|YP 003413644.1	gi|662858600|ref|NC_013766.2|:1489068-1490354
hypothetical protein LM5578 1535	gi|284801780|ref|YP 003413645.1	gi|662858600|ref|NC_013766.2|:1490446-1491177
hypothetical protein LM5578 1536	gi|284801781|ref|YP 003413646.1	gi|662858600|ref|NC_013766.2|:1491228-1492157

hypothetical protein LM5578 1537	gi|284801782|ref|YP 003413647.1	gi|662858600|ref|NC_013766.2|:1492247-1492825
recombinase A	gi|284801784|ref|YP 003413649.1	gi|662858600|ref|NC_013766.2|:1494411-1495457
phosphodiesterase	gi|284801785|ref|YP 003413650.1	gi|662858600|ref|NC_013766.2|:1495758-1497320
hypothetical protein LM5578 1542	gi|284801787|ref|YP 003413652.1	gi|662858600|ref|NC_013766.2|:1497937-1498740
DNA mismatch repair protein	gi|284801789|ref|YP 003413654.1	gi|662858600|ref|NC_013766.2|:1499229-1501811
DNA mismatch repair protein	gi|284801790|ref|YP 003413655.1	gi|662858600|ref|NC_013766.2|:1501831-1503642
pyruvate formate-lyase	gi|284801792|ref|YP 003413657.1	gi|662858600|ref|NC_013766.2|:1504633-1506864
pyruvate-formate lyase activating enzyme	gi|284801793|ref|YP 003413658.1	gi|662858600|ref|NC_013766.2|:1506941-1507687
hypothetical protein LM5578 1549	gi|284801794|ref|YP 003413659.1	gi|662858600|ref|NC_013766.2|:c1508252-1507722
peptidoglycan binding protein	gi|284801799|ref|YP 003413664.1	gi|662858600|ref|NC_013766.2|:c1512685-1511366
hypothetical protein LM5578 1556	gi|284801801|ref|YP 003413666.1	gi|662858600|ref|NC_013766.2|:1514190-1515356
hypothetical protein LM5578 1558	gi|284801803|ref|YP 003413668.1	gi|662858600|ref|NC_013766.2|:c1517293-1516049
hypothetical protein LM5578 1559	gi|284801804|ref|YP 003413669.1	gi|662858600|ref|NC_013766.2|:c1518580-1517312
hypothetical protein LM5578 1560	gi|284801805|ref|YP 003413670.1	gi|662858600|ref|NC_013766.2|:c1519681-1518644
hypothetical protein LM5578 1562	gi|284801807|ref|YP 003413672.1	gi|662858600|ref|NC_013766.2|:1520810-1521796

hypothetical protein LM5578 1563	gi|284801808|ref|YP 003413673.1	gi|662858600|ref|NC_013766.2|:1521793-1523307
hypothetical protein LM5578 1564	gi|284801809|ref|YP 003413674.1	gi|662858600|ref|NC_013766.2|:c1524173-1523343
manganese transport protein MntH	gi|284801810|ref|YP 003413675.1	gi|662858600|ref|NC_013766.2|:c1525658-1524312
hypothetical protein LM5578 1566	gi|284801811|ref|YP 003413676.1	gi|662858600|ref|NC_013766.2|:c1526442-1525771
hypothetical protein LM5578 1567	gi|284801812|ref|YP 003413677.1	gi|662858600|ref|NC_013766.2|:c1527383-1526457
hypothetical protein LM5578 1568	gi|284801813|ref|YP 003413678.1	gi|662858600|ref|NC_013766.2|:c1528041-1527385
hypothetical protein LM5578 1570	gi|284801815|ref|YP 003413680.1	gi|662858600|ref|NC_013766.2|:c1530091-1529531
hypothetical protein LM5578 1573	gi|284801818|ref|YP 003413683.1	gi|662858600|ref|NC_013766.2|:c1533153-1532503
hypothetical protein LM5578 1574	gi|284801819|ref|YP 003413684.1	gi|662858600|ref|NC_013766.2|:c1534546-1533206
hypothetical protein LM5578 1575	gi|284801820|ref|YP 003413685.1	gi|662858600|ref|NC_013766.2|:c1536302-1534635
dihydrodipicolinate synthase	gi|284801821|ref|YP 003413686.1	gi|662858600|ref|NC_013766.2|:c1537203-1536322
aspartate kinase I	gi|284801822|ref|YP 003413687.1	gi|662858600|ref|NC_013766.2|:c1538429-1537218
hypothetical protein LM5578 1578	gi|284801823|ref|YP 003413688.1	gi|662858600|ref|NC_013766.2|:c1539484-1538441
hypothetical protein LM5578 1580	gi|284801825|ref|YP 003413690.1	gi|662858600|ref|NC_013766.2|:c1541847-1539682
hypothetical protein LM5578 1582	gi|284801827|ref|YP 003413692.1	gi|662858600|ref|NC_013766.2|:1542886-1543686

hypothetical protein LM5578 1584	gi|284801829|ref|YP 003413694.1	gi|662858600|ref|NC_013766.2|:1544941-1545648
metal (zinc) transport protein (ABC transporter, permease protein)	gi|284801833|ref|YP 003413698.1	gi|662858600|ref|NC_013766.2|:c1548352-1547474
endonuclease IV	gi|284801836|ref|YP 003413701.1	gi|662858600|ref|NC_013766.2|:c1551078-1550185
hypothetical protein LM5578 1592	gi|284801837|ref|YP 003413702.1	gi|662858600|ref|NC_013766.2|:c1552400-1551093
hypothetical protein LM5578 1594	gi|284801839|ref|YP 003413704.1	gi|662858600|ref|NC_013766.2|:c1554708-1553587
hypothetical protein LM5578 1595	gi|284801840|ref|YP 003413705.1	gi|662858600|ref|NC_013766.2|:c1555409-1554705
RNA polymerase sigma factor RpoD	gi|284801841|ref|YP 003413706.1	gi|662858600|ref|NC_013766.2|:c1556601-1555477
DNA primase	gi|284801842|ref|YP 003413707.1	gi|662858600|ref|NC_013766.2|:c1558576-1556696
hypothetical protein LM5578 1599	gi|284801844|ref|YP 003413709.1	gi|662858600|ref|NC_013766.2|:c1560050-1559226
DNA repair protein RecO	gi|284801847|ref|YP 003413712.1	gi|662858600|ref|NC_013766.2|:c1564082-1563315
hypothetical protein LM5578 1603	gi|284801848|ref|YP 003413713.1	gi|662858600|ref|NC_013766.2|:1564218-1564847
GTP-binding protein Era	gi|284801849|ref|YP 003413714.1	gi|662858600|ref|NC_013766.2|:c1565801-1564896
putative metalloprotease	gi|284801852|ref|YP 003413717.1	gi|662858600|ref|NC_013766.2|:c1567076-1566591
hypothetical protein LM5578 1609	gi|284801854|ref|YP 003413719.1	gi|662858600|ref|NC_013766.2|:c1570224-1569265
heat shock protein DnaJ	gi|284801859|ref|YP	gi|662858600|ref|NC_013766.2|:c1574167-1573034

	003413724.1	
class I heat-shock protein (molecular chaperone) DnaK	gi|284801860|ref|YP 003413725.1	gi|662858600|ref|NC_013766.2|:c1576150-1574309
heat shock protein GrpE	gi|284801861|ref|YP 003413726.1	gi|662858600|ref|NC_013766.2|:c1576759-1576184
heat-inducible transcription repressor	gi|284801862|ref|YP 003413727.1	gi|662858600|ref|NC_013766.2|:c1577838-1576801
DNA polymerase III subunit delta	gi|284801868|ref|YP 003413733.1	gi|662858600|ref|NC_013766.2|:c1584285-1583254
hypothetical protein LM5578 1625	gi|284801870|ref|YP 003413735.1	gi|662858600|ref|NC_013766.2|:c1587170-1586610
hypothetical protein LM5578 1626	gi|284801871|ref|YP 003413736.1	gi|662858600|ref|NC_013766.2|:c1587844-1587242
hypothetical protein LM5578 1627	gi|284801872|ref|YP 003413737.1	gi|662858600|ref|NC_013766.2|:c1588678-1587947
hypothetical protein LM5578 1632	gi|284801877|ref|YP 003413742.1	gi|662858600|ref|NC_013766.2|:c1591351-1590530
hypothetical protein LM5578 1634	gi|284801879|ref|YP 003413744.1	gi|662858600|ref|NC_013766.2|:c1592989-1592468
hypothetical protein LM5578 1639	gi|284801884|ref|YP 003413749.1	gi|662858600|ref|NC_013766.2|:c1597641-1597012
uridine kinase	gi|284801887|ref|YP 003413752.1	gi|662858600|ref|NC_013766.2|:c1598991-1598362
hypothetical protein LM5578 1644	gi|284801889|ref|YP 003413754.1	gi|662858600|ref|NC_013766.2|:c1600788-1599718
hypothetical protein LM5578 1645	gi|284801890|ref|YP 003413755.1	gi|662858600|ref|NC_013766.2|:1600991-1601617
alanyl-tRNA synthetase	gi|284801894|ref|YP 003413759.1	gi|662858600|ref|NC_013766.2|:c1605255-1602757

hypothetical protein LM5578 1650	gi|284801895|ref|YP 003413760.1	gi|662858600|ref|NC_013766.2|:c1606398-1605703
hypothetical protein LM5578 1652	gi|284801897|ref|YP 003413762.1	gi|662858600|ref|NC_013766.2|:1608079-1608765
hypothetical protein LM5578 1653	gi|284801898|ref|YP 003413763.1	gi|662858600|ref|NC_013766.2|:1608762-1610201
hypothetical protein LM5578 1655	gi|284801900|ref|YP 003413765.1	gi|662858600|ref|NC_013766.2|:c1613335-1612667
hypothetical protein LM5578 1657	gi|284801902|ref|YP 003413767.1	gi|662858600|ref|NC_013766.2|:c1615319-1614204
hypothetical protein LM5578 1658	gi|284801903|ref|YP 003413768.1	gi|662858600|ref|NC_013766.2|:c1616486-1615338
recombination factor protein RarA	gi|284801904|ref|YP 003413769.1	gi|662858600|ref|NC_013766.2|:c1618149-1616866
hypothetical protein LM5578 1661	gi|284801906|ref|YP 003413771.1	gi|662858600|ref|NC_013766.2|:1618965-1620170
hypothetical protein LM5578 1666	gi|284801911|ref|YP 003413776.1	gi|662858600|ref|NC_013766.2|:1624624-1625907
hypothetical protein LM5578 1668	gi|284801913|ref|YP 003413778.1	gi|662858600|ref|NC_013766.2|:c1628628-1626412
hypothetical protein LM5578 1670	gi|284801915|ref|YP 003413780.1	gi|662858600|ref|NC_013766.2|:c1631780-1629345
bifunctional preprotein translocase subunit SecD/SecF	gi|284801917|ref|YP 003413782.1	gi|662858600|ref|NC_013766.2|:c1634506-1632242
queuine tRNA-ribosyltransferase	gi|284801920|ref|YP 003413785.1	gi|662858600|ref|NC_013766.2|:c1636542-1635403
hypothetical protein LM5578 1676	gi|284801921|ref|YP 003413786.1	gi|662858600|ref|NC_013766.2|:c1637657-1636629
Holliday junction DNA helicase B	gi|284801922|ref|YP 003413787.1	gi|662858600|ref|NC_013766.2|:c1638668-1637661

hypothetical protein LM5578 1678	gi|284801923|ref|YP 003413788.1	gi|662858600|ref|NC_013766.2|:c1639289-1638684
prephenate dehydratase	gi|284801926|ref|YP 003413791.1	gi|662858600|ref|NC_013766.2|:c1642123-1641254
GTPase ObgE	gi|284801927|ref|YP 003413792.1	gi|662858600|ref|NC_013766.2|:c1643458-1642169
glycerol kinase	gi|284801928|ref|YP 003413793.1	gi|662858600|ref|NC_013766.2|:c1645111-1643618
hypothetical protein LM5578 1684	gi|284801929|ref|YP 003413794.1	gi|662858600|ref|NC_013766.2|:c1646004-1645186
hypothetical protein LM5578 1688	gi|284801933|ref|YP 003413798.1	gi|662858600|ref|NC_013766.2|:c1648837-1647476
septum formation inhibitor	gi|284801935|ref|YP 003413800.1	gi|662858600|ref|NC_013766.2|:c1650369-1649692
hypothetical protein LM5578 1691	gi|284801936|ref|YP 003413801.1	gi|662858600|ref|NC_013766.2|:c1651065-1650547
hypothetical protein LM5578 1692	gi|284801937|ref|YP 003413802.1	gi|662858600|ref|NC_013766.2|:c1651955-1651068
hypothetical protein LM5578 1693	gi|284801938|ref|YP 003413803.1	gi|662858600|ref|NC_013766.2|:c1653054-1652041
DNA repair protein RadC	gi|284801939|ref|YP 003413804.1	gi|662858600|ref|NC_013766.2|:c1654180-1653506
valyl-tRNA synthetase	gi|284801943|ref|YP 003413808.1	gi|662858600|ref|NC_013766.2|:c1659116-1656465
glutamate-1-semialdehyde aminotransferase	gi|284801944|ref|YP 003413809.1	gi|662858600|ref|NC_013766.2|:c1660728-1659439
delta-aminolevulinic acid dehydratase	gi|284801945|ref|YP 003413810.1	gi|662858600|ref|NC_013766.2|:c1661715-1660741
uroporphyrinogen-III synthase	gi|284801946|ref|YP 003413811.1	gi|662858600|ref|NC_013766.2|:c1662434-1661712

porphobilinogen deaminase	gi|284801947|ref|YP 003413812.1	gi|662858600|ref|NC_013766.2|:c1663360-1662431
glutamyl-tRNA reductase	gi|284801948|ref|YP 003413813.1	gi|662858600|ref|NC_013766.2|:c1664671-1663364
GTPase EngB	gi|284801949|ref|YP 003413814.1	gi|662858600|ref|NC_013766.2|:c1665383-1664799
hypothetical protein LM5578 1709	gi|284801954|ref|YP 003413819.1	gi|662858600|ref|NC_013766.2|:c1671279-1670620
hypothetical protein LM5578 1710	gi|284801955|ref|YP 003413820.1	gi|662858600|ref|NC_013766.2|:c1672060-1671239
methylcitrate synthase	gi|284801958|ref|YP 003413823.1	gi|662858600|ref|NC_013766.2|:c1677280-1676159
pyruvate kinase	gi|284801961|ref|YP 003413826.1	gi|662858600|ref|NC_013766.2|:c1680186-1678429
6-phosphofructokinase	gi|284801962|ref|YP 003413827.1	gi|662858600|ref|NC_013766.2|:c1681428-1680469
acetyl-CoA carboxylase carboxyltransferase subunit alpha	gi|284801963|ref|YP 003413828.1	gi|662858600|ref|NC_013766.2|:c1682668-1681712
hypothetical protein LM5578 1721	gi|284801966|ref|YP 003413831.1	gi|662858600|ref|NC_013766.2|:c1688146-1687208
hypothetical protein LM5578 1725	gi|284801970|ref|YP 003413835.1	gi|662858600|ref|NC_013766.2|:c1692606-1691494
inorganic polyphosphate/ATP-NAD kinase	gi|284801978|ref|YP 003413843.1	gi|662858600|ref|NC_013766.2|:1698076-1698879
ornithine carbamoyltransferase	gi|284801979|ref|YP 003413844.1	gi|662858600|ref|NC_013766.2|:c1699861-1698911
bifunctional ornithine acetyltransferase/N-acetylglutamate synthase protein	gi|284801982|ref|YP 003413847.1	gi|662858600|ref|NC_013766.2|:c1702976-1701780
N-acetyl-gamma-glutamyl-phosphate	gi|284801983|ref|YP	gi|662858600|ref|NC_013766.2|:c1704023-1702992

reductase	003413848.1	
hypothetical protein LM5578 1739	gi|284801984|ref|YP 003413849.1	gi|662858600|ref|NC_013766.2|:c1705408-1704197
hypothetical protein LM5578 1740	gi|284801985|ref|YP 003413850.1	gi|662858600|ref|NC_013766.2|:c1706549-1705410
septation ring formation regulator EzrA	gi|284801986|ref|YP 003413851.1	gi|662858600|ref|NC_013766.2|:c1708391-1706676
30S ribosomal protein S4	gi|284801988|ref|YP 003413853.1	gi|662858600|ref|NC_013766.2|:1709384-1709986
hypothetical protein LM5578 1748	gi|284801993|ref|YP 003413858.1	gi|662858600|ref|NC_013766.2|:c1716201-1715677
hypothetical protein LM5578 1755	gi|284802000|ref|YP 003413865.1	gi|662858600|ref|NC_013766.2|:c1724498-1723698
hypothetical protein LM5578 1758	gi|284802003|ref|YP 003413868.1	gi|662858600|ref|NC_013766.2|:c1726240-1725167
hypothetical protein LM5578 1761	gi|284802006|ref|YP 003413871.1	gi|662858600|ref|NC_013766.2|:c1728020-1727169
tRNA (guanine-N(7))-methyltransferase	gi|284802007|ref|YP 003413872.1	gi|662858600|ref|NC_013766.2|:c1728725-1728081
hypothetical protein LM5578 1763	gi|284802008|ref|YP 003413873.1	gi|662858600|ref|NC_013766.2|:c1729514-1728732
D-amino acid aminotransferase	gi|284802011|ref|YP 003413876.1	gi|662858600|ref|NC_013766.2|:c1732723-1731854
dipeptidase PepV	gi|284802012|ref|YP 003413877.1	gi|662858600|ref|NC_013766.2|:c1734248-1732836
hypothetical protein LM5578 1769	gi|284802014|ref|YP 003413879.1	gi|662858600|ref|NC_013766.2|:1734841-1735671
hypothetical protein LM5578 1770	gi|284802015|ref|YP 003413880.1	gi|662858600|ref|NC_013766.2|:c1736363-1735710

hypothetical protein LM5578 1771	gi|284802016|ref|YP 003413881.1	gi|662858600|ref|NC_013766.2|:c1737986-1736373
tryptophan synthase subunit alpha	gi|284802019|ref|YP 003413884.1	gi|662858600|ref|NC_013766.2|:c1741076-1740303
tryptophan synthase subunit beta	gi|284802020|ref|YP 003413885.1	gi|662858600|ref|NC_013766.2|:c1742271-1741069
phosphoribosyl anthranilate isomerase	gi|284802021|ref|YP 003413886.1	gi|662858600|ref|NC_013766.2|:c1742882-1742274
indole-3-glycerol-phosphate synthase	gi|284802022|ref|YP 003413887.1	gi|662858600|ref|NC_013766.2|:c1743637-1742879
anthranilate phosphoribosyltransferase	gi|284802023|ref|YP 003413888.1	gi|662858600|ref|NC_013766.2|:c1744653-1743634
anthranilate synthase component II	gi|284802024|ref|YP 003413889.1	gi|662858600|ref|NC_013766.2|:c1745230-1744625
anthranilate synthase component I	gi|284802025|ref|YP 003413890.1	gi|662858600|ref|NC_013766.2|:c1746591-1745227
bifunctional acetaldehyde-CoA/alcohol dehydrogenase	gi|284802026|ref|YP 003413891.1	gi|662858600|ref|NC_013766.2|:1747675-1750275
hypothetical protein LM5578 1783	gi|284802028|ref|YP 003413893.1	gi|662858600|ref|NC_013766.2|:1750910-1751830
hypothetical protein LM5578 1784	gi|284802029|ref|YP 003413894.1	gi|662858600|ref|NC_013766.2|:1751827-1752894
aconitate hydratase	gi|284802033|ref|YP 003413898.1	gi|662858600|ref|NC_013766.2|:c1757678-1754829
hypothetical protein LM5578 1794	gi|284802039|ref|YP 003413904.1	gi|662858600|ref|NC_013766.2|:1766546-1767154
hypothetical protein LM5578 1796	gi|284802041|ref|YP 003413906.1	gi|662858600|ref|NC_013766.2|:c1768257-1767742
elongation factor Ts	gi|284802049|ref|YP 003413914.1	gi|662858600|ref|NC_013766.2|:c1777354-1776470

30S ribosomal protein S2	gi|284802050|ref|YP 003413915.1	gi|662858600|ref|NC_013766.2|:c1778183-1777434
hypothetical protein LM5578 1809	gi|284802054|ref|YP 003413919.1	gi|662858600|ref|NC_013766.2|:1783104-1783679
hypothetical protein LM5578 1814	gi|284802059|ref|YP 003413924.1	gi|662858600|ref|NC_013766.2|:c1793810-1792902
hypothetical protein LM5578 1816	gi|284802061|ref|YP 003413926.1	gi|662858600|ref|NC_013766.2|:c1794593-1794120
hypothetical protein LM5578 1821	gi|284802066|ref|YP 003413931.1	gi|662858600|ref|NC_013766.2|:c1799122-1798295
1,4-dihydroxy-2-naphthoate octaprenyltransferase	gi|284802069|ref|YP 003413934.1	gi|662858600|ref|NC_013766.2|:1802426-1803364
cystathionine beta-lyase	gi|284802071|ref|YP 003413936.1	gi|662858600|ref|NC_013766.2|:c1806419-1805247
hypothetical protein LM5578 1827	gi|284802072|ref|YP 003413937.1	gi|662858600|ref|NC_013766.2|:c1807536-1806412
5-methyltetrahydropteroyltriglutamate--homocysteine methyltransferase	gi|284802073|ref|YP 003413938.1	gi|662858600|ref|NC_013766.2|:c1809855-1807558
hypothetical protein LM5578 1829	gi|284802074|ref|YP 003413939.1	gi|662858600|ref|NC_013766.2|:c1811320-1810145
hypothetical protein LM5578 1831	gi|284802076|ref|YP 003413941.1	gi|662858600|ref|NC_013766.2|:c1820204-1819263
glutamate-1-semialdehyde aminotransferase	gi|284802077|ref|YP 003413942.1	gi|662858600|ref|NC_013766.2|:1820349-1821647
hypothetical protein LM5578 1837	gi|284802082|ref|YP 003413947.1	gi|662858600|ref|NC_013766.2|:1825614-1826594
hypothetical protein LM5578 1842	gi|284802087|ref|YP 003413952.1	gi|662858600|ref|NC_013766.2|:c1831761-1829164

hypothetical protein LM5578 1843	gi|284802088|ref|YP 003413953.1	gi|662858600|ref|NC_013766.2|:c1832462-1831788
hypothetical protein LM5578 1845	gi|284802090|ref|YP 003413955.1	gi|662858600|ref|NC_013766.2|:c1834221-1833679
hypothetical protein LM5578 1846	gi|284802091|ref|YP 003413956.1	gi|662858600|ref|NC_013766.2|:1834411-1835313
hypothetical protein LM5578 1907	gi|284802152|ref|YP 003414017.1	gi|662858600|ref|NC_013766.2|:c1889794-1888925
methionine aminopeptidase	gi|284802155|ref|YP 003414020.1	gi|662858600|ref|NC_013766.2|:c1891811-1891053
hypothetical protein LM5578 1913	gi|284802158|ref|YP 003414023.1	gi|662858600|ref|NC_013766.2|:1893919-1895178
hypothetical protein LM5578 1918	gi|284802162|ref|YP 003414027.1	gi|662858600|ref|NC_013766.2|:c1899261-1898173
hypothetical protein LM5578 1926	gi|284802170|ref|YP 003414035.1	gi|662858600|ref|NC_013766.2|:c1907214-1906231
transcription activator of glutamate synthase operon GltC	gi|284802179|ref|YP 003414044.1	gi|662858600|ref|NC_013766.2|:1923505-1924392
hypothetical protein LM5578 1938	gi|284802182|ref|YP 003414047.1	gi|662858600|ref|NC_013766.2|:c1926922-1926107
hypothetical protein LM5578 1940	gi|284802184|ref|YP 003414049.1	gi|662858600|ref|NC_013766.2|:c1928247-1927582
hypothetical protein LM5578 1941	gi|284802185|ref|YP 003414050.1	gi|662858600|ref|NC_013766.2|:c1929661-1928621
hypothetical protein LM5578 1942	gi|284802186|ref|YP 003414051.1	gi|662858600|ref|NC_013766.2|:c1931422-1929683
hypothetical protein LM5578 1944	gi|284802188|ref|YP 003414053.1	gi|662858600|ref|NC_013766.2|:c1932796-1931921
hypothetical protein LM5578 1945	gi|284802189|ref|YP 003414054.1	gi|662858600|ref|NC_013766.2|:c1933544-1932867

hypothetical protein LM5578 1949	gi|284802193|ref|YP 003414058.1	gi|662858600|ref|NC_013766.2|:1937204-1937677
hypothetical protein LM5578 1950	gi|284802194|ref|YP 003414059.1	gi|662858600|ref|NC_013766.2|:c1938262-1937708
hypothetical protein LM5578 1951	gi|284802195|ref|YP 003414060.1	gi|662858600|ref|NC_013766.2|:c1939958-1938597
putative lipid kinase	gi|284802197|ref|YP 003414062.1	gi|662858600|ref|NC_013766.2|:c1941817-1940885
aspartyl/glutamyl-tRNA amidotransferase subunit B	gi|284802198|ref|YP 003414063.1	gi|662858600|ref|NC_013766.2|:c1943388-1941958
hypothetical protein LM5578 1957	gi|284802201|ref|YP 003414066.1	gi|662858600|ref|NC_013766.2|:c1946408-1945293
NAD-dependent DNA ligase LigA	gi|284802202|ref|YP 003414067.1	gi|662858600|ref|NC_013766.2|:c1948420-1946405
geranylgeranylglyceryl phosphate synthase-like protein	gi|284802204|ref|YP 003414069.1	gi|662858600|ref|NC_013766.2|:c1951382-1950705
hypothetical protein LM5578 1961	gi|284802205|ref|YP 003414070.1	gi|662858600|ref|NC_013766.2|:1951589-1952944
phosphoribosylamine--glycine ligase	gi|284802208|ref|YP 003414073.1	gi|662858600|ref|NC_013766.2|:c1954956-1953694
bifunctional phosphoribosylaminoimidazolecarboxamide formyltransferase/IMP cyclohydrolase	gi|284802209|ref|YP 003414074.1	gi|662858600|ref|NC_013766.2|:c1956510-1954981
hypothetical protein LM5578 1966	gi|284802210|ref|YP 003414075.1	gi|662858600|ref|NC_013766.2|:c1957082-1956516
phosphoribosylformylglycinamidine synthase II	gi|284802213|ref|YP 003414078.1	gi|662858600|ref|NC_013766.2|:c1961778-1959559
phosphoribosylaminoimidazole-succinocarboxamide synthase	gi|284802216|ref|YP 003414081.1	gi|662858600|ref|NC_013766.2|:c1963428-1962715
adenylosuccinate lyase	gi|284802217|ref|YP	gi|662858600|ref|NC_013766.2|:c1964801-1963509

	003414082.1	
phosphoribosylaminoimidazole carboxylase ATPase subunit	gi|284802218|ref|YP 003414083.1	gi|662858600|ref|NC_013766.2|:c1965944-1964820
hypothetical protein LM5578 1977	gi|284802221|ref|YP 003414086.1	gi|662858600|ref|NC_013766.2|:c1973149-1972544
hypothetical protein LM5578 1978	gi|284802222|ref|YP 003414087.1	gi|662858600|ref|NC_013766.2|:1973215-1973988
translation initiation factor IF-3	gi|284802229|ref|YP 003414094.1	gi|662858600|ref|NC_013766.2|:c1978243-1977740
hypothetical protein LM5578 1992	gi|284802236|ref|YP 003414101.1	gi|662858600|ref|NC_013766.2|:1981969-1982685
hypothetical protein LM5578 1994	gi|284802238|ref|YP 003414103.1	gi|662858600|ref|NC_013766.2|:c1983862-1983125
hypothetical protein LM5578 2000	gi|284802244|ref|YP 003414109.1	gi|662858600|ref|NC_013766.2|:c1987928-1986270
hypothetical protein LM5578 2003	gi|284802247|ref|YP 003414112.1	gi|662858600|ref|NC_013766.2|:c1993572-1992220
hypothetical protein LM5578 2006	gi|284802250|ref|YP 003414115.1	gi|662858600|ref|NC_013766.2|:c1998591-1995031
hypothetical protein LM5578 2007	gi|284802251|ref|YP 003414116.1	gi|662858600|ref|NC_013766.2|:c1999303-1998614
fatty acid biosynthesis transcriptional regulator	gi|284802256|ref|YP 003414121.1	gi|662858600|ref|NC_013766.2|:c2003121-2002552
hypothetical protein LM5578 2015	gi|284802259|ref|YP 003414124.1	gi|662858600|ref|NC_013766.2|:c2007031-2006369
hypothetical protein LM5578 2016	gi|284802260|ref|YP 003414125.1	gi|662858600|ref|NC_013766.2|:c2008729-2007071
hypothetical protein LM5578 2019	gi|284802263|ref|YP 003414128.1	gi|662858600|ref|NC_013766.2|:c2010336-2009692
ribulose-phosphate 3-epimerase	gi|284802264|ref|YP	gi|662858600|ref|NC_013766.2|:c2011056-2010400

	003414129.1	
ribosome-associated GTPase	gi|284802265|ref|YP 003414130.1	gi|662858600|ref|NC_013766.2|:c2011934-2011059
hypothetical protein LM5578 2022	gi|284802266|ref|YP 003414131.1	gi|662858600|ref|NC_013766.2|:c2013920-2011953
hypothetical protein LM5578 2024	gi|284802268|ref|YP 003414133.1	gi|662858600|ref|NC_013766.2|:c2016032-2014698
hypothetical protein LM5578 2029	gi|284802273|ref|YP 003414138.1	gi|662858600|ref|NC_013766.2|:c2021560-2020940
hypothetical protein LM5578 2031	gi|284802275|ref|YP 003414140.1	gi|662858600|ref|NC_013766.2|:2022608-2024320
short chain dehydrogenase	gi|284802276|ref|YP 003414141.1	gi|662858600|ref|NC_013766.2|:2024408-2025007
orotate phosphoribosyltransferase	gi|284802277|ref|YP 003414142.1	gi|662858600|ref|NC_013766.2|:c2025677-2025048
hypothetical protein LM5578 2034	gi|284802278|ref|YP 003414143.1	gi|662858600|ref|NC_013766.2|:c2026375-2025674
dihydroorotate dehydrogenase 1B	gi|284802279|ref|YP 003414144.1	gi|662858600|ref|NC_013766.2|:c2027286-2026372
hypothetical protein LM5578 2036	gi|284802280|ref|YP 003414145.1	gi|662858600|ref|NC_013766.2|:c2028047-2027283
carbamoyl phosphate synthase large subunit	gi|284802281|ref|YP 003414146.1	gi|662858600|ref|NC_013766.2|:c2031282-2028070
carbamoyl phosphate synthase small subunit	gi|284802282|ref|YP 003414147.1	gi|662858600|ref|NC_013766.2|:c2032366-2031275
hypothetical protein LM5578 2041	gi|284802285|ref|YP 003414150.1	gi|662858600|ref|NC_013766.2|:c2035912-2034626
pyrimidine regulatory protein PyrR	gi|284802286|ref|YP 003414151.1	gi|662858600|ref|NC_013766.2|:c2036592-2036041

hypothetical protein LM5578 2045	gi|284802289|ref|YP 003414154.1	gi|662858600|ref|NC_013766.2|:c2038896-2037985
hypothetical protein LM5578 2049	gi|284802293|ref|YP 003414158.1	gi|662858600|ref|NC_013766.2|:c2043217-2042285
hypothetical protein LM5578 2050	gi|284802294|ref|YP 003414159.1	gi|662858600|ref|NC_013766.2|:c2044056-2043214
hypothetical protein LM5578 2051	gi|284802295|ref|YP 003414160.1	gi|662858600|ref|NC_013766.2|:c2044782-2044060
hypothetical protein LM5578 2053	gi|284802297|ref|YP 003414162.1	gi|662858600|ref|NC_013766.2|:c2046884-2045394
hypothetical protein LM5578 2057	gi|284802301|ref|YP 003414166.1	gi|662858600|ref|NC_013766.2|:c2050725-2049901
purine nucleoside phosphorylase	gi|284802302|ref|YP 003414167.1	gi|662858600|ref|NC_013766.2|:c2051484-2050783
hypothetical protein LM5578 2060	gi|284802304|ref|YP 003414169.1	gi|662858600|ref|NC_013766.2|:c2052697-2051792
methionine sulfoxide reductase A	gi|284802306|ref|YP 003414171.1	gi|662858600|ref|NC_013766.2|:c2053757-2053224
hypothetical protein LM5578 2064	gi|284802308|ref|YP 003414173.1	gi|662858600|ref|NC_013766.2|:c2055208-2054423
hypothetical protein LM5578 2066	gi|284802310|ref|YP 003414175.1	gi|662858600|ref|NC_013766.2|:2056241-2056873
hypothetical protein LM5578 2067	gi|284802311|ref|YP 003414176.1	gi|662858600|ref|NC_013766.2|:2056995-2057612
hypothetical protein LM5578 2068	gi|284802312|ref|YP 003414177.1	gi|662858600|ref|NC_013766.2|:2057625-2058437
hypothetical protein LM5578 2071	gi|284802315|ref|YP 003414180.1	gi|662858600|ref|NC_013766.2|:c2062509-2061526
hypothetical protein LM5578 2072	gi|284802316|ref|YP 003414181.1	gi|662858600|ref|NC_013766.2|:c2063226-2062603

hypothetical protein LM5578 2073	gi|284802317|ref|YP 003414182.1	gi|662858600|ref|NC_013766.2|:c2064877-2063366
thymidylate synthase	gi|284802320|ref|YP 003414185.1	gi|662858600|ref|NC_013766.2|:c2067316-2066372
hypothetical protein LM5578 2077	gi|284802321|ref|YP 003414186.1	gi|662858600|ref|NC_013766.2|:c2069221-2067329
hypothetical protein LM5578 2082	gi|284802326|ref|YP 003414191.1	gi|662858600|ref|NC_013766.2|:c2073587-2072715
hypothetical protein LM5578 2084	gi|284802328|ref|YP 003414193.1	gi|662858600|ref|NC_013766.2|:c2075116-2074058
hypothetical protein LM5578 2085	gi|284802329|ref|YP 003414194.1	gi|662858600|ref|NC_013766.2|:c2076615-2075308
xanthine phosphoribosyltransferase	gi|284802330|ref|YP 003414195.1	gi|662858600|ref|NC_013766.2|:c2077200-2076622
hypothetical protein LM5578 2088	gi|284802332|ref|YP 003414197.1	gi|662858600|ref|NC_013766.2|:c2080104-2078956
hypothetical protein LM5578 2090	gi|284802334|ref|YP 003414199.1	gi|662858600|ref|NC_013766.2|:c2081703-2081158
Holliday junction-specific endonuclease	gi|284802336|ref|YP 003414201.1	gi|662858600|ref|NC_013766.2|:2082312-2082917
endonuclease III (DNA repair)	gi|284802339|ref|YP 003414204.1	gi|662858600|ref|NC_013766.2|:c2086618-2085959
hypothetical protein LM5578 2096	gi|284802340|ref|YP 003414205.1	gi|662858600|ref|NC_013766.2|:c2087348-2086629
asparaginyl-tRNA synthetase	gi|284802341|ref|YP 003414206.1	gi|662858600|ref|NC_013766.2|:c2088789-2087497
aspartate aminotransferase	gi|284802342|ref|YP 003414207.1	gi|662858600|ref|NC_013766.2|:c2089984-2088803
hypothetical protein LM5578 2099	gi|284802343|ref|YP 003414208.1	gi|662858600|ref|NC_013766.2|:c2090591-2090007

bifunctional ATP-dependent DNA helicase/DNA polymerase III subunit epsilon	gi|284802344|ref|YP 003414209.1	gi|662858600|ref|NC_013766.2|:c2093422-2090636
hypothetical protein LM5578 2102	gi|284802346|ref|YP 003414211.1	gi|662858600|ref|NC_013766.2|:c2094763-2093906
3-methyl-2-oxobutanoate hydroxymethyltransferase	gi|284802347|ref|YP 003414212.1	gi|662858600|ref|NC_013766.2|:c2095600-2094767
tRNA CCA-pyrophosphorylase	gi|284802350|ref|YP 003414215.1	gi|662858600|ref|NC_013766.2|:c2098536-2097355
dihydrodipicolinate reductase	gi|284802352|ref|YP 003414217.1	gi|662858600|ref|NC_013766.2|:c2099762-2098971
hypothetical protein LM5578 2110	gi|284802354|ref|YP 003414219.1	gi|662858600|ref|NC_013766.2|:2100234-2101100
hypothetical protein LM5578 2111	gi|284802355|ref|YP 003414220.1	gi|662858600|ref|NC_013766.2|:c2102196-2101090
hypothetical protein LM5578 2112	gi|284802356|ref|YP 003414221.1	gi|662858600|ref|NC_013766.2|:2102516-2103652
hypothetical protein LM5578 2113	gi|284802357|ref|YP 003414222.1	gi|662858600|ref|NC_013766.2|:2103678-2104805
malate dehydrogenase	gi|284802360|ref|YP 003414225.1	gi|662858600|ref|NC_013766.2|:2106619-2108262
hypothetical protein LM5578 2117	gi|284802361|ref|YP 003414226.1	gi|662858600|ref|NC_013766.2|:2108265-2109272
hypothetical protein LM5578 2119	gi|284802363|ref|YP 003414228.1	gi|662858600|ref|NC_013766.2|:c2113097-2111832
hypothetical protein LM5578 2120	gi|284802364|ref|YP 003414229.1	gi|662858600|ref|NC_013766.2|:c2113894-2113211
hypothetical protein LM5578 2121	gi|284802365|ref|YP 003414230.1	gi|662858600|ref|NC_013766.2|:c2114576-2113980

hypothetical protein LM5578 2122	gi|284802366|ref|YP 003414231.1	gi|662858600|ref|NC_013766.2|:c2115209-2114664
hypothetical protein LM5578 2123	gi|284802367|ref|YP 003414232.1	gi|662858600|ref|NC_013766.2|:c2116497-2115244
prephenate dehydrogenase	gi|284802369|ref|YP 003414234.1	gi|662858600|ref|NC_013766.2|:c2119031-2117928
histidinol-phosphate aminotransferase	gi|284802370|ref|YP 003414235.1	gi|662858600|ref|NC_013766.2|:c2120134-2119052
chorismate synthase	gi|284802373|ref|YP 003414238.1	gi|662858600|ref|NC_013766.2|:c2122771-2121605
ubiquinone/menaquinone biosynthesis methyltransferase	gi|284802376|ref|YP 003414241.1	gi|662858600|ref|NC_013766.2|:c2125126-2124413
hypothetical protein LM5578 2133	gi|284802377|ref|YP 003414242.1	gi|662858600|ref|NC_013766.2|:c2125916-2125149
GTP cyclohydrolase I	gi|284802378|ref|YP 003414243.1	gi|662858600|ref|NC_013766.2|:c2126544-2125975
hypothetical protein LM5578 2136	gi|284802380|ref|YP 003414245.1	gi|662858600|ref|NC_013766.2|:c2128447-2127467
GTP-binding protein EngA	gi|284802382|ref|YP 003414247.1	gi|662858600|ref|NC_013766.2|:c2130899-2129589
30S ribosomal protein S1	gi|284802383|ref|YP 003414248.1	gi|662858600|ref|NC_013766.2|:c2132188-2131043
hypothetical protein LM5578 2140	gi|284802384|ref|YP 003414249.1	gi|662858600|ref|NC_013766.2|:c2133221-2132547
hypothetical protein LM5578 2141	gi|284802385|ref|YP 003414250.1	gi|662858600|ref|NC_013766.2|:c2134199-2133237
hypothetical protein LM5578 2148	gi|284802392|ref|YP 003414257.1	gi|662858600|ref|NC_013766.2|:c2141523-2139733
hypothetical protein LM5578 2149	gi|284802393|ref|YP 003414258.1	gi|662858600|ref|NC_013766.2|:c2142340-2141624

hypothetical protein LM5578 2150	gi|284802394|ref|YP 003414259.1	gi|662858600|ref|NC_013766.2|:c2143257-2142523
hypothetical protein LM5578 2151	gi|284802395|ref|YP 003414260.1	gi|662858600|ref|NC_013766.2|:c2143856-2143260
segregation and condensation protein A	gi|284802396|ref|YP 003414261.1	gi|662858600|ref|NC_013766.2|:c2144602-2143853
purine nucleoside phosphorylase	gi|284802398|ref|YP 003414263.1	gi|662858600|ref|NC_013766.2|:c2146927-2146109
phosphopentomutase	gi|284802399|ref|YP 003414264.1	gi|662858600|ref|NC_013766.2|:c2148130-2146946
hypothetical protein LM5578 2156	gi|284802400|ref|YP 003414265.1	gi|662858600|ref|NC_013766.2|:c2149052-2148159
hypothetical protein LM5578 2157	gi|284802401|ref|YP 003414266.1	gi|662858600|ref|NC_013766.2|:c2149759-2149205
hypothetical protein LM5578 2160	gi|284802404|ref|YP 003414269.1	gi|662858600|ref|NC_013766.2|:c2152989-2152048
hypothetical protein LM5578 2161	gi|284802405|ref|YP 003414270.1	gi|662858600|ref|NC_013766.2|:2153154-2153978
hypothetical protein LM5578 2163	gi|284802407|ref|YP 003414272.1	gi|662858600|ref|NC_013766.2|:c2155602-2154982
hypothetical protein LM5578 2164	gi|284802408|ref|YP 003414273.1	gi|662858600|ref|NC_013766.2|:c2156417-2155602
hypothetical protein LM5578 2166	gi|284802410|ref|YP 003414275.1	gi|662858600|ref|NC_013766.2|:c2158459-2157902
hypothetical protein LM5578 2167	gi|284802411|ref|YP 003414276.1	gi|662858600|ref|NC_013766.2|:2158735-2159394
hypothetical protein LM5578 2176	gi|284802420|ref|YP 003414285.1	gi|662858600|ref|NC_013766.2|:c2166974-2165904
hypothetical protein LM5578 2178	gi|284802422|ref|YP 003414287.1	gi|662858600|ref|NC_013766.2|:c2168799-2167879

2-isopropylmalate synthase	gi|284802433|ref|YP 003414298.1	gi|662858600|ref|NC_013766.2|:2178130-2179668
3-isopropylmalate dehydrogenase	gi|284802434|ref|YP 003414299.1	gi|662858600|ref|NC_013766.2|:2179670-2180722
isopropylmalate isomerase large subunit	gi|284802435|ref|YP 003414300.1	gi|662858600|ref|NC_013766.2|:2180724-2182112
isopropylmalate isomerase small subunit	gi|284802436|ref|YP 003414301.1	gi|662858600|ref|NC_013766.2|:2182099-2182680
hypothetical protein LM5578 2200	gi|284802444|ref|YP 003414309.1	gi|662858600|ref|NC_013766.2|:c2190644-2189649
hypothetical protein LM5578 2202	gi|284802446|ref|YP 003414311.1	gi|662858600|ref|NC_013766.2|:c2192565-2191744
hypothetical protein LM5578 2203	gi|284802447|ref|YP 003414312.1	gi|662858600|ref|NC_013766.2|:c2193364-2192546
hypothetical protein LM5578 2205	gi|284802449|ref|YP 003414314.1	gi|662858600|ref|NC_013766.2|:c2194575-2193874
acetolactate synthase	gi|284802452|ref|YP 003414317.1	gi|662858600|ref|NC_013766.2|:c2198290-2196593
hypothetical protein LM5578 2209	gi|284802453|ref|YP 003414318.1	gi|662858600|ref|NC_013766.2|:c2199943-2198486
hypothetical protein LM5578 2210	gi|284802454|ref|YP 003414319.1	gi|662858600|ref|NC_013766.2|:c2200935-2199964
hypothetical protein LM5578 2211	gi|284802455|ref|YP 003414320.1	gi|662858600|ref|NC_013766.2|:c2201883-2200954
hypothetical protein LM5578 2213	gi|284802457|ref|YP 003414322.1	gi|662858600|ref|NC_013766.2|:c2205213-2203480
hypothetical protein LM5578 2214	gi|284802458|ref|YP 003414323.1	gi|662858600|ref|NC_013766.2|:c2205887-2205276
hypothetical protein LM5578 2215	gi|284802459|ref|YP 003414324.1	gi|662858600|ref|NC_013766.2|:c2207252-2205948

hypothetical protein LM5578 2219	gi|284802463|ref|YP 003414328.1	gi|662858600|ref|NC_013766.2|:c2214520-2213825
hypothetical protein LM5578 2222	gi|284802466|ref|YP 003414331.1	gi|662858600|ref|NC_013766.2|:c2219210-2218683
hypothetical protein LM5578 2223	gi|284802467|ref|YP 003414332.1	gi|662858600|ref|NC_013766.2|:c2219825-2219304
hypothetical protein LM5578 2234	gi|284802478|ref|YP 003414343.1	gi|662858600|ref|NC_013766.2|:c2229565-2228876
cell division protein FtsZ	gi|284802479|ref|YP 003414344.1	gi|662858600|ref|NC_013766.2|:c2230859-2229684
hypothetical protein LM5578 2236	gi|284802480|ref|YP 003414345.1	gi|662858600|ref|NC_013766.2|:c2232206-2230926
UDP-N-acetylmuramoyl-L-alanyl-D-glutamate synthetase	gi|284802483|ref|YP 003414348.1	gi|662858600|ref|NC_013766.2|:c2235862-2234495
hypothetical protein LM5578 2240	gi|284802484|ref|YP 003414349.1	gi|662858600|ref|NC_013766.2|:c2236977-2236003
hypothetical protein LM5578 2242	gi|284802486|ref|YP 003414351.1	gi|662858600|ref|NC_013766.2|:c2240890-2238674
S-adenosyl-methyltransferase MraW	gi|284802488|ref|YP 003414353.1	gi|662858600|ref|NC_013766.2|:c2242243-2241305
hypothetical protein LM5578 2249	gi|284802493|ref|YP 003414358.1	gi|662858600|ref|NC_013766.2|:c2248219-2247683
hypothetical protein LM5578 2251	gi|284802495|ref|YP 003414360.1	gi|662858600|ref|NC_013766.2|:2249603-2251843
hypothetical protein LM5578 2257	gi|284802501|ref|YP 003414366.1	gi|662858600|ref|NC_013766.2|:c2255936-2254893
protoheme IX farnesyltransferase	gi|284802502|ref|YP 003414367.1	gi|662858600|ref|NC_013766.2|:c2257000-2256095
hypothetical protein LM5578 2260	gi|284802504|ref|YP 003414369.1	gi|662858600|ref|NC_013766.2|:2258262-2259005

hypothetical protein LM5578 2261	gi|284802505|ref|YP 003414370.1	gi|662858600|ref|NC_013766.2|:c2259652-2259011
hypothetical protein LM5578 2262	gi|284802506|ref|YP 003414371.1	gi|662858600|ref|NC_013766.2|:c2260288-2259671
hypothetical protein LM5578 2263	gi|284802507|ref|YP 003414372.1	gi|662858600|ref|NC_013766.2|:c2261925-2260300
chaperonin GroEL	gi|284802513|ref|YP 003414378.1	gi|662858600|ref|NC_013766.2|:c2266503-2264875
hypothetical protein LM5578 2272	gi|284802516|ref|YP 003414381.1	gi|662858600|ref|NC_013766.2|:2267059-2267745
hypothetical protein LM5578 2275	gi|284802519|ref|YP 003414384.1	gi|662858600|ref|NC_013766.2|:2268935-2270887
hypothetical protein LM5578 2276	gi|284802520|ref|YP 003414385.1	gi|662858600|ref|NC_013766.2|:2270911-2271870
O-sialoglycoprotein endopeptidase	gi|284802521|ref|YP 003414386.1	gi|662858600|ref|NC_013766.2|:c2273436-2272414
hypothetical protein LM5578 2279	gi|284802523|ref|YP 003414388.1	gi|662858600|ref|NC_013766.2|:c2274577-2273885
hypothetical protein LM5578 2281	gi|284802525|ref|YP 003414390.1	gi|662858600|ref|NC_013766.2|:c2276282-2275140
hypothetical protein LM5578 2289	gi|284802533|ref|YP 003414398.1	gi|662858600|ref|NC_013766.2|:c2284532-2283201
argininosuccinate lyase	gi|284802537|ref|YP 003414402.1	gi|662858600|ref|NC_013766.2|:2287719-2289089
glycine betaine transporter BetL	gi|284802538|ref|YP 003414403.1	gi|662858600|ref|NC_013766.2|:2289257-2290780
hypothetical protein LM5578 2297	gi|284802541|ref|YP 003414406.1	gi|662858600|ref|NC_013766.2|:c2292609-2291677
hypothetical protein LM5578 2301	gi|284802545|ref|YP 003414410.1	gi|662858600|ref|NC_013766.2|:c2296766-2294760

hypothetical protein LM5578 2307	gi|284802551|ref|YP 003414416.1	gi|662858600|ref|NC_013766.2|:2301533-2303527
hypothetical protein LM5578 2311	gi|284802555|ref|YP 003414420.1	gi|662858600|ref|NC_013766.2|:2305619-2306752
hypothetical protein LM5578 2312	gi|284802556|ref|YP 003414421.1	gi|662858600|ref|NC_013766.2|:2306794-2307591
putative heme peroxidase	gi|284802560|ref|YP 003414425.1	gi|662858600|ref|NC_013766.2|:2309882-2310637
hypothetical protein LM5578 2320	gi|284802564|ref|YP 003414429.1	gi|662858600|ref|NC_013766.2|:2314451-2314912
hypothetical protein LM5578 2321	gi|284802565|ref|YP 003414430.1	gi|662858600|ref|NC_013766.2|:c2316299-2314947
hypothetical protein LM5578 2322	gi|284802566|ref|YP 003414431.1	gi|662858600|ref|NC_013766.2|:c2317910-2316549
hypothetical protein LM5578 2323	gi|284802567|ref|YP 003414432.1	gi|662858600|ref|NC_013766.2|:c2318728-2317907
hypothetical protein LM5578 2326	gi|284802570|ref|YP 003414435.1	gi|662858600|ref|NC_013766.2|:c2322892-2322041
hypothetical protein LM5578 2328	gi|284802572|ref|YP 003414437.1	gi|662858600|ref|NC_013766.2|:c2325546-2324287
hypothetical protein LM5578 2329	gi|284802573|ref|YP 003414438.1	gi|662858600|ref|NC_013766.2|:c2327535-2325760
hypothetical protein LM5578 2334	gi|284802578|ref|YP 003414443.1	gi|662858600|ref|NC_013766.2|:c2333928-2333239
hypothetical protein LM5578 2342	gi|284802586|ref|YP 003414451.1	gi|662858600|ref|NC_013766.2|:2340507-2341409
hypothetical protein LM5578 2343	gi|284802587|ref|YP 003414452.1	gi|662858600|ref|NC_013766.2|:2341417-2342637
hypothetical protein LM5578 2345	gi|284802589|ref|YP 003414454.1	gi|662858600|ref|NC_013766.2|:c2344380-2343715

hypothetical protein LM5578 2346	gi|284802590|ref|YP 003414455.1	gi|662858600|ref|NC_013766.2|:2344594-2346336
hypothetical protein LM5578 2347	gi|284802591|ref|YP 003414456.1	gi|662858600|ref|NC_013766.2|:c2347109-2346375
hypothetical protein LM5578 2348	gi|284802592|ref|YP 003414457.1	gi|662858600|ref|NC_013766.2|:c2348240-2347224
hypothetical protein LM5578 2349	gi|284802593|ref|YP 003414458.1	gi|662858600|ref|NC_013766.2|:c2349163-2348309
hypothetical protein LM5578 2350	gi|284802594|ref|YP 003414459.1	gi|662858600|ref|NC_013766.2|:2349269-2350276
ribonucleotide-diphosphate reductase subunit beta	gi|284802601|ref|YP 003414466.1	gi|662858600|ref|NC_013766.2|:c2353967-2352918
ribonucleotide-diphosphate reductase subunit alpha	gi|284802602|ref|YP 003414467.1	gi|662858600|ref|NC_013766.2|:c2356314-2354023
hypothetical protein LM5578 2367	gi|284802611|ref|YP 003414476.1	gi|662858600|ref|NC_013766.2|:c2365108-2364230
hypothetical protein LM5578 2368	gi|284802612|ref|YP 003414477.1	gi|662858600|ref|NC_013766.2|:c2365873-2365178
hypothetical protein LM5578 2369	gi|284802613|ref|YP 003414478.1	gi|662858600|ref|NC_013766.2|:c2366797-2366126
hypothetical protein LM5578 2370	gi|284802614|ref|YP 003414479.1	gi|662858600|ref|NC_013766.2|:c2367480-2366863
hypothetical protein LM5578 2374	gi|284802618|ref|YP 003414483.1	gi|662858600|ref|NC_013766.2|:c2370404-2369181
hypothetical protein LM5578 2376	gi|284802620|ref|YP 003414485.1	gi|662858600|ref|NC_013766.2|:c2373581-2372214
hypothetical protein LM5578 2377	gi|284802621|ref|YP 003414486.1	gi|662858600|ref|NC_013766.2|:c2374760-2373687
hypothetical protein LM5578 2384	gi|284802628|ref|YP 003414493.1	gi|662858600|ref|NC_013766.2|:c2386860-2386120

hypothetical protein LM5578 2385	gi|284802629|ref|YP 003414494.1	gi|662858600|ref|NC_013766.2|:c2387643-2386864
hypothetical protein LM5578 2386	gi|284802630|ref|YP 003414495.1	gi|662858600|ref|NC_013766.2|:c2388613-2387624
hypothetical protein LM5578 2387	gi|284802631|ref|YP 003414496.1	gi|662858600|ref|NC_013766.2|:c2389475-2388603
hypothetical protein LM5578 2388	gi|284802632|ref|YP 003414497.1	gi|662858600|ref|NC_013766.2|:c2391271-2389562
hypothetical protein LM5578 2389	gi|284802633|ref|YP 003414498.1	gi|662858600|ref|NC_013766.2|:c2391909-2391286
hypothetical protein LM5578 2391	gi|284802635|ref|YP 003414500.1	gi|662858600|ref|NC_013766.2|:c2394484-2392679
hypothetical protein LM5578 2392	gi|284802636|ref|YP 003414501.1	gi|662858600|ref|NC_013766.2|:c2395672-2394557
adaptor protein	gi|284802637|ref|YP 003414502.1	gi|662858600|ref|NC_013766.2|:c2396451-2395798
hypothetical protein LM5578 2396	gi|284802640|ref|YP 003414505.1	gi|662858600|ref|NC_013766.2|:c2399438-2398362
hypothetical protein LM5578 2397	gi|284802641|ref|YP 003414506.1	gi|662858600|ref|NC_013766.2|:c2400490-2399456
hypothetical protein LM5578 2398	gi|284802642|ref|YP 003414507.1	gi|662858600|ref|NC_013766.2|:c2401419-2400490
hypothetical protein LM5578 2399	gi|284802643|ref|YP 003414508.1	gi|662858600|ref|NC_013766.2|:c2403374-2401698
tryptophanyl-tRNA synthetase	gi|284802645|ref|YP 003414510.1	gi|662858600|ref|NC_013766.2|:2404676-2405671
hypothetical protein LM5578 2406	gi|284802650|ref|YP 003414515.1	gi|662858600|ref|NC_013766.2|:c2410332-2409205
hypothetical protein LM5578 2408	gi|284802652|ref|YP 003414517.1	gi|662858600|ref|NC_013766.2|:c2411622-2410933

hypothetical protein LM5578 2409	gi|284802653|ref|YP 003414518.1	gi|662858600|ref|NC_013766.2|:c2414313-2411713
hypothetical protein LM5578 2410	gi|284802654|ref|YP 003414519.1	gi|662858600|ref|NC_013766.2|:c2415139-2414462
hypothetical protein LM5578 2411	gi|284802655|ref|YP 003414520.1	gi|662858600|ref|NC_013766.2|:c2416021-2415251
hypothetical protein LM5578 2412	gi|284802656|ref|YP 003414521.1	gi|662858600|ref|NC_013766.2|:c2416870-2416085
ferrochelatase	gi|284802658|ref|YP 003414523.1	gi|662858600|ref|NC_013766.2|:c2418421-2417492
uroporphyrinogen decarboxylase	gi|284802659|ref|YP 003414524.1	gi|662858600|ref|NC_013766.2|:c2419479-2418418
hypothetical protein LM5578 2416	gi|284802660|ref|YP 003414525.1	gi|662858600|ref|NC_013766.2|:2419607-2420179
hypothetical protein LM5578 2417	gi|284802661|ref|YP 003414526.1	gi|662858600|ref|NC_013766.2|:c2421441-2420218
hypothetical protein LM5578 2421	gi|284802665|ref|YP 003414530.1	gi|662858600|ref|NC_013766.2|:2423335-2423874
3'-5' exoribonuclease YhaM	gi|284802667|ref|YP 003414532.1	gi|662858600|ref|NC_013766.2|:c2425899-2424913
hypothetical protein LM5578 2424	gi|284802668|ref|YP 003414533.1	gi|662858600|ref|NC_013766.2|:c2428622-2425899
hypothetical protein LM5578 2425	gi|284802669|ref|YP 003414534.1	gi|662858600|ref|NC_013766.2|:c2429854-2428619
fumarate hydratase	gi|284802672|ref|YP 003414537.1	gi|662858600|ref|NC_013766.2|:c2433036-2431669
hypothetical protein LM5578 2430	gi|284802674|ref|YP 003414539.1	gi|662858600|ref|NC_013766.2|:c2434802-2433897
hypothetical protein LM5578 2434	gi|284802678|ref|YP 003414543.1	gi|662858600|ref|NC_013766.2|:2438064-2438933

hypothetical protein LM5578 2435	gi|284802679|ref|YP 003414544.1	gi|662858600|ref|NC_013766.2|:2439189-2440493
hypothetical protein LM5578 2436	gi|284802680|ref|YP 003414545.1	gi|662858600|ref|NC_013766.2|:c2441411-2440533
hypothetical protein LM5578 2437	gi|284802681|ref|YP 003414546.1	gi|662858600|ref|NC_013766.2|:2441712-2442566
shikimate 5-dehydrogenase	gi|284802683|ref|YP 003414548.1	gi|662858600|ref|NC_013766.2|:2444618-2445487
hypothetical protein LM5578 2441	gi|284802685|ref|YP 003414550.1	gi|662858600|ref|NC_013766.2|:2446888-2448078
hypothetical protein LM5578 2446	gi|284802690|ref|YP 003414555.1	gi|662858600|ref|NC_013766.2|:2451160-2451726
hypothetical protein LM5578 2447	gi|284802691|ref|YP 003414556.1	gi|662858600|ref|NC_013766.2|:2451737-2452612
hypothetical protein LM5578 2449	gi|284802693|ref|YP 003414558.1	gi|662858600|ref|NC_013766.2|:c2453675-2453019
hypothetical protein LM5578 2450	gi|284802694|ref|YP 003414559.1	gi|662858600|ref|NC_013766.2|:c2454556-2453672
hypothetical protein LM5578 2451	gi|284802695|ref|YP 003414560.1	gi|662858600|ref|NC_013766.2|:2454943-2455563
hypothetical protein LM5578 2452	gi|284802696|ref|YP 003414561.1	gi|662858600|ref|NC_013766.2|:2455577-2456575
hypothetical protein LM5578 2456	gi|284802700|ref|YP 003414565.1	gi|662858600|ref|NC_013766.2|:c2460842-2460213
hypothetical protein LM5578 2457	gi|284802701|ref|YP 003414566.1	gi|662858600|ref|NC_013766.2|:2461015-2462307
hypothetical protein LM5578 2462	gi|284802706|ref|YP 003414571.1	gi|662858600|ref|NC_013766.2|:c2465308-2464433
hypothetical protein LM5578 2464	gi|284802708|ref|YP 003414573.1	gi|662858600|ref|NC_013766.2|:c2466513-2465761

hypothetical protein LM5578 2465	gi|284802709|ref|YP 003414574.1	gi|662858600|ref|NC_013766.2|:2466665-2467477
hypothetical protein LM5578 2466	gi|284802710|ref|YP 003414575.1	gi|662858600|ref|NC_013766.2|:c2468457-2467555
hypothetical protein LM5578 2468	gi|284802712|ref|YP 003414577.1	gi|662858600|ref|NC_013766.2|:c2469772-2468924
hypothetical protein LM5578 2469	gi|284802713|ref|YP 003414578.1	gi|662858600|ref|NC_013766.2|:c2473480-2469773
hypothetical protein LM5578 2470	gi|284802714|ref|YP 003414579.1	gi|662858600|ref|NC_013766.2|:c2476973-2473482
hypothetical protein LM5578 2533	gi|284802776|ref|YP 003414641.1	gi|662858600|ref|NC_013766.2|:c2520168-2518270
fructose-1-phosphate kinase	gi|284802777|ref|YP 003414642.1	gi|662858600|ref|NC_013766.2|:c2521093-2520170
hypothetical protein LM5578 2535	gi|284802778|ref|YP 003414643.1	gi|662858600|ref|NC_013766.2|:c2521842-2521090
hypothetical protein LM5578 2539	gi|284802782|ref|YP 003414647.1	gi|662858600|ref|NC_013766.2|:c2525922-2524804
hypothetical protein LM5578 2543	gi|284802786|ref|YP 003414651.1	gi|662858600|ref|NC_013766.2|:c2529403-2528408
hypothetical protein LM5578 2545	gi|284802788|ref|YP 003414653.1	gi|662858600|ref|NC_013766.2|:c2530886-2530176
hypothetical protein LM5578 2547	gi|284802790|ref|YP 003414655.1	gi|662858600|ref|NC_013766.2|:c2532444-2531635
hypothetical protein LM5578 2548	gi|284802791|ref|YP 003414656.1	gi|662858600|ref|NC_013766.2|:c2532996-2532460
hypothetical protein LM5578 2549	gi|284802792|ref|YP 003414657.1	gi|662858600|ref|NC_013766.2|:c2533557-2533012
hypothetical protein LM5578 2551	gi|284802794|ref|YP 003414659.1	gi|662858600|ref|NC_013766.2|:c2536679-2534727

hypothetical protein LM5578 2553	gi|284802796|ref|YP 003414661.1	gi|662858600|ref|NC_013766.2|:c2539646-2538405
hypothetical protein LM5578 2555	gi|284802798|ref|YP 003414663.1	gi|662858600|ref|NC_013766.2|:c2540458-2539847
hypothetical protein LM5578 2556	gi|284802799|ref|YP 003414664.1	gi|662858600|ref|NC_013766.2|:c2541178-2540648
hypothetical protein LM5578 2557	gi|284802800|ref|YP 003414665.1	gi|662858600|ref|NC_013766.2|:c2541926-2541195
hypothetical protein LM5578 2558	gi|284802801|ref|YP 003414666.1	gi|662858600|ref|NC_013766.2|:c2542815-2541970
hypothetical protein LM5578 2565	gi|284802808|ref|YP 003414673.1	gi|662858600|ref|NC_013766.2|:c2559051-2558293
glucose-6-phosphate isomerase	gi|284802809|ref|YP 003414674.1	gi|662858600|ref|NC_013766.2|:c2560508-2559156
hypothetical protein LM5578 2569	gi|284802812|ref|YP 003414677.1	gi|662858600|ref|NC_013766.2|:2561672-2562838
hypothetical protein LM5578 2570	gi|284802813|ref|YP 003414678.1	gi|662858600|ref|NC_013766.2|:2562977-2564080
hypothetical protein LM5578 2571	gi|284802814|ref|YP 003414679.1	gi|662858600|ref|NC_013766.2|:2564080-2564760
aspartate kinase	gi|284802816|ref|YP 003414681.1	gi|662858600|ref|NC_013766.2|:2565362-2566723
hypothetical protein LM5578 2575	gi|284802818|ref|YP 003414683.1	gi|662858600|ref|NC_013766.2|:c2567831-2567247
putative monovalent cation/H+ antiporter subunit D	gi|284802823|ref|YP 003414688.1	gi|662858600|ref|NC_013766.2|:2572774-2574261
putative monovalent cation/H+ antiporter subunit G	gi|284802826|ref|YP 003414691.1	gi|662858600|ref|NC_013766.2|:2575015-2575608
hypothetical protein LM5578 2586	gi|284802829|ref|YP 003414694.1	gi|662858600|ref|NC_013766.2|:2576692-2577954

hypothetical protein LM5578 2588	gi|284802831|ref|YP 003414696.1	gi|662858600|ref|NC_013766.2|:c2580105-2578894
hypothetical protein LM5578 2589	gi|284802832|ref|YP 003414697.1	gi|662858600|ref|NC_013766.2|:2580555-2581550
hypothetical protein LM5578 2590	gi|284802833|ref|YP 003414698.1	gi|662858600|ref|NC_013766.2|:2581638-2582267
hypothetical protein LM5578 2598	gi|284802841|ref|YP 003414706.1	gi|662858600|ref|NC_013766.2|:c2589141-2587831
hypothetical protein LM5578 2600	gi|284802843|ref|YP 003414708.1	gi|662858600|ref|NC_013766.2|:c2590436-2589669
hypothetical protein LM5578 2602	gi|284802845|ref|YP 003414710.1	gi|662858600|ref|NC_013766.2|:c2592116-2590728
hypothetical protein LM5578 2605	gi|284802848|ref|YP 003414713.1	gi|662858600|ref|NC_013766.2|:c2594333-2593491
hypothetical protein LM5578 2610	gi|284802853|ref|YP 003414718.1	gi|662858600|ref|NC_013766.2|:c2599897-2599112
hypothetical protein LM5578 2611	gi|284802854|ref|YP 003414719.1	gi|662858600|ref|NC_013766.2|:2600237-2601313
hypothetical protein LM5578 2612	gi|284802855|ref|YP 003414720.1	gi|662858600|ref|NC_013766.2|:c2602185-2601355
hypothetical protein LM5578 2613	gi|284802856|ref|YP 003414721.1	gi|662858600|ref|NC_013766.2|:c2602923-2602249
hypothetical protein LM5578 2618	gi|284802861|ref|YP 003414726.1	gi|662858600|ref|NC_013766.2|:c2607303-2606428
hypothetical protein LM5578 2625	gi|284802868|ref|YP 003414733.1	gi|662858600|ref|NC_013766.2|:c2613153-2612143
hypothetical protein LM5578 2626	gi|284802869|ref|YP 003414734.1	gi|662858600|ref|NC_013766.2|:c2614124-2613153
hypothetical protein LM5578 2636	gi|284802879|ref|YP 003414744.1	gi|662858600|ref|NC_013766.2|:c2621175-2620279

hypothetical protein LM5578 2641	gi|284802884|ref|YP 003414749.1	gi|662858600|ref|NC_013766.2|:c2628225-2627281
hypothetical protein LM5578 2646	gi|284802889|ref|YP 003414754.1	gi|662858600|ref|NC_013766.2|:c2636549-2635803
hypothetical protein LM5578 2648	gi|284802891|ref|YP 003414756.1	gi|662858600|ref|NC_013766.2|:c2637884-2637141
hypothetical protein LM5578 2649	gi|284802892|ref|YP 003414757.1	gi|662858600|ref|NC_013766.2|:c2638786-2638067
hypothetical protein LM5578 2652	gi|284802895|ref|YP 003414760.1	gi|662858600|ref|NC_013766.2|:c2642912-2642157
phosphoglycerate kinase	gi|284802896|ref|YP 003414761.1	gi|662858600|ref|NC_013766.2|:c2644148-2642958
glyceraldehyde-3-phosphate dehydrogenase	gi|284802897|ref|YP 003414762.1	gi|662858600|ref|NC_013766.2|:c2645293-2644283
hypothetical protein LM5578 2655	gi|284802898|ref|YP 003414763.1	gi|662858600|ref|NC_013766.2|:c2646373-2645327
hypothetical protein LM5578 2657	gi|284802900|ref|YP 003414765.1	gi|662858600|ref|NC_013766.2|:c2649244-2648318
hypothetical protein LM5578 2659	gi|284802902|ref|YP 003414767.1	gi|662858600|ref|NC_013766.2|:c2652175-2651564
hypothetical protein LM5578 2662	gi|284802905|ref|YP 003414770.1	gi|662858600|ref|NC_013766.2|:c2654830-2653397
ATP-dependent Clp protease proteolytic subunit	gi|284802906|ref|YP 003414771.1	gi|662858600|ref|NC_013766.2|:2654987-2655583
hypothetical protein LM5578 2667	gi|284802910|ref|YP 003414775.1	gi|662858600|ref|NC_013766.2|:c2660471-2659500
hypothetical protein LM5578 2668	gi|284802911|ref|YP 003414776.1	gi|662858600|ref|NC_013766.2|:c2661447-2660479
hypothetical protein LM5578 2669	gi|284802912|ref|YP 003414777.1	gi|662858600|ref|NC_013766.2|:c2662345-2661449

hypothetical protein LM5578 2671	gi|284802914|ref|YP 003414779.1	gi|662858600|ref|NC_013766.2|:c2665265-2664204
UDP-glucose 4-epimerase	gi|284802915|ref|YP 003414780.1	gi|662858600|ref|NC_013766.2|:c2666265-2665282
thioredoxin reductase	gi|284802916|ref|YP 003414781.1	gi|662858600|ref|NC_013766.2|:c2667343-2666384
hypothetical protein LM5578 2674	gi|284802917|ref|YP 003414782.1	gi|662858600|ref|NC_013766.2|:c2668915-2667422
pyrophosphatase PpaX	gi|284802919|ref|YP 003414784.1	gi|662858600|ref|NC_013766.2|:c2670137-2669484
prolipoprotein diacylglyceryl transferase	gi|284802920|ref|YP 003414785.1	gi|662858600|ref|NC_013766.2|:c2671014-2670181
HPr kinase/phosphorylase	gi|284802921|ref|YP 003414786.1	gi|662858600|ref|NC_013766.2|:c2672038-2671100
hypothetical protein LM5578 2681	gi|284802924|ref|YP 003414789.1	gi|662858600|ref|NC_013766.2|:c2674022-2672811
excinuclease ABC (subunit A)	gi|284802926|ref|YP 003414791.1	gi|662858600|ref|NC_013766.2|:c2678431-2675561
excinuclease ABC subunit B	gi|284802927|ref|YP 003414792.1	gi|662858600|ref|NC_013766.2|:c2680415-2678439
hypothetical protein LM5578 2686	gi|284802929|ref|YP 003414794.1	gi|662858600|ref|NC_013766.2|:c2681573-2680926
hypothetical protein LM5578 2689	gi|284802932|ref|YP 003414797.1	gi|662858600|ref|NC_013766.2|:c2683319-2682660
hypothetical protein LM5578 2690	gi|284802933|ref|YP 003414798.1	gi|662858600|ref|NC_013766.2|:c2684111-2683332
hypothetical protein LM5578 2693	gi|284802936|ref|YP 003414801.1	gi|662858600|ref|NC_013766.2|:c2686800-2685850
hypothetical protein LM5578 2694	gi|284802937|ref|YP 003414802.1	gi|662858600|ref|NC_013766.2|:c2687775-2686867

two-component sensor histidine kinase	gi|284802938|ref|YP 003414803.1	gi|662858600|ref|NC_013766.2|:c2689806-2688031
two-component response phosphate regulator	gi|284802939|ref|YP 003414804.1	gi|662858600|ref|NC_013766.2|:c2690516-2689806
hypothetical protein LM5578 2697	gi|284802940|ref|YP 003414805.1	gi|662858600|ref|NC_013766.2|:c2691784-2690666
hypothetical protein LM5578 2698	gi|284802941|ref|YP 003414806.1	gi|662858600|ref|NC_013766.2|:c2693317-2691869
hypothetical protein LM5578 2701	gi|284802944|ref|YP 003414809.1	gi|662858600|ref|NC_013766.2|:c2697035-2696151
hypothetical protein LM5578 2702	gi|284802945|ref|YP 003414810.1	gi|662858600|ref|NC_013766.2|:c2697711-2697025
hypothetical protein LM5578 2703	gi|284802946|ref|YP 003414811.1	gi|662858600|ref|NC_013766.2|:c2699078-2698215
hypothetical protein LM5578 2706	gi|284802949|ref|YP 003414814.1	gi|662858600|ref|NC_013766.2|:c2703577-2703014
hypothetical protein LM5578 2707	gi|284802950|ref|YP 003414815.1	gi|662858600|ref|NC_013766.2|:c2704428-2703772
hypothetical protein LM5578 2708	gi|284802951|ref|YP 003414816.1	gi|662858600|ref|NC_013766.2|:c2705740-2704421
hypothetical protein LM5578 2709	gi|284802952|ref|YP 003414817.1	gi|662858600|ref|NC_013766.2|:c2706786-2705935
hypothetical protein LM5578 2710	gi|284802953|ref|YP 003414818.1	gi|662858600|ref|NC_013766.2|:c2707493-2706807
hypothetical protein LM5578 2713	gi|284802956|ref|YP 003414821.1	gi|662858600|ref|NC_013766.2|:2709236-2710294
hypothetical protein LM5578 2714	gi|284802957|ref|YP 003414822.1	gi|662858600|ref|NC_013766.2|:c2711385-2710333
hypothetical protein LM5578 2720	gi|284802963|ref|YP 003414828.1	gi|662858600|ref|NC_013766.2|:c2716670-2715675

UDP-N-acetylglucosamine 1-carboxyvinyltransferase	gi|284802964|ref|YP 003414829.1	gi|662858600|ref|NC_013766.2|:c2718125-2716833
hypothetical protein LM5578 2737	gi|284802980|ref|YP 003414845.1	gi|662858600|ref|NC_013766.2|:c2732399-2731548
threonine synthase	gi|284802984|ref|YP 003414849.1	gi|662858600|ref|NC_013766.2|:c2736093-2735038
homoserine dehydrogenase	gi|284802985|ref|YP 003414850.1	gi|662858600|ref|NC_013766.2|:c2737382-2736096
UDP-N-acetylglucosamine 1-carboxyvinyltransferase	gi|284802990|ref|YP 003414855.1	gi|662858600|ref|NC_013766.2|:c2742058-2740787
hypothetical protein LM5578 2749	gi|284802992|ref|YP 003414857.1	gi|662858600|ref|NC_013766.2|:c2744266-2743256
hypothetical protein LM5578 2750	gi|284802993|ref|YP 003414858.1	gi|662858600|ref|NC_013766.2|:c2745557-2744274
hypothetical protein LM5578 2751	gi|284802994|ref|YP 003414859.1	gi|662858600|ref|NC_013766.2|:c2746732-2745878
putative lipid kinase	gi|284802995|ref|YP 003414860.1	gi|662858600|ref|NC_013766.2|:c2747821-2746901
CTP synthetase	gi|284802997|ref|YP 003414862.1	gi|662858600|ref|NC_013766.2|:c2752533-2750935
arginyl-tRNA synthetase	gi|284802999|ref|YP 003414864.1	gi|662858600|ref|NC_013766.2|:c2755473-2753803
hypothetical protein LM5578 2758	gi|284803001|ref|YP 003414866.1	gi|662858600|ref|NC_013766.2|:c2756650-2755997
hypothetical protein LM5578 2760	gi|284803003|ref|YP 003414868.1	gi|662858600|ref|NC_013766.2|:c2758267-2756945
hypothetical protein LM5578 2766	gi|284803009|ref|YP 003414874.1	gi|662858600|ref|NC_013766.2|:c2762547-2761933
hypothetical protein LM5578 2767	gi|284803010|ref|YP 003414875.1	gi|662858600|ref|NC_013766.2|:c2763203-2762571

hypothetical protein LM5578 2769	gi|284803012|ref|YP 003414877.1	gi|662858600|ref|NC_013766.2|:c2764729-2763731
hypothetical protein LM5578 2773	gi|284803016|ref|YP 003414881.1	gi|662858600|ref|NC_013766.2|:c2771824-2770997
hypothetical protein LM5578 2779	gi|284803022|ref|YP 003414887.1	gi|662858600|ref|NC_013766.2|:c2776595-2775216
hypothetical protein LM5578 2780	gi|284803023|ref|YP 003414888.1	gi|662858600|ref|NC_013766.2|:c2777269-2776592
formate dehydrogenase accessory protein	gi|284803024|ref|YP 003414889.1	gi|662858600|ref|NC_013766.2|:c2778101-2777316
hypothetical protein LM5578 2786	gi|284803029|ref|YP 003414894.1	gi|662858600|ref|NC_013766.2|:c2785484-2784597
lysyl-tRNA synthetase	gi|284803032|ref|YP 003414897.1	gi|662858600|ref|NC_013766.2|:c2794202-2792706
hypothetical protein LM5578 2790	gi|284803033|ref|YP 003414898.1	gi|662858600|ref|NC_013766.2|:c2795312-2794317
hypothetical protein LM5578 2795	gi|284803038|ref|YP 003414903.1	gi|662858600|ref|NC_013766.2|:c2798682-2797756
pantothenate kinase	gi|284803040|ref|YP 003414905.1	gi|662858600|ref|NC_013766.2|:c2800477-2799698
hypothetical protein LM5578 2799	gi|284803042|ref|YP 003414907.1	gi|662858600|ref|NC_013766.2|:c2804960-2803014
peptidyl-tRNA hydrolase	gi|284803048|ref|YP 003414913.1	gi|662858600|ref|NC_013766.2|:c2812397-2811837
hypothetical protein LM5578 2806	gi|284803049|ref|YP 003414914.1	gi|662858600|ref|NC_013766.2|:c2813088-2812504
50S ribosomal protein L25/general stress protein Ctc	gi|284803050|ref|YP 003414915.1	gi|662858600|ref|NC_013766.2|:c2813801-2813178
L-lactate dehydrogenase	gi|284803051|ref|YP 003414916.1	gi|662858600|ref|NC_013766.2|:2814095-2815036

hypothetical protein LM5578 2809	gi|284803052|ref|YP 003414917.1	gi|662858600|ref|NC_013766.2|:2815113-2815787
phospholipase C	gi|284803056|ref|YP 003414921.1	gi|662858600|ref|NC_013766.2|:c2818097-2817228
Zinc metalloproteinase precursor	gi|284803058|ref|YP 003414923.1	gi|662858600|ref|NC_013766.2|:c2821783-2820251
listeriolysin O precursor	gi|284803059|ref|YP 003414924.1	gi|662858600|ref|NC_013766.2|:c2823703-2822114
phosphatidylinositol-specific phospholipase c	gi|284803060|ref|YP 003414925.1	gi|662858600|ref|NC_013766.2|:2823945-2824898
phosphoribosyl pyrophosphate synthetase	gi|284803062|ref|YP 003414927.1	gi|662858600|ref|NC_013766.2|:c2826881-2825925
hypothetical protein LM5578 2820	gi|284803063|ref|YP 003414928.1	gi|662858600|ref|NC_013766.2|:c2828305-2826932
hypothetical protein LM5578 2825	gi|284803068|ref|YP 003414933.1	gi|662858600|ref|NC_013766.2|:c2832736-2832059
purine operon repressor	gi|284803069|ref|YP 003414934.1	gi|662858600|ref|NC_013766.2|:c2833729-2832911
hypothetical protein LM5578 2827	gi|284803070|ref|YP 003414935.1	gi|662858600|ref|NC_013766.2|:c2834630-2833893
4-diphosphocytidyl-2-C-methyl-D-erythritol kinase	gi|284803071|ref|YP 003414936.1	gi|662858600|ref|NC_013766.2|:c2835533-2834652
dimethyladenosine transferase	gi|284803073|ref|YP 003414938.1	gi|662858600|ref|NC_013766.2|:c2836936-2836049
hypothetical protein LM5578 2831	gi|284803074|ref|YP 003414939.1	gi|662858600|ref|NC_013766.2|:c2837504-2836929
hypothetical protein LM5578 2832	gi|284803075|ref|YP 003414940.1	gi|662858600|ref|NC_013766.2|:c2838832-2837606
hypothetical protein LM5578 2833	gi|284803076|ref|YP 003414941.1	gi|662858600|ref|NC_013766.2|:c2839897-2839124

hypothetical protein LM5578 2837	gi|284803080|ref|YP 003414945.1	gi|662858600|ref|NC_013766.2|:c2848594-2847338
hypothetical protein LM5578 2838	gi|284803081|ref|YP 003414946.1	gi|662858600|ref|NC_013766.2|:c2849470-2848622
hypothetical protein LM5578 2839	gi|284803082|ref|YP 003414947.1	gi|662858600|ref|NC_013766.2|:c2850348-2849470
hypothetical protein LM5578 2842	gi|284803085|ref|YP 003414950.1	gi|662858600|ref|NC_013766.2|:c2854688-2853828
hypothetical protein LM5578 2847	gi|284803090|ref|YP 003414955.1	gi|662858600|ref|NC_013766.2|:c2861513-2860254
hypothetical protein LM5578 2848	gi|284803091|ref|YP 003414956.1	gi|662858600|ref|NC_013766.2|:c2862438-2861581
hypothetical protein LM5578 2850	gi|284803093|ref|YP 003414958.1	gi|662858600|ref|NC_013766.2|:c2863763-2862882
hypothetical protein LM5578 2852	gi|284803095|ref|YP 003414960.1	gi|662858600|ref|NC_013766.2|:c2864789-2864016
hypothetical protein LM5578 2854	gi|284803097|ref|YP 003414962.1	gi|662858600|ref|NC_013766.2|:c2866059-2865226
DNA polymerase III subunit delta'	gi|284803098|ref|YP 003414963.1	gi|662858600|ref|NC_013766.2|:c2867033-2866065
hypothetical protein LM5578 2856	gi|284803099|ref|YP 003414964.1	gi|662858600|ref|NC_013766.2|:2867274-2868104
hypothetical protein LM5578 2859	gi|284803102|ref|YP 003414967.1	gi|662858600|ref|NC_013766.2|:c2873501-2872689
hypothetical protein LM5578 2860	gi|284803103|ref|YP 003414968.1	gi|662858600|ref|NC_013766.2|:c2875886-2873547
hypothetical protein LM5578 2861	gi|284803104|ref|YP 003414969.1	gi|662858600|ref|NC_013766.2|:2876166-2876846
hypothetical protein LM5578 2907	gi|284803150|ref|YP 003415015.1	gi|662858600|ref|NC_013766.2|:c2910406-2909708

hypothetical protein LM5578 2908	gi|284803151|ref|YP 003415016.1	gi|662858600|ref|NC_013766.2|:c2911637-2910522
hypothetical protein LM5578 2910	gi|284803153|ref|YP 003415018.1	gi|662858600|ref|NC_013766.2|:2912649-2914391
hypothetical protein LM5578 2913	gi|284803156|ref|YP 003415021.1	gi|662858600|ref|NC_013766.2|:c2919477-2917195
hypothetical protein LM5578 2915	gi|284803158|ref|YP 003415023.1	gi|662858600|ref|NC_013766.2|:c2920857-2920225
hypothetical protein LM5578 2920	gi|284803163|ref|YP 003415028.1	gi|662858600|ref|NC_013766.2|:c2923531-2922620
hypothetical protein LM5578 2921	gi|284803164|ref|YP 003415029.1	gi|662858600|ref|NC_013766.2|:c2924359-2923553
hypothetical protein LM5578 2922	gi|284803165|ref|YP 003415030.1	gi|662858600|ref|NC_013766.2|:c2925348-2924383
F0F1 ATP synthase subunit beta	gi|284803169|ref|YP 003415034.1	gi|662858600|ref|NC_013766.2|:c2928710-2927340
F0F1 ATP synthase subunit alpha	gi|284803171|ref|YP 003415036.1	gi|662858600|ref|NC_013766.2|:c2931073-2929577
hypothetical protein LM5578 2964	gi|284803207|ref|YP 003415072.1	gi|662858600|ref|NC_013766.2|:c2973678-2971705
putative accessory gene regulator protein	gi|284803211|ref|YP 003415076.1	gi|662858600|ref|NC_013766.2|:c2976742-2976128
hypothetical protein LM5578 2969	gi|284803212|ref|YP 003415077.1	gi|662858600|ref|NC_013766.2|:c2977610-2976999
arginine deiminase	gi|284803216|ref|YP 003415081.1	gi|662858600|ref|NC_013766.2|:c2980437-2979211
hypothetical protein LM5578 2974	gi|284803217|ref|YP 003415082.1	gi|662858600|ref|NC_013766.2|:c2981182-2980523
hypothetical protein LM5578 2982	gi|284803225|ref|YP 003415090.1	gi|662858600|ref|NC_013766.2|:c2990492-2989134

hypothetical protein LM5578 2983	gi|284803226|ref|YP 003415091.1	gi|662858600|ref|NC_013766.2|:c2992907-2990505
hypothetical protein LM5578 2984	gi|284803227|ref|YP 003415092.1	gi|662858600|ref|NC_013766.2|:c2993869-2992904
LacI family transcription regulator	gi|284803228|ref|YP 003415093.1	gi|662858600|ref|NC_013766.2|:2994071-2995126
hypothetical protein LM5578 2994	gi|284803237|ref|YP 003415102.1	gi|662858600|ref|NC_013766.2|:c3001539-3000664
hypothetical protein LM5578 2996	gi|284803239|ref|YP 003415104.1	gi|662858600|ref|NC_013766.2|:c3004374-3003730
hypothetical protein LM5578 2997	gi|284803240|ref|YP 003415105.1	gi|662858600|ref|NC_013766.2|:c3005130-3004426
hypothetical protein LM5578 2998	gi|284803241|ref|YP 003415106.1	gi|662858600|ref|NC_013766.2|:c3005979-3005158
hypothetical protein LM5578 2999	gi|284803242|ref|YP 003415107.1	gi|662858600|ref|NC_013766.2|:c3006763-3005960
hypothetical protein LM5578 3002	gi|284803245|ref|YP 003415110.1	gi|662858600|ref|NC_013766.2|:c3008431-3007691
hypothetical protein LM5578 3003	gi|284803246|ref|YP 003415111.1	gi|662858600|ref|NC_013766.2|:3008672-3009493
AA3-600 quinol oxidase subunit I	gi|284803251|ref|YP 003415116.1	gi|662858600|ref|NC_013766.2|:c3015281-3013302
hypothetical protein LM5578 3010	gi|284803253|ref|YP 003415118.1	gi|662858600|ref|NC_013766.2|:c3017833-3016751
hypothetical protein LM5578 3012	gi|284803255|ref|YP 003415120.1	gi|662858600|ref|NC_013766.2|:c3019680-3018739
DNA gyrase subunit A	gi|284803259|ref|YP 003415124.1	gi|662858600|ref|NC_013766.2|:c3024560-3022032
DNA gyrase subunit B	gi|284803260|ref|YP 003415125.1	gi|662858600|ref|NC_013766.2|:c3026595-3024655

recombination protein F	gi|284803261|ref|YP 003415126.1	gi|662858600|ref|NC_013766.2|:c3027756-3026644
hypothetical protein LM5578 3020	gi|284803263|ref|YP 003415128.1	gi|662858600|ref|NC_013766.2|:c3029504-3028161
DNA polymerase III subunit beta	gi|284803264|ref|YP 003415129.1	gi|662858600|ref|NC_013766.2|:c3030758-3029613
chromosomal replication initiation protein	gi|284803265|ref|YP 003415130.1	gi|662858600|ref|NC_013766.2|:c3032307-3030952
