# Supplementary material for: The Listeria monocytogenes Core-Genome Sequence Typer (LmCGST): a bioinformatic pipeline for molecular characterization with next-generation sequence data
Source: BMC Microbiol. 2015 Oct 22;15:224. doi: 10.1186/s12866-015-0526-1 (PMC4618880; doi:10.1186/s12866-015-0526-1)
Supplement: Additional file 5: — Comparisons of the frequencies of proteins within Clusters of Orthologous Groups categories. Frequencies of different functional classes of proteins encoded by open-reading frames from a complete Listeria monocytogenes chromosome (08–5578) and the high-confidence core. (PDF 117 kb) [file 12866_2015_526_MOESM5_ESM.pdf]

Supporting Information for:

The *Listeria monocytogenes* Core-Genome Sequence Typer (LmCGST): a bioinformatic pipeline for molecular characterization with next-generation sequence data

Arthur W. Pightling[1], Nicholas Petronella[2], Franco Pagotto[1*]

[1] Listeriosis Reference Service for Canada, Microbiology Research Division, Bureau of Microbial Hazards, Food Directorate, Health Products and Food Branch, Health Canada, 251 Sir Frederick Banting Driveway, Ottawa, Ontario, K1A 0K9 Canada

[2] Biostatistics and Modelling Division, Bureau of Food Surveillance and Science Integration, Food Directorate, Health Products and Food Branch, Health Canada, 251 Sir Frederick Banting Driveway, Ottawa, Ontario, K1A 0K9 Canada

[*] Corresponding author

E-mails:

Franco Pagotto: Franco.Pagotto@hc-sc.gc.ca
Arthur Pightling: Arthur.Pightling@hc-sc.gc.ca
Nicholas Petronella: Nicholas.Petronella@hc-sc.gc.ca

Additional file 5: Comparisons of the frequencies of proteins within Clusters of Orthologous Groups categories. The entire set of *L. monocytogenes* strain 08-5578 predicted protein sequences were categorized and compared to the predicted proteins encoded by 1013 open-reading frames present within the calculated high-confidence core-genome.

Additional file 5

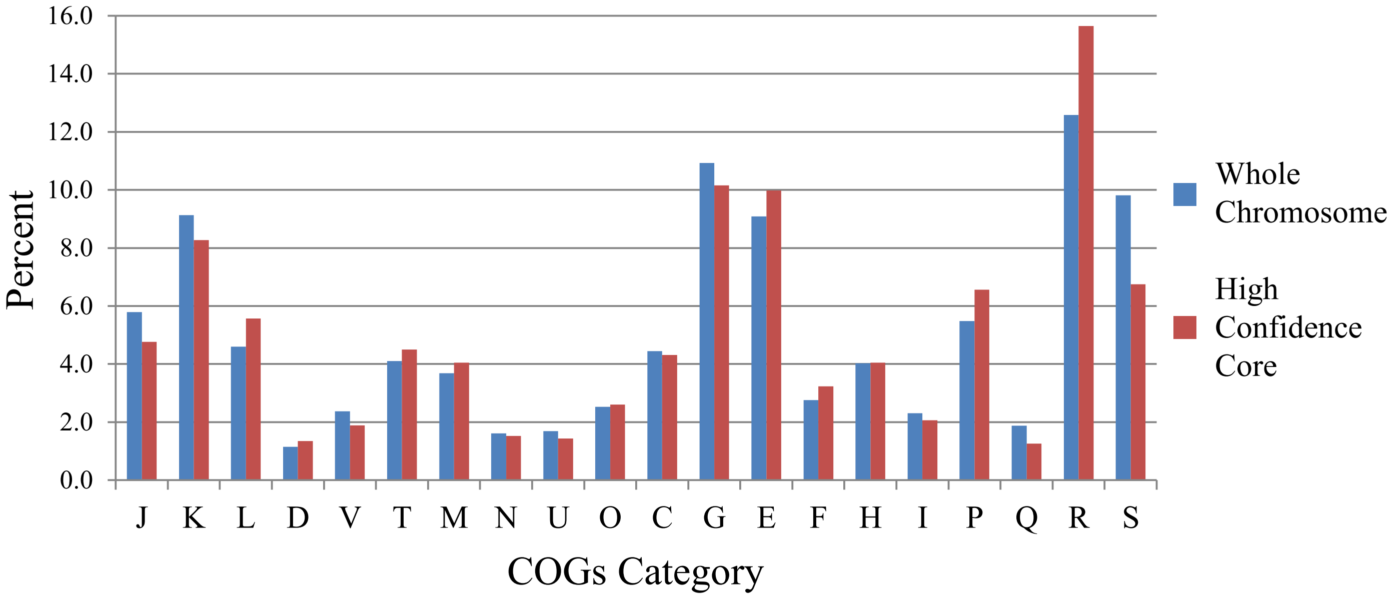

INFORMATION STORAGE AND PROCESSING
J = Translation, ribosomal structure and biogenesis
K = Transcription
L = Replication, recombination and repair

CELLULAR PROCESSES AND SIGNALING
D = Cell cycle control, cell division and chromosome partitioning
V = Defense mechanisms
T = Signal transduction mechanisms
M = Cell wall/membrane/envelope biogenesis
N = Cell motility
U = Intracellular trafficking secretion and vesicular transport
O = Posttranslational modification, protein turnover and chaperones

METABOLISM
C = Energy production and conversion
G = Carbohydrate transport and metabolism
E = Amino acid transport and metabolism
F = Nucleotide transport and metabolism
H = Coenzyme transport and metabolism
I = Lipid transport and metabolism
P = Inorganic ion transport and metabolism
Q = Secondary metabolites biosynthesis, transport and catabolism

POORLY CHARACTERIZED
R = General function prediction only
S = Function unknown
