# Supplementary material for: The Listeria monocytogenes Core-Genome Sequence Typer (LmCGST): a bioinformatic pipeline for molecular characterization with next-generation sequence data
Source: BMC Microbiol. 2015 Oct 22;15:224. doi: 10.1186/s12866-015-0526-1 (PMC4618880; doi:10.1186/s12866-015-0526-1)
Supplement: Additional file 8: — Listeria monocytogenes high-confidence core profiles grouped by nucleotide distances. Proposed nomenclature in which Listeria monocytogenes high-confidence core profiles are grouped by nucleotide distances. (PDF 144 kb) [file 12866_2015_526_MOESM8_ESM.pdf]

Supporting Information for:

The *Listeria monocytogenes* Core-Genome Sequence Typer (LmCGST): a bioinformatic pipeline for molecular characterization with next-generation sequence data

Arthur W. Pightling[1], Nicholas Petronella[2], Franco Pagotto[1*]

[1] Listeriosis Reference Service for Canada, Microbiology Research Division, Bureau of Microbial Hazards, Food Directorate, Health Products and Food Branch, Health Canada, 251 Sir Frederick Banting Driveway, Ottawa, Ontario, K1A 0K9 Canada

[2] Biostatistics and Modelling Division, Bureau of Food Surveillance and Science Integration, Food Directorate, Health Products and Food Branch, Health Canada, 251 Sir Frederick Banting Driveway, Ottawa, Ontario, K1A 0K9 Canada

[*] Corresponding author

E-mails:

Franco Pagotto: Franco.Pagotto@hc-sc.gc.ca
Arthur Pightling: Arthur.Pightling@hc-sc.gc.ca
Nicholas Petronella: Nicholas.Petronella@hc-sc.gc.ca

Additional file 8: *Listeria monocytogenes* high-confidence core profiles grouped by nucleotide distances. A cladogram was calculated by aligning and concatenating 1,013 loci that comprise the *L. monocytogenes* high-confidence core (HCC) genomes of 114 taxa and analyzing the resulting alignment of 1,067,173 nucleotide positions with the Randomized Axelerated Maximum Likelihood tool (GTRCATI+25γ). The best of 100 bootstrap replicates is shown. Nucleotide distances were measured with PHYLIP. Taxa were grouped by evolutionary lineage (I, II, or III) and those that have 100 and 10 or fewer nucleotide differences, while unique HCC profiles that differ by no more than 10 nucleotides were numbered in the order that they were processed.

Additional file 8

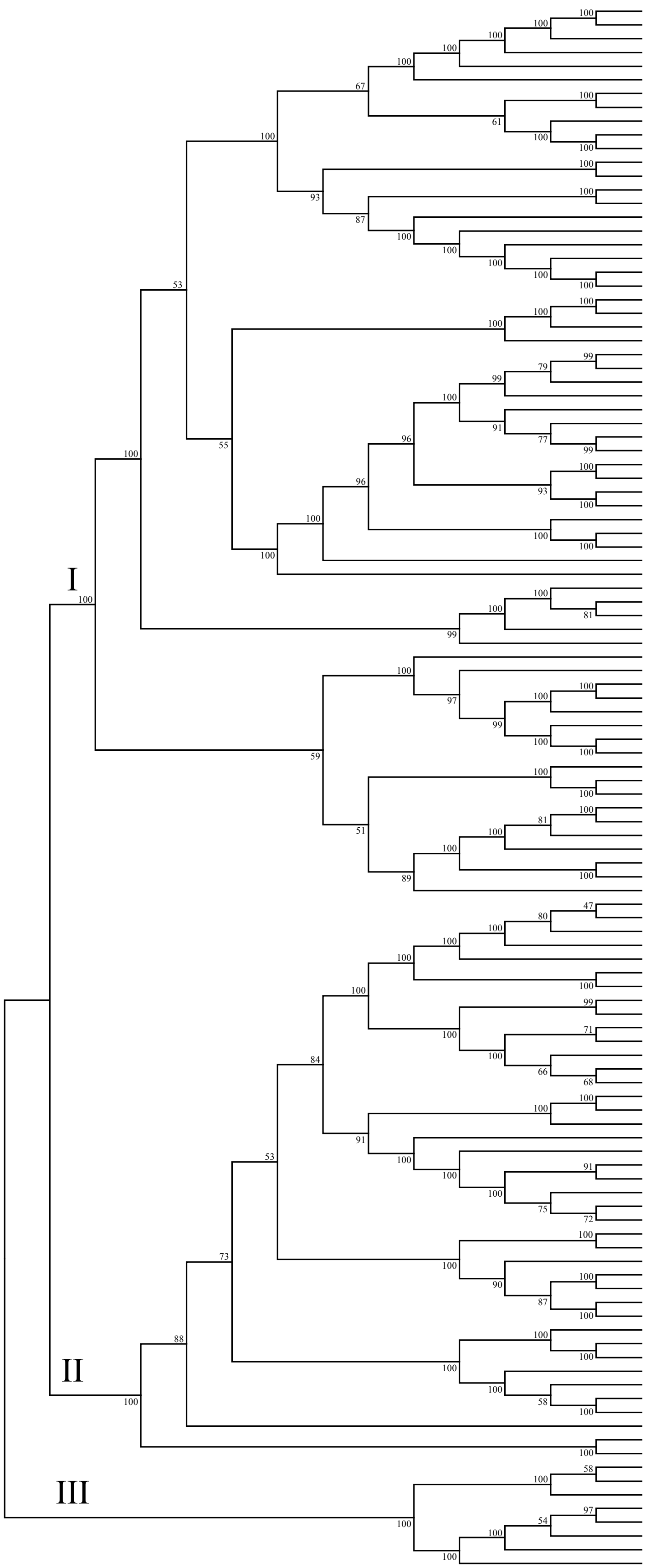

Strain	Lineage	100	10	Ind
HPB962	1	1	1	1
HPB963	1	1	1	2
HPB1013	1	1	1	3
HPB1679	1	1	2	1
CC70B	1	1	3	1
HPB862	1	1	4	1
LL195	1	1	5	1
HPB1077	1	1	6	1
SLCC2378	1	1	7	1
HPB37	1	1	8	1
HPB1042	1	1	8	2
HPB1174	1	1	9	1
HPB1175	1	1	9	2
HPB1074	1	1	10	1
HPB1102	1	1	10	2
HPB7	1	1	11	1
HPB8	1	1	11	2
HPB9	1	1	11	3
HPB23	1	1	11	4
HPB396	1	1	11	5
F2365	1	1	12	1
J1816	1	2	1	1
J1817	1	2	1	2
J1776	1	2	2	1
HPB2141	1	2	3	1
HPB769	1	3	1	1
HPB770	1	3	1	2
HPB771	1	3	1	3
HPB772	1	3	1	4
HPB773	1	3	1	5
HPB774	1	3	1	6
HPB1015	1	3	1	7
HPB1027	1	3	1	8
HPB1014	1	3	2	1
HPB1092	1	3	2	2
HPB2263	1	3	3	1
HPB2264	1	3	3	2
J1-220	1	3	4	1
HPB7171	1	3	5	1
SCOTT-A	1	3	5	2
ATCC-191	1	3	6	1
HPB1945	1	4	1	1
CLIP80459	1	5	1	1
HPB960	1	5	2	1
L312	1	5	3	1
07PF0776	1	6	1	1
HPB6355	1	7	1	1
HPB1031	1	8	5	1
SLCC2482	1	8	4	1
HPB394	1	8	1	1
HPB1111	1	8	1	2
SLCC2755	1	8	2	1
R2-502	1	8	3	3
HPB2130	1	8	3	1
HPB2134	1	8	3	2
HPB5252	1	9	1	1
HPB2129	1	10	1	1
HPB5767	1	10	2	1
HPB1340	1	11	1	1
HPB1341	1	11	1	2
HPB518	1	11	2	1
J2-064	1	11	3	1
Lm19860	1	11	4	1
Lm26686	1	11	4	2
SLCC2540	1	12	1	1
SLCC2372	2	1	1	1
SLCC2479	2	1	2	1
FSL-R2-56	2	1	3	1
HPB2921	2	1	4	1
EGD-e	2	2	1	1
La111	2	3	1	1
N53-1	2	3	2	1
HPB1707	2	4	1	1
HPB2412	2	5	1	1
F6900	2	6	1	1
HPB1022	2	6	1	2
HPB1045	2	6	1	3
HPB1075	2	6	1	4
J2818	2	6	1	5
HPB1225	2	7	1	1
SLCC7179	2	8	1	1
Lm25180	2	9	1	1
Lm21045	2	10	1	1
HPB976	2	10	2	1
08-5578	2	10	3	1
08-5923	2	10	3	2
HPB913	2	10	3	3
HPB2088	2	10	3	4
HPB5415	2	10	3	5
10403S	2	11	1	1
HPB2980	2	11	2	1
HPB2386	2	11	3	1
HPB2865	2	11	4	1
HPB2866	2	11	4	2
SLCC5850	2	11	5	1
WSLC1001	2	11	5	2
HPB4	2	12	1	1
HPB285	2	12	2	1
HPB1654	2	12	3	1
C1-387	2	13	1	1
HPB2	2	13	2	1
Finland-98	2	13	3	1
HPB2864	2	13	3	2
HPB2166	2	14	1	1
HPB424	2	15	1	1
NCCP	2	16	1	1
HCC23	3	1	1	1
L99	3	1	1	2
M7	3	1	1	3
HPB330	3	2	1	1
HPB1631	3	2	1	2
HPB535	3	2	2	1
HPB5209	3	2	3	1
SLCC2376	3	3	1	1
