# Supplementary material for: The Listeria monocytogenes Core-Genome Sequence Typer (LmCGST): a bioinformatic pipeline for molecular characterization with next-generation sequence data
Source: BMC Microbiol. 2015 Oct 22;15:224. doi: 10.1186/s12866-015-0526-1 (PMC4618880; doi:10.1186/s12866-015-0526-1)

Supporting Information for:

The *Listeria monocytogenes* Core-Genome Sequence Typer (LmCGST): a bioinformatic pipeline for molecular characterization with next-generation sequence data

Arthur W. Pightling[1], Nicholas Petronella[2], Franco Pagotto[1*]

[1] Listeriosis Reference Service for Canada, Microbiology Research Division, Bureau of Microbial Hazards, Food Directorate, Health Products and Food Branch, Health Canada, 251 Sir Frederick Banting Driveway, Ottawa, Ontario, K1A 0K9 Canada

[2] Biostatistics and Modelling Division, Bureau of Food Surveillance and Science Integration, Food Directorate, Health Products and Food Branch, Health Canada, 251 Sir Frederick Banting Driveway, Ottawa, Ontario, K1A 0K9 Canada

[*] Corresponding author

E-mails:

Franco Pagotto: Franco.Pagotto@hc-sc.gc.ca
Arthur Pightling: Arthur.Pightling@hc-sc.gc.ca
Nicholas Petronella: Nicholas.Petronella@hc-sc.gc.ca

Additional file 9: Phylogenetic analysis of 115 high-confidence core profiles. Sequence data for 1013 loci that comprise the high-confidence core were collected from all members of the core-genome sequence typing database and a single randomly selected subject (red). Sequences were then aligned and concatenated. Supermatrices were analyzed with the PHYLIP “dnadist” (F84 and default settings) and “neighbor” (Neighbor-joining approach with default settings) modules. Roman numerals (I, II, and III) identify the three currently recognized evolutionary lineages.

Additional file 9

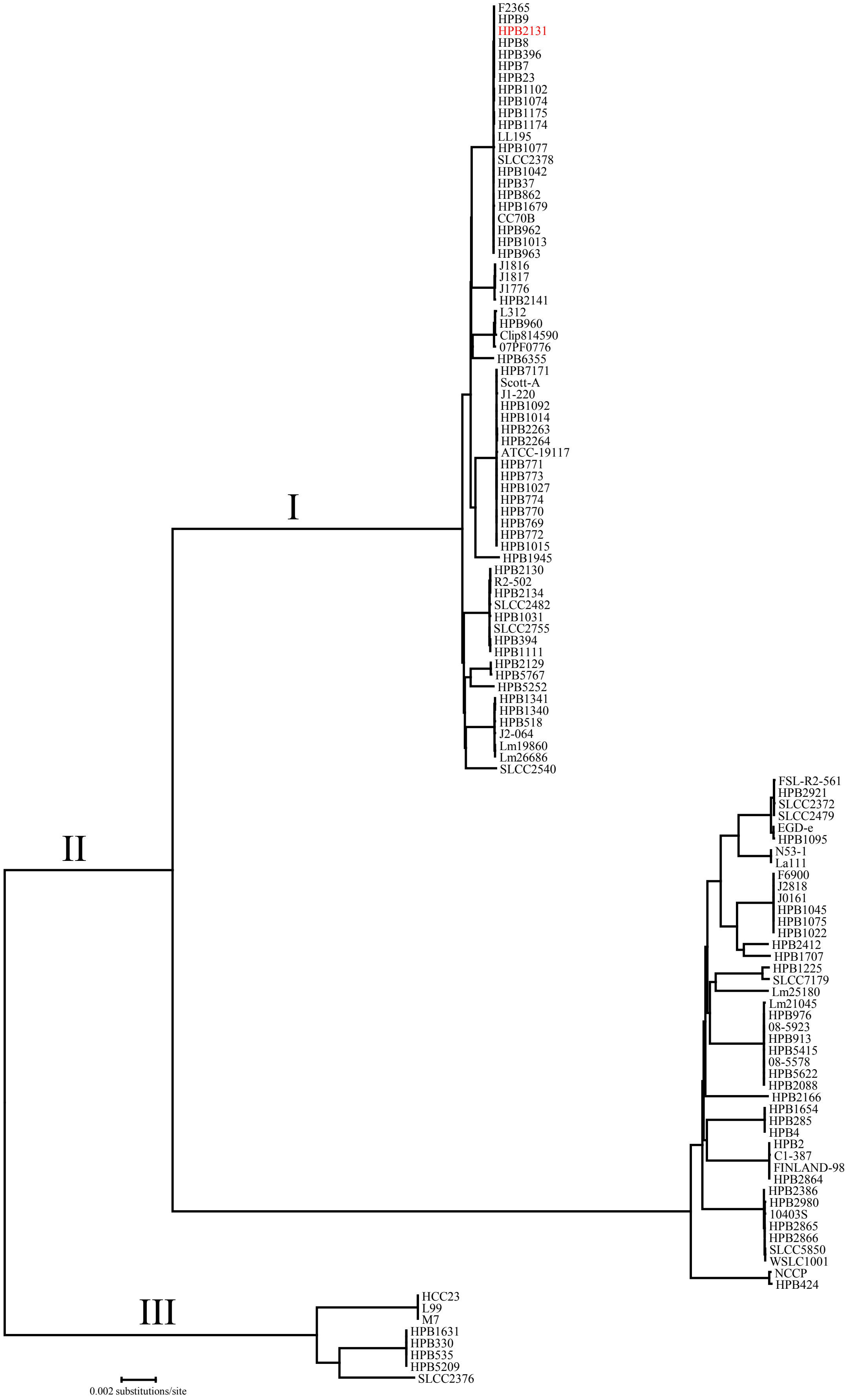

Supplement: Additional file 9: — Phylogenetic analysis of 115 high-confidence core profiles. Phylogenetic relationships of 114 HCC profiles that comprise the CGST database and a single randomly selected test subject. (PDF 112 kb) [file 12866_2015_526_MOESM9_ESM.pdf]
