# Supplementary material for: The Listeria monocytogenes Core-Genome Sequence Typer (LmCGST): a bioinformatic pipeline for molecular characterization with next-generation sequence data
Source: BMC Microbiol. 2015 Oct 22;15:224. doi: 10.1186/s12866-015-0526-1 (PMC4618880; doi:10.1186/s12866-015-0526-1)

Supporting Information for:

The *Listeria monocytogenes* Core-Genome Sequence Typer (LmCGST): a bioinformatic pipeline for molecular characterization with next-generation sequence data

Arthur W. Pightling[1], Nicholas Petronella[2], Franco Pagotto[1*]

[1] Listeriosis Reference Service for Canada, Microbiology Research Division, Bureau of Microbial Hazards, Food Directorate, Health Products and Food Branch, Health Canada, 251 Sir Frederick Banting Driveway, Ottawa, Ontario, K1A 0K9 Canada

[2] Biostatistics and Modelling Division, Bureau of Food Surveillance and Science Integration, Food Directorate, Health Products and Food Branch, Health Canada, 251 Sir Frederick Banting Driveway, Ottawa, Ontario, K1A 0K9 Canada

[*] Corresponding author

E-mails:

Franco Pagotto: Franco.Pagotto@hc-sc.gc.ca
Arthur Pightling: Arthur.Pightling@hc-sc.gc.ca
Nicholas Petronella: Nicholas.Petronella@hc-sc.gc.ca

Additional file 10: Comparison of 12 sets of Illumina MiSeq data of varying qualities. We sequenced *Listeria monocytogenes* strain 08-5578 genomic DNA 12 times. All reads passing the chastity filter implemented by the Illumina MiSeq reporter software were assembled using SPAdes v3.0.0 and the BayesHammer correction tool. We then annotated the assemblies with Prokka v1.10 and searched for the 1013 open-reading frames (ORFs) that compose the high-confidence core. For each set, we noted the numbers of false single-nucleotide polymorphisms (SNPs) observed (a), the numbers of missing ORFs (b), and the numbers of partial ORFs (c).

Additional file 10

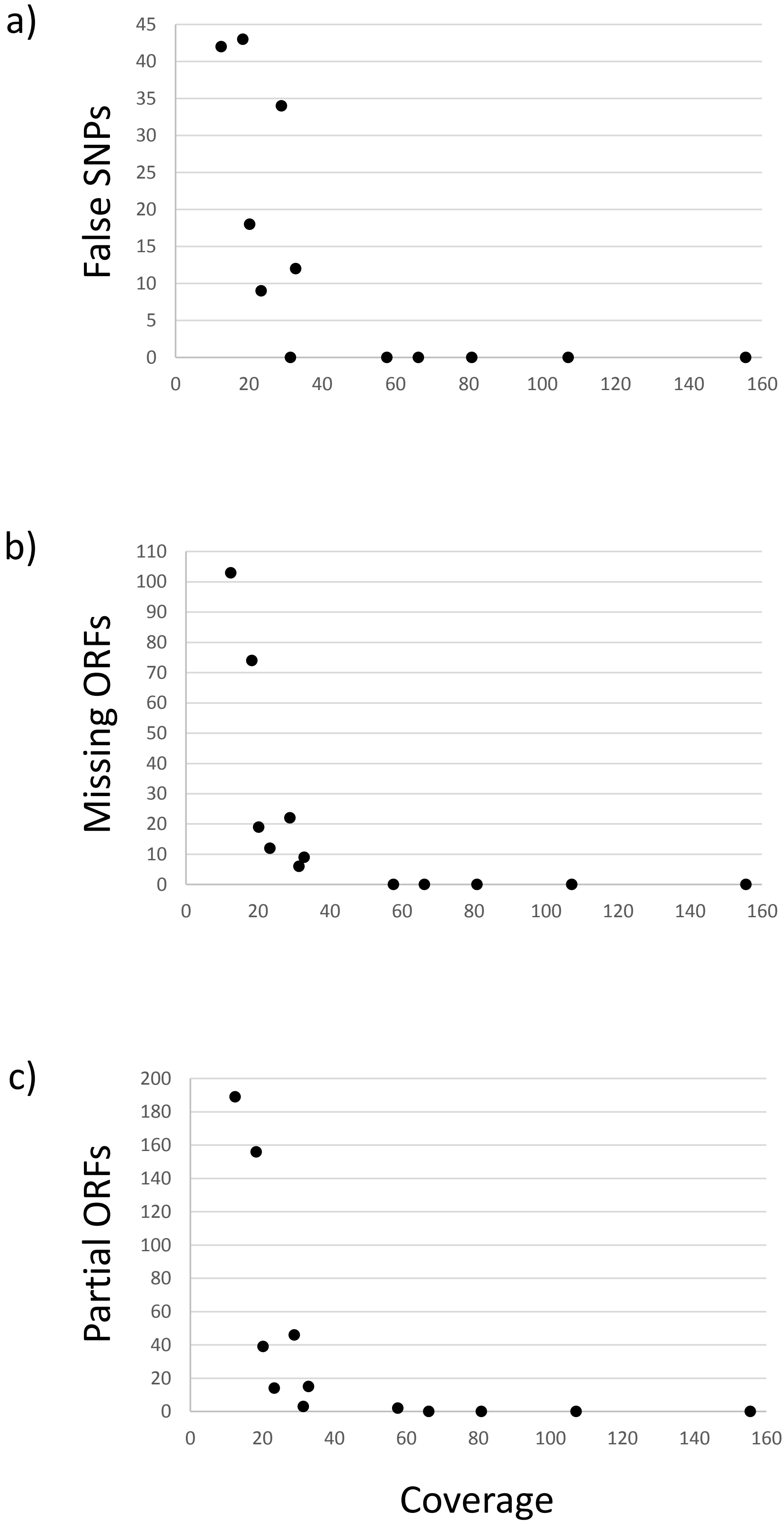

Supplement: Additional file 10: — Comparison of 12 sets of Illumina MiSeq data of varying qualities. Numbers of: a) SNPs, b) missing ORFs, and c) partial ORFs observed when genome sequence datasets of low to high quality are analyzed using the LmCGST pipeline. (PDF 112 kb) [file 12866_2015_526_MOESM10_ESM.pdf]
