# Supplementary material for: The Listeria monocytogenes Core-Genome Sequence Typer (LmCGST): a bioinformatic pipeline for molecular characterization with next-generation sequence data
Source: BMC Microbiol. 2015 Oct 22;15:224. doi: 10.1186/s12866-015-0526-1 (PMC4618880; doi:10.1186/s12866-015-0526-1)
Supplement: Additional file 11: — Summary statistics describing data obtained from Illumina sequencing runs of Listeria monocytogenes strain 08–5578 genomic DNA samples. (PDF 97 kb) [file 12866_2015_526_MOESM11_ESM.pdf]

Supporting Information for:

The *Listeria monocytogenes* Core-Genome Sequence Typer (LmCGST): a bioinformatic pipeline for molecular characterization with next-generation sequence data

Arthur W. Pightling[1], Nicholas Petronella[2], Franco Pagotto[1*]

[1] Listeriosis Reference Service for Canada, Microbiology Research Division, Bureau of Microbial Hazards, Food Directorate, Health Products and Food Branch, Health Canada, 251 Sir Frederick Banting Driveway, Ottawa, Ontario, K1A 0K9 Canada

[2] Biostatistics and Modelling Division, Bureau of Food Surveillance and Science Integration, Food Directorate, Health Products and Food Branch, Health Canada, 251 Sir Frederick Banting Driveway, Ottawa, Ontario, K1A 0K9 Canada

[*] Corresponding author

E-mails:

Franco Pagotto: Franco.Pagotto@hc-sc.gc.ca
Arthur Pightling: Arthur.Pightling@hc-sc.gc.ca
Nicholas Petronella: Nicholas.Petronella@hc-sc.gc.ca

Additional file 11: Summary statistics describing data obtained from Illumina sequencing runs of *Listeria monocytogenes* strain 08-5578 genomic DNA samples.

Run	Yield (Gb)	Average Cluster Density (k/mm^2)	Total Reads	Reads Passing Filter
1	3.96	478.5	9357411	8506127
2	1.16	178.5	5077244	2302912
3	7.76	1075.0	19068628	16320666

Note: Genomic DNA was extracted and divided into four subsamples. Each subsample was sequenced during three separate runs on an Illumina MiSeq benchtop sequencer.
