# Supplementary material for: The Listeria monocytogenes Core-Genome Sequence Typer (LmCGST): a bioinformatic pipeline for molecular characterization with next-generation sequence data
Source: BMC Microbiol. 2015 Oct 22;15:224. doi: 10.1186/s12866-015-0526-1 (PMC4618880; doi:10.1186/s12866-015-0526-1)
Supplement: Additional file 12: — Processing times for steps of the LmCGST pipeline with different database sizes. (PDF 86 kb) [file 12866_2015_526_MOESM12_ESM.pdf]

Supporting Information for:

The *Listeria monocytogenes* Core-Genome Sequence Typer (LmCGST): a bioinformatic pipeline for molecular characterization with next-generation sequence data

Arthur W. Pightling[1], Nicholas Petronella[2], Franco Pagotto[1*]

[1] Listeriosis Reference Service for Canada, Microbiology Research Division, Bureau of Microbial Hazards, Food Directorate, Health Products and Food Branch, Health Canada, 251 Sir Frederick Banting Driveway, Ottawa, Ontario, K1A 0K9 Canada

[2] Biostatistics and Modelling Division, Bureau of Food Surveillance and Science Integration, Food Directorate, Health Products and Food Branch, Health Canada, 251 Sir Frederick Banting Driveway, Ottawa, Ontario, K1A 0K9 Canada

[*] Corresponding author

E-mails:

Franco Pagotto: Franco.Pagotto@hc-sc.gc.ca
Arthur Pightling: Arthur.Pightling@hc-sc.gc.ca
Nicholas Petronella: Nicholas.Petronella@hc-sc.gc.ca

Additional file 12: Processing times for steps of the LmCGST pipeline with different database sizes. We estimated the amount of time necessary to assemble and annotate a randomly selected set of *Listeria monocytogenes* short-read sequence data with SPAdes v3.0.0 and Prokka v1.10. In addition, we ran the LmCGST pipeline with databases populated by 25, 50, and 100 high-confidence core (HCC) genomes in order to measure times for HCC profile comparisons and phylogenetic analyses. The pipeline was run on a desktop computer with an AMD Phenom II X6 1090T processor and 16GB of DDR3 RAM using 5 cores.

	Assembly	Annotation	HCC Comparisons			Phylogenies			Totals		
			25	50	100	25	50	100	25	50	100
Time (hours)	2.69	0.095	0.013	0.027	0.059	0.089	0.19	0.46	2.89	3.01	3.31
