# Supplementary material for: The Listeria monocytogenes Core-Genome Sequence Typer (LmCGST): a bioinformatic pipeline for molecular characterization with next-generation sequence data
Source: BMC Microbiol. 2015 Oct 22;15:224. doi: 10.1186/s12866-015-0526-1 (PMC4618880; doi:10.1186/s12866-015-0526-1)

Supporting Information for:

The *Listeria monocytogenes* Core-Genome Sequence Typer (LmCGST): a bioinformatic pipeline for molecular characterization with next-generation sequence data

Arthur W. Pightling[1], Nicholas Petronella[2], Franco Pagotto[1*]

[1] Listeriosis Reference Service for Canada, Microbiology Research Division, Bureau of Microbial Hazards, Food Directorate, Health Products and Food Branch, Health Canada, 251 Sir Frederick Banting Driveway, Ottawa, Ontario, K1A 0K9 Canada

[2] Biostatistics and Modelling Division, Bureau of Food Surveillance and Science Integration, Food Directorate, Health Products and Food Branch, Health Canada, 251 Sir Frederick Banting Driveway, Ottawa, Ontario, K1A 0K9 Canada

[*] Corresponding author

E-mails:

Franco Pagotto: Franco.Pagotto@hc-sc.gc.ca
Arthur Pightling: Arthur.Pightling@hc-sc.gc.ca
Nicholas Petronella: Nicholas.Petronella@hc-sc.gc.ca

Additional file 13: Predicted LmCGST processing times with increasing database sizes. The amount of time necessary to assemble *Listeria monocytogenes* short-read sequences with SPAdes v3.0.0 and to annotate the assembly with Prokka v1.10 was measured. In addition, we predicted the amount of time necessary to perform high-confidence core genome comparisons and phylogenetic analyses using the PHYLIP package with increasing database sizes. The pipeline was run on a desktop computer with an AMD Phenom II X6 1090T processor and 16GB of DDR3 RAM using 5 cores.

Additional file 13

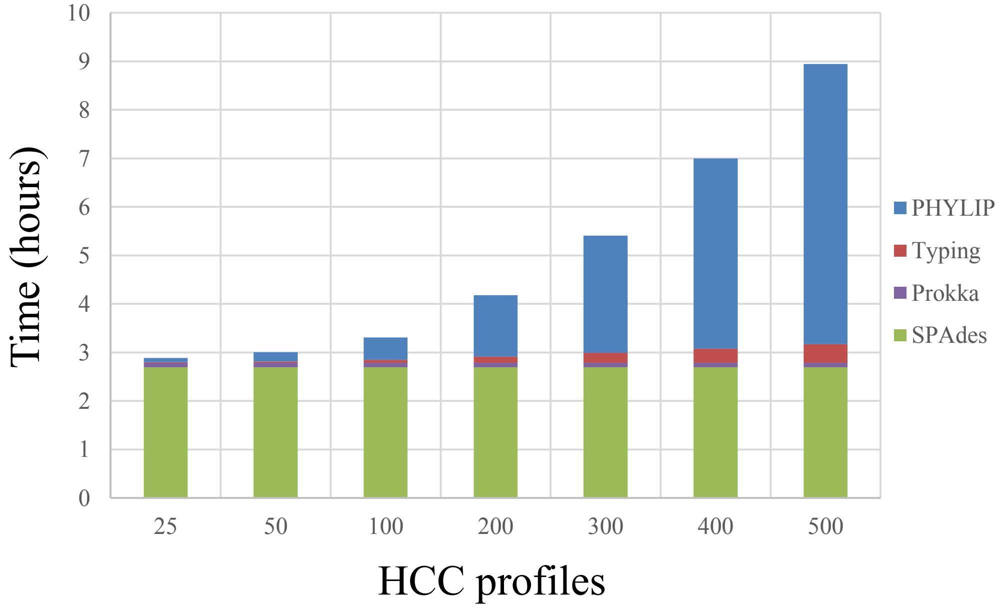

Supplement: Additional file 13: — Predicted LmCGST processing times with increasing database sizes. Amounts of time predicted to be required for the assembly, annotation, HCC comparison, and tree-building steps with database sizes from 25 to 500 HCC profiles. (PDF 108 kb) [file 12866_2015_526_MOESM13_ESM.pdf]
