# Supplementary material for: The Listeria monocytogenes Core-Genome Sequence Typer (LmCGST): a bioinformatic pipeline for molecular characterization with next-generation sequence data
Source: BMC Microbiol. 2015 Oct 22;15:224. doi: 10.1186/s12866-015-0526-1 (PMC4618880; doi:10.1186/s12866-015-0526-1)
Supplement: Additional file 14: — Simpson’s index of diversity and 95 % confidence intervals. Measurements of the probabilities that two strains sampled randomly from a population will be assigned to two different types with the CGST, PFGE, ribotyping, and MLST methods. (PDF 85 kb) [file 12866_2015_526_MOESM14_ESM.pdf]

Supporting Information for:

The *Listeria monocytogenes* Core-Genome Sequence Typer (LmCGST): a bioinformatic pipeline for molecular characterization with next-generation sequence data

Arthur W. Pightling[1], Nicholas Petronella[2], Franco Pagotto[1*]

[1] Listeriosis Reference Service for Canada, Microbiology Research Division, Bureau of Microbial Hazards, Food Directorate, Health Products and Food Branch, Health Canada, 251 Sir Frederick Banting Driveway, Ottawa, Ontario, K1A 0K9 Canada

[2] Biostatistics and Modelling Division, Bureau of Food Surveillance and Science Integration, Food Directorate, Health Products and Food Branch, Health Canada, 251 Sir Frederick Banting Driveway, Ottawa, Ontario, K1A 0K9 Canada

[*] Corresponding author

E-mails:

Franco Pagotto: Franco.Pagotto@hc-sc.gc.ca
Arthur Pightling: Arthur.Pightling@hc-sc.gc.ca
Nicholas Petronella: Nicholas.Petronella@hc-sc.gc.ca

Additional file 14: Simpson's index of diversity and 95% confidence intervals. These measurements indicate the probabilities that two strains sampled randomly from a population will be assigned to two different types.

**Name**	**Number of Partitions**	**Simpson's ID**	**Confidence Interval (95%)**
CGST	73	0.995	0.990-1.000
PFGE+Ribo	48	0.972	0.957-0.988
PFGE	48	0.972	0.957-0.988
ApaI	43	0.968	0.952-0.984
AscI	39	0.965	0.949-0.981
ribotype	25	0.899	0.861-0.936
MLST	25	0.889	0.844-0.934

Note: ApaI and AscI data were combined to generate the PFGE category and ApaI, AscI, and ribotype data were combined to generate the PFGE+Ribo category.
