# Supplementary material for: The Listeria monocytogenes Core-Genome Sequence Typer (LmCGST): a bioinformatic pipeline for molecular characterization with next-generation sequence data
Source: BMC Microbiol. 2015 Oct 22;15:224. doi: 10.1186/s12866-015-0526-1 (PMC4618880; doi:10.1186/s12866-015-0526-1)

Supporting Information for:

The *Listeria monocytogenes* Core-Genome Sequence Typer (LmCGST): a bioinformatic pipeline for molecular characterization with next-generation sequence data

Arthur W. Pightling[1], Nicholas Petronella[2], Franco Pagotto[1*]

[1] Listeriosis Reference Service for Canada, Microbiology Research Division, Bureau of Microbial Hazards, Food Directorate, Health Products and Food Branch, Health Canada, 251 Sir Frederick Banting Driveway, Ottawa, Ontario, K1A 0K9 Canada

[2] Biostatistics and Modelling Division, Bureau of Food Surveillance and Science Integration, Food Directorate, Health Products and Food Branch, Health Canada, 251 Sir Frederick Banting Driveway, Ottawa, Ontario, K1A 0K9 Canada

[*] Corresponding author

E-mails:

Franco Pagotto: Franco.Pagotto@hc-sc.gc.ca
Arthur Pightling: Arthur.Pightling@hc-sc.gc.ca
Nicholas Petronella: Nicholas.Petronella@hc-sc.gc.ca

Additional file 16: Phylogenetic analysis of *de-novo* assemblies calculated from short-read sequence data obtained from four sequencing runs of a single *Listeria monocytogenes* strain 08-5578 DNA extraction. DNA was isolated and divided into four subsamples. The subsamples were analyzed on an Illumina MiSeq benchtop sequencer. Reads were assembled *de-novo* with SPAdes v3.0.0, aligned with Mauve v2.3.1, and 3,138,152 nucleotide positions were phylogenetically analyzed with RAxML v8.1.1 (GTRCATI+25γ). The best of 100 bootstrap replicates is shown. The estimated sequence coverage for each assembly are indicated in parenthesis.

Additional file 16

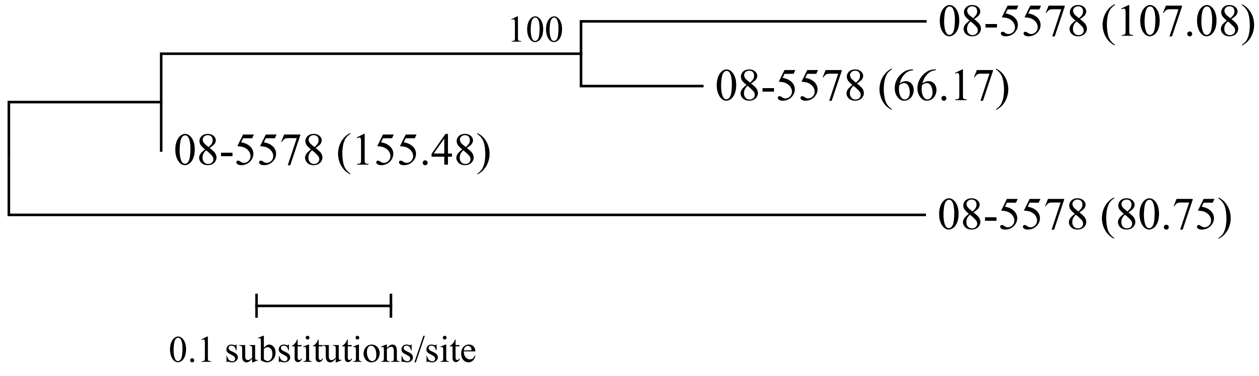

Supplement: Additional file 16: — Phylogenetic analysis of de-novo assemblies calculated from short-read sequence data obtained from four sequencing runs of a single Listeria monocytogenes strain 08–5578 DNA extraction. (PDF 117 kb) [file 12866_2015_526_MOESM16_ESM.pdf]
