# Supplementary material for: The Listeria monocytogenes Core-Genome Sequence Typer (LmCGST): a bioinformatic pipeline for molecular characterization with next-generation sequence data
Source: BMC Microbiol. 2015 Oct 22;15:224. doi: 10.1186/s12866-015-0526-1 (PMC4618880; doi:10.1186/s12866-015-0526-1)
Supplement: Additional file 17: — Distance analysis of de-novo assemblies calculated from short-read sequence data obtained from four sequencing runs of a single Listeria monocytogenes strain 08–5578 DNA extraction. (PDF 96 kb) [file 12866_2015_526_MOESM17_ESM.pdf]

Supporting Information for:

The *Listeria monocytogenes* Core-Genome Sequence Typer (LmCGST): a bioinformatic pipeline for molecular characterization with next-generation sequence data

Arthur W. Pightling[1], Nicholas Petronella[2], Franco Pagotto[1*]

[1] Listeriosis Reference Service for Canada, Microbiology Research Division, Bureau of Microbial Hazards, Food Directorate, Health Products and Food Branch, Health Canada, 251 Sir Frederick Banting Driveway, Ottawa, Ontario, K1A 0K9 Canada

[2] Biostatistics and Modelling Division, Bureau of Food Surveillance and Science Integration, Food Directorate, Health Products and Food Branch, Health Canada, 251 Sir Frederick Banting Driveway, Ottawa, Ontario, K1A 0K9 Canada

[*] Corresponding author

E-mails:

Franco Pagotto: Franco.Pagotto@hc-sc.gc.ca
Arthur Pightling: Arthur.Pightling@hc-sc.gc.ca
Nicholas Petronella: Nicholas.Petronella@hc-sc.gc.ca

Additional file 17: Distance analysis of *de-novo* assemblies calculated from short-read sequence data obtained from four sequencing runs of a single *Listeria monocytogenes* strain 08-5578 DNA extraction.

	08-5578 (66.17)	08-5578 (80.75)	08-5578 (107.08)
08-5578 (80.75)	0.000020/62.76		
08-5578 (107.08)	0.000009/28.24	0.000030/94.14	
08-5578 (155.48)	0.000006/18.83	0.000014/43.93	0.000013/40.80

Note: DNA was isolated and divided into four subsamples. The subsamples were analyzed on an Illumina MiSeq benchtop sequencer. Reads were assembled *de-novo* with SPAdes v3.0.0, aligned with Mauve v2.3.1, and distance were calculated from 3,138,152 nucleotide positions with PHYLIP. The estimated sequence coverage for each assembly are indicated in parenthesis. Distances are shown with the estimated numbers of differences between chromosome sequences separated by a forward slash.
