# Supplementary material for: The Listeria monocytogenes Core-Genome Sequence Typer (LmCGST): a bioinformatic pipeline for molecular characterization with next-generation sequence data
Source: BMC Microbiol. 2015 Oct 22;15:224. doi: 10.1186/s12866-015-0526-1 (PMC4618880; doi:10.1186/s12866-015-0526-1)
Supplement: Additional file 18: — Data used to calculate unique Listeria monocytogenes high-confidence core genomes. (PDF 109 kb) [file 12866_2015_526_MOESM18_ESM.pdf]

Supporting Information for:

The *Listeria monocytogenes* Core-Genome Sequence Typer (LmCGST): a bioinformatic pipeline for molecular characterization with next-generation sequence data

Arthur W. Pightling[1], Nicholas Petronella[2], Franco Pagotto[1*]

[1] Listeriosis Reference Service for Canada, Microbiology Research Division, Bureau of Microbial Hazards, Food Directorate, Health Products and Food Branch, Health Canada, 251 Sir Frederick Banting Driveway, Ottawa, Ontario, K1A 0K9 Canada

[2] Biostatistics and Modelling Division, Bureau of Food Surveillance and Science Integration, Food Directorate, Health Products and Food Branch, Health Canada, 251 Sir Frederick Banting Driveway, Ottawa, Ontario, K1A 0K9 Canada

[*] Corresponding author

E-mails:

Franco Pagotto: Franco.Pagotto@hc-sc.gc.ca
Arthur Pightling: Arthur.Pightling@hc-sc.gc.ca
Nicholas Petronella: Nicholas.Petronella@hc-sc.gc.ca

Additional file 18: Data used to calculate unique *Listeria monocytogenes* high-confidence core genomes.

Strain	NCBI Database	NCBI Number
CC70B	Short-read archive	SRR835447
J1776	Nucleotide	CP006598.1
Scott-A	Nucleotide	NZ_CM001159.1
R2-502	Nucleotide	CP006594.1
J2-064	Nucleotide	CP006592.1
Lm19860	Nucleotide	KK211032.1
Lm26686	Nucleotide	KK211038.1
F6900	Nucleotide	NZ_AARU02000001.1 – NZ_AARU020000023.1
J2818	Nucleotide	NZ_AARX02000001.1 – NZ_AARX02000024.1
Lm25180	Nucleotide	KK211191.1
Lm21045	Nucleotide	KK211193.1
WSLC1001	Nucleotide	CP007160.1
C1-387	Nucleotide	CP006591.1
NCCP_15743	Nucleotide	NZ_APJT01000001.1 – NZ_APJT01000005.1
HPB1013	Short-read archive	SRR1639783
HPB1014	Short-read archive	SRR1640020
HPB1015	Short-read archive	SRR1640113
HPB1022	Short-read archive	SRR1640203
HPB1027	Short-read archive	SRR1640093
HPB1031	Short-read archive	SRR1640143
HPB1042	Short-read archive	SRR1639796
HPB1045	Short-read archive	SRR1640206
HPB1074	Short-read archive	SRR1639771
HPB1075	Short-read archive	SRR1640205
HPB1077	Short-read archive	SRR1640000
HPB1092	Short-read archive	SRR1640007
HPB1102	Short-read archive	SRR1639767
HPB1111	Short-read archive	SRR1640136
HPB1174	Short-read archive	SRR1639774
HPB1175	Short-read archive	SRR1639778
HPB1225	Short-read archive	SRR1640172
HPB1340	Short-read archive	SRR1640144

HPB1341	Short-read archive	SRR1640146
HPB1631	Short-read archive	SRR1640221
HPB1654	Short-read archive	SRR1640175
HPB1679	Short-read archive	SRR1639790
HPB1707	Short-read archive	SRR1640208
HPB1945	Short-read archive	SRR1640116
HPB2	Short-read archive	SRR1640183
HPB2088	Nucleotide	JOKU00000000.1
HPB2129	Short-read archive	SRR1640122
HPB2130	Short-read archive	SRR1640129
HPB2134	Short-read archive	SRR1640130
HPB2141	Short-read archive	SRR1640003
HPB2166	Short-read archive	SRR1640184
HPB2263	Short-read archive	SRR1640039
HPB2264	Short-read archive	SRR1640043
HPB23	Short-read archive	SRR1639677
HPB2386	Short-read archive	SRR1640193
HPB2412	Short-read archive	SRR1640211
HPB285	Short-read archive	SRR1640177
HPB2864	Short-read archive	SRR1640182
HPB2865	Short-read archive	SRR1640187
HPB2866	Short-read archive	SRR1640188
HPB2921	Short-read archive	SRR1640173
HPB2980	Short-read archive	SRR1640195
HPB330	Short-read archive	SRR1640223
HPB37	Short-read archive	SRR1639999
HPB394	Short-read archive	SRR1640131
HPB396	Short-read archive	SRR1639755
HPB4	Short-read archive	SRR1640179
HPB424	Short-read archive	SRR1640213
HPB518	Short-read archive	SRR1640164
HPB5209	Short-read archive	SRR1640224
HPB5252	Short-read archive	SRR1640125
HPB535	Short-read archive	SRR1640222
HPB5415	Nucleotide	JOKV00000000.1
HPB5767	Short-read archive	SRR1640123
HPB6355	Short-read archive	SRR1640120
HPB7	Short-read archive	SRR1639760

HPB7171 [58]	Nucleotide	JMMW01000000.1
HPB769	Short-read archive	SRR1640108
HPB770	Short-read archive	SRR1640109
HPB771	Short-read archive	SRR1640069
HPB772	Short-read archive	SRR1640111
HPB773	Short-read archive	SRR1640089
HPB774	Short-read archive	SRR1640090
HPB8	Short-read archive	SRR1639674
HPB862	Short-read archive	SRR1639793
HPB9	Short-read archive	SRR1639763
HPB913	Short-read archive	SRR1640166
HPB960	Short-read archive	SRR1640119
HPB962	Short-read archive	SRR1639780
HPB963	Short-read archive	SRR1639787
HPB976	Short-read archive	SRR1640169
HPB1024*	Short-read archive	SRR1640197
HPB1035*	Short-read archive	SRR1640198
HPB1043*	Short-read archive	SRR1640199
HPB1050*	Short-read archive	SRR1640115
HPB1053*	Short-read archive	SRR1639663
HPB1104*	Short-read archive	SRR1640024
HPB1110*	Short-read archive	SRR1640201
HPB1342*	Short-read archive	SRR1640152
HPB1380*	Short-read archive	SRR1640162
HPB2131*	Short-read archive	SRR1686399
HPB22*	Short-read archive	SRR1639669
HPB2863*	Short-read archive	SRR1640181
HPB5622*	Short-read archive	SRR1373535

Note: Asterisks indicate taxa with high-confidence core (HCC) profiles that exactly match a profile in the HCC database.
